# Effect of High‐Pressure Processing Operating Parameters on Microbial Inactivation and Bioactive Protein Preservation in Bovine Milk: A Systematic Review

**DOI:** 10.1111/1541-4337.70324

**Published:** 2025-11-14

**Authors:** Rudy Sykora, Caleb Mark, Marie Biondi Ryan, Bishal Barman, Michael Pitino, David C. Dallas

**Affiliations:** ^1^ Department of Food Science and Technology, College of Agriculture Oregon State University Corvallis Oregon USA; ^2^ Nutrition Program, School of Nutrition and Public Health, College of Health Oregon State University Corvallis Oregon USA; ^3^ Rogers Hixon Ontario Human Milk Bank Toronto Canada

**Keywords:** cow's milk, dairy processing, food safety, food quality, protein structure

## Abstract

In the U.S., bovine milk is processed using thermal pasteurization to ensure microbial safety. However, this process alters the structure of heat‐sensitive bioactive proteins associated with the functional benefits of raw milk, including antimicrobial, immunomodulatory, and antioxidant proteins. Given the risks associated with raw milk consumption and the negative effects of thermal processing on protein functionality, there is a growing interest in high‐pressure processing (HPP), an alternative treatment that may better preserve milk's functional qualities. HPP is widely used in other food sectors but is not yet approved for milk in the U.S. Most studies have investigated either the microbial safety or the preservation of bioactive protein structure in HPP‐treated milk, rarely considering both outcomes together. Therefore, optimization of HPP treatments for dairy remains incomplete. The goal of this systematic review was to identify optimal HPP operating parameters for simultaneously achieving microbial inactivation and preserving bioactive proteins in bovine milk. Eighty‐nine articles met inclusion criteria from Web of Science, Medline, EMBASE, and PubMed based on a specified search strategy. Pressures ≥600 MPa achieved >5‐log average reductions in *Listeria monocytogenes*, *Salmonella enterica*, and *Staphylococcus aureus*, yet often caused considerable denaturation of proteins such as β‐lactoglobulin and immunoglobulin G and lesser denaturation of lactoferrin and alkaline phosphatase. Future research on HPP and bovine milk should evaluate both microbial reductions and impacts on nutrients within the same manuscript to facilitate regulatory evaluation and possible commercial adoption.

## 1 Introduction

Bovine milk provides nutritive and functional components that benefit human consumers (Haug et al. 2007). Among bovine milk's beneficial components are its functional proteins, including those with antimicrobial (lactoferrin (LF), β‐lactoglobulin (β‐LG), lactoperoxidase (LPO), α‐lactalbumin (α‐LA)), anti‐inflammatory (β‐LG, β‐casein (β‐CN), α‐LA), and immunomodulatory (LF, immunoglobulins (Ig) A, G, M) actions (Layman et al. 2018; Ogrodowczyk et al. 2022).

However, raw bovine milk can be contaminated with pathogenic and potentially life‐threatening bacteria (Vasavada 1988). To mitigate this risk, the United States Food and Drug Administration (FDA) mandates pasteurization of bovine milk destined for interstate commerce (Program, 2024a) to reduce pathogens such as *Staphylococcus aureus*, *Listeria monocytogenes* and *Escherichia coli*, which may originate from the mammary gland, udder surface or post‐processing contamination (Asfaw et al. 2023).

One of two main types of thermal pasteurization is usually employed: high‐temperature, short‐time (HTST) or low‐temperature, long‐time (LTLT). HTST is usually continuous and conducted at 72°C for >15 s (*Codes of Practice* *|*CODEXALIMENTARIUS FAO‐WHO n.d.), whereas LTLT is usually performed in a batch system and is conducted at 63°C for 30 min. Both methods cause cellular death through the denaturation of microbial DNA and key cellular structures (Ágredo‐Campos et al. 2023). Though effective for microbial safety, thermal pasteurization reduces some innate bioactive proteins via irreversible denaturation and causing aggregation (Sergius‐Ronot et al. 2022). Heat‐induced protein denaturation occurs when elevated temperatures disrupt hydrogen bonds, ionic interactions, and hydrophobic interactions, causing the protein to unfold and lose its functional activity (Stănciuc et al. 2013). Traditional thermal milk processing (HTST) denatures some heat‐sensitive whey proteins (e.g. lactoferrin, immunoglobulins, γ‐glutamyl transferase) up to 90%, reducing milk's health‐promoting potential (Čurlej et al. 2022). Additionally, both LTLT and HTST induces minor lipid oxidation (Cappozzo et al. 2015), damages milk fat globule membranes (Yang et al. 2018) and causes the production of some unwanted Maillard reaction compounds (Cosentino et al. 2016), diminishing both the nutritional value and sensory attributes of raw milk (Amador‐Espejo et al. 2014). Alternative milk processing methods that sufficiently inactivate microorganisms (>5 log reduction as per the FDA (Program 2024a)) while better preserving the concentration and function of bioactives could enable enhanced consumer benefits from milk consumption.

High pressure processing (HPP) is an alternative technology used to reduce microorganisms present in food (Billeaud 2021) while maintaining product quality. Currently, HPP is used in high moisture foods such as juices, salsas, dressings, soups, hummus, fruits, and vegetables to better preserve nutritional benefits, color and aroma compared to thermal methods (Yuan et al. 2022; Xu et al. 2009). HPP most commonly involves the applications of pressures 300–600 megapascals (MPa) for relatively brief time periods (4–15 min) at moderate temperatures (20–30°C). The lower temperatures utilized by HPP technology prevents formation of heat‐induced Maillard reaction products (e.g. bitter ketones, aldehydes) (Marousez et al. 2022). Previous studies observed that HPP improved retention of the activities and concentrations of key bioactive proteins in bovine milk at low pressures (300–400 MPa) when compared to traditional pasteurization (HTST), but did not investigate impact of these pressures on bacterial reduction (Bogahawaththa et al. 2018).

The mechanism of HPP for bacterial reduction involves inducing multiple stresses to the microbes that accumulate to eventually result in inactivation (Wu et al. 2022). The primary stressor for inactivation is the increase in pressure, causing an increase in membrane permeability, which in turn disrupts osmotic and ionic homeostasis of bacterial cells. Simultaneously, pressure causes reductions in enzymatic activity, disrupts metabolism and arrests genetic replication within the cell (Sehrawat et al. 2021). Together, these stressors lead to bacterial cell death. Although studies have illustrated the lethality of HPP on certain bacteria (Wuytack et al. 2002; Zagorska et al. 2021), it is still unclear if the pressure intensity required to achieve this lethality is consistent across literature and if these treatments have the potential to preserve the bioactivity of proteins.

At parameters required for >5‐log reductions of vegetative pathogenic bacteria, HPP may also affect milk protein structure and function. At high pressures (>400 MPa), HPP can induce water molecules to penetrate hydrophobic cavities within the protein, causing destabilization of protein secondary structures, which can lead to misfolding and protein aggregation (Hummer et al. 1998). Some protein unfolding can be reversible (Hillson et al. 1999) when the free‐energy state trends toward original beta‐sheet or alpha‐helical structures. However, some HPP‐induced changes are irreversible and result in diminished or complete loss of bioactivity (Roche and Royer 2018). Thus, it is currently unclear whether the minimum operating parameters for HPP to guarantee safe milk will also preserve protein activity.

Though prior reviews have advanced our understanding of how HPP affects bovine milk, critical limitations persist. Many reviews focus solely on the microbial efficacy of HPP (Bermúdez‐Aguirre and Barbosa‐Cánovas 2011) or the preservation of functional and nutritional components (Siddiqui et al. 2024). This bifurcation obscures critical cohesion between safety and milk protein functionality. Bioactive protein preservation is only relevant if products are safe to consume (through validation of microbial reductions). For example, papers frequently indicate preservation of key bioactive proteins at lower pressures, but other papers indicate that achieving >5‐log reductions in pathogens requires more intense HPP parameters, which could induce more degradation to milk proteins (Franco et al. 2013). Furthermore, prior reviews omitted information on key methodological variables such as operating equipment, pressurization rates or methods of analysis. This review attempts to overcome these limitations by systematically approaching the literature and comparing operating parameters’ bacterial reduction capacity and protein retention capability together. These inclusions position this review as an indispensable resource for researchers, regulatory bodies and dairy processors navigating the microbial safety and bioactive protein functionality trade‐offs inherent to HPP optimization.

## 2 Methods

**FIGURE 1:**
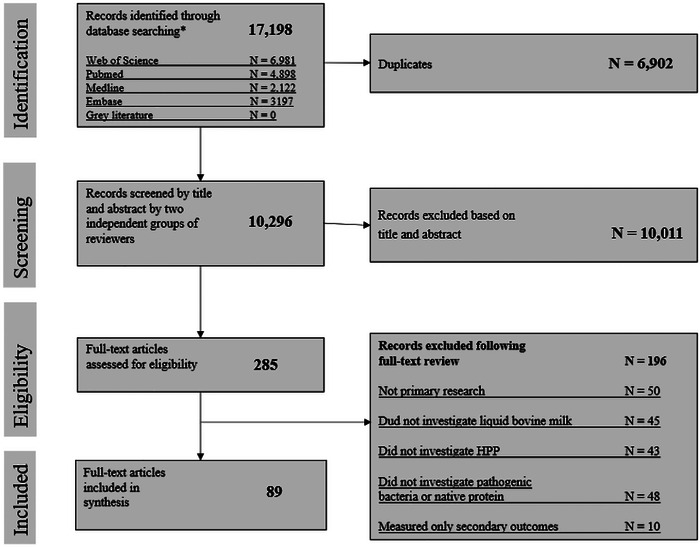
PRIMSA flow diagram showing number of papers extracted from different databases, removes as duplicates, screened by title and abstract, assessed for eligibility via full‐text review and included or excluded in the final data synthesis.

This systematic review followed the Preferred Reporting Items for systematic reviews and meta‐analyses (PRISMA) guidelines (Shamseer et al. 2015). This systematic review was registered with Open Science Framework on February 12th, 2024. The search strategy for this review was developed as part of a larger review investigating multiple non‐thermal processing techniques on bovine milk. For this article, only papers including HPP were considered, as shown in the PRISMA flow diagram (Figure 1).

### 2.1 Eligibility Criteria

Article inclusion criteria were generated using an adaptation of the Population, Exposure, Outcome (PEO) framework which guides query generation and literature search structure. Population was defined as raw, liquid bovine milk without nutritional fortifiers. Reconstituted bovine milk from milk powders or milk fractions (with the exception of fat fractions) were excluded. Fat fractionation products (whole, semi‐skim, and skim milks) were considered as they meet the standard of identity for retail fluid milk products. The exposures considered were applications of isostatic pressures on bovine milk through hydraulic or mechanical mechanisms without homogenization. Eligible outcomes constituted the analysis of microbial safety and native protein concentration or activity. Articles were included only if outcomes were analyzed before and after pressure treatment. Only peer‐reviewed, primary research articles were included. Articles from any year, language and region were considered.

### 2.2 Search Strategy

The databases PubMed, EMBASE, MEDLINE, and Web of Science were searched on February 20th, 2024, with no time restriction.

A systematic search strategy was developed and adapted to each database with the assistance of a librarian using an adapted PEO structure (Table S1). MeSH or equivalent indexed terms were used to capture all relevant articles for population (bovine milk) and exposure (high pressure processing or ultraviolet‐C processing) (note that ultraviolet C irradiation is an alternative non‐thermal process that will not be covered in this review). Outcomes (native protein quality and bacterial safety) were manually screened. Synonyms and specifics not captured using MeSH terms were identified from preliminary searches and included in final searches (Table S1).

### 2.3 Article Screening

All articles were managed through the Covidence software. Article screening was conducted by two independent reviewers. Each reviewer independently screened the title and abstract for each captured article, based on previously established eligibility criteria (Table S2). Duplicate articles were removed manually. After title and abstract screening, identified articles went through full‐text evaluation by both reviewers independently. Disputes in screening were mediated by a third, independent arbitrator and were included or excluded based on a consensus vote. Gray literature screening was conducted using Google Scholar. After initial screening of the 4 included databases was completed, reference screening was conducted with ResearchRabbit software by uploading all included articles and manually identifying any articles connected to the collection.

### 2.4 Data Extraction

A single researcher performed extraction of relevant data from included articles. The extracted information included quantitative data regarding sample collection, HPP treatment parameters (including time, vessel temperature, pressure, number of cycles, and pressurization times), equipment type, author information (including names and contact details), bacterial species, microbial methods and conditions, and protein outcomes and methodology (Table S3). The software PlotDigitizer (PORBITAL) was used to extract quantitative data that was solely provided in graphical forms.

### 2.5 Data Analysis

For comparability, all protein data were converted to percent retention, and all bacterial data was converted to lethality (log reduction in CFU/mL). All processing temperatures were converted to °C. Bacterial outcomes were analyzed through tabular, graphical and narrative analysis based on whether treatments achieved >5 log reductions as per the FDA's requirements for pasteurization of bovine fluid milk. Mixed model analysis of variance (ANOVA) was conducted using JMP Pro version 18 (SAS institute, Cary, NC) on major operating parameters (pressure, time and temperature) of HPP to compare the significance and relative impact of parameters on each microorganism.

## 3 Results and Discussion

### 3.1 Study Inclusion

Using the search strategy, 17,198 articles were captured from all databases. After the removal of duplicates (6902), 10,296 articles remained for title and abstract screening. After screening, 285 articles were identified as meeting the eligibility criteria for full‐text review. A total of 196 articles were removed during the full‐text screening based on eligibility criteria. Eighty‐nine articles were identified for inclusion in the systematic review. No additional articles were identified through gray literature searches or reference screening. The screening method is summarized in Figure 1.

### 3.2 Study Characteristics

Of the articles included, 65 investigated pathogenic bacteria (Table S4) and 24 included protein analysis (Table 2). Only 4 papers included both information on protein and bacteria (Foster et al. 2016; Guamán‐Lozada et al. 2023; Liu et al. 2020; Ramos et al. 2015). Of the 4 that evaluated the effects of HPP on both protein and bacteria, two investigated bovine colostrum (Foster et al. 2016; Gosch et al. 2014) and two investigated mature bovine milk (Liu et al. 2020; Ramos et al. 2015). The pathogens assessed included *S. aureus*, *Pseudomonas aeruginosa*, *L. monocytogenes*, *E. coli*, and *Salmonella enterica*. Proteins analyzed were IgG, LF, LPO, α‐LA, and β‐Lg. Treatments ranged from 200–600 MPa for 5–60 min at 20–40°C.

The 4 most investigated bacteria across identified literature were *L. monocytogenes* (*n* = 34), *E. coli* (*n* = 24), *S. aureus* (*n* = 19), and *S. enterica* (*n* = 8) (Table S4). Bacteria that were investigated in less than 5 unique papers include *Yersinia enterocolitica*, *Shigella flexineri*, *Campylobacter jejuni*, *Vibrio paramaemolyticus*, *Aeromonas hydrophilia*, *Francisella tularensis*, *P. aeruginosa*, and *Yersinia pseudotuberculosis* (Table S4).

Among articles which examined proteins, a single study investigated casein proteins (β‐casein), whereas all others investigated effects of pressure on whey proteins, including β‐Lg (*n* = 8), α‐LA (*n* = 6), alkaline phosphatase (ALP) (*n* = 5), IgG (*n* = 4), LF (*n* = 3), LPO (*n* = 1), sIgA (*n* = 1), XO (*n* = 1), and gamma‐glutamyl transferase (GGT) (*n* = 1) (Table 2).

Thirty‐six unique pressure treatments were investigated, including a range from 50–900 MPa, with the most common pressures being between 400–600 MPa. Treatment times ranged from 1 to 600 min, with the most common being between 5‐ and 15‐min. Treatment temperatures ranged from −20 to 75°C, with the most common being between 20 and 30°C. Pressurization and depressurization rates varied greatly depending on whether equipment used was benchtop or industrial scale, with industrial scale equipment be capable of higher pressurization rates. Pressurization and depressurization rates ranged from 1.3 to 100 MPa/s and 2.5 to 200 MPa/s, respectively. Other parameters investigated include number of pressure cycles (3 articles included; Buzrul et al. 2009; García Graells et al. 1999; Li et al. 2020) and functional additives (11 articles included; Alpas and Bozoglu 2002a, 2002b; Gao and Ju 2008; García Graells et al. 1999; García‐Graells et al. 2000; Hayman et al. 2008; Komora et al. 2020, p. 100; Liu et al. 2017; Misiou et al. 2018; Nakimbugwe et al. 2006; Tabla et al. 2012). Articles that investigated these properties (cycles and additives) were analyzed separately.

### 3.3 Effect of HPP on Bacterial Inactivation

**FIGURE 2:**
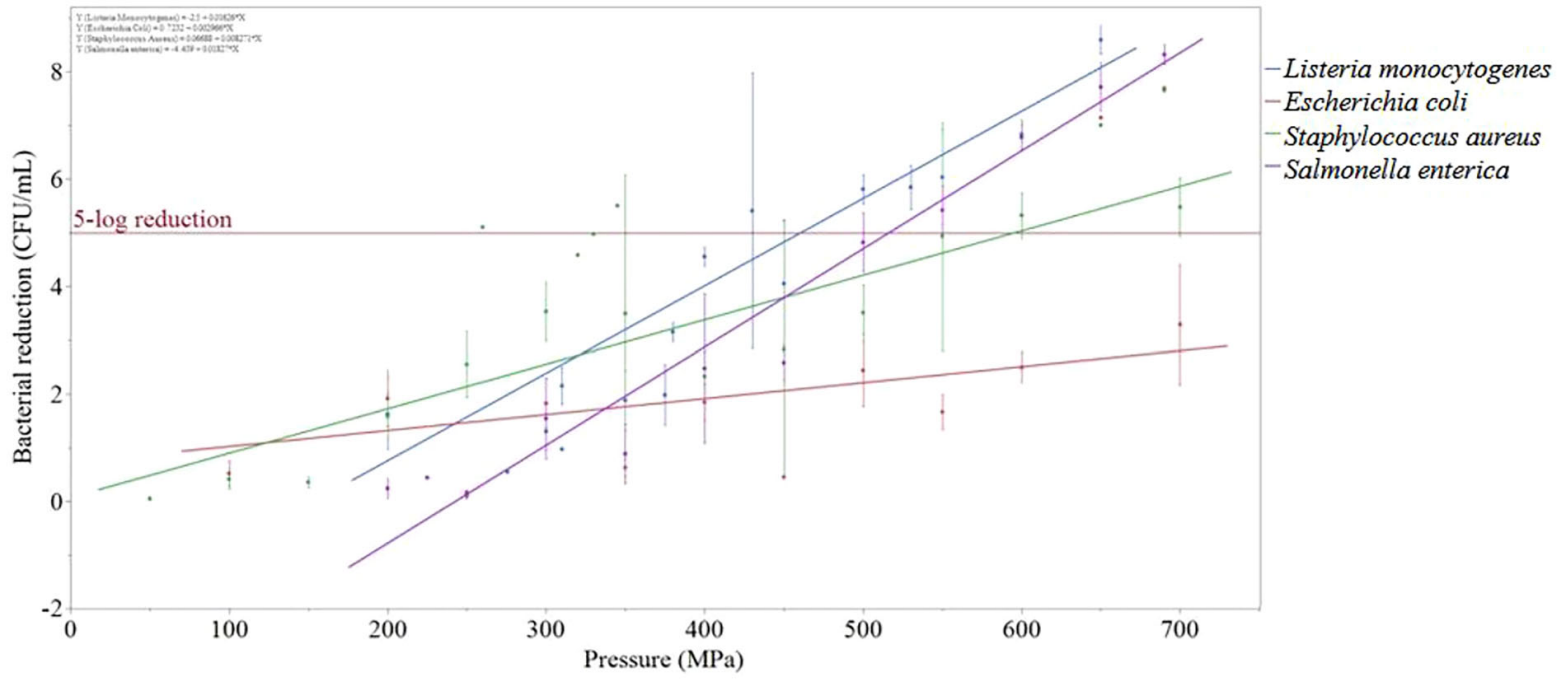
Average reduction in CFU/mL for *L. monocytogenes*, *E. coli*, *S. aureus* and *S. enterica* in whole bovine milk. Data presented as average ± SD. Linear regression line included for all bacteria.

Across all bacteria, as pressure increased, bacterial reductions increased. For the bacteria investigated in more than 5 unique papers, *L. monocytogenes* was the most pressure‐sensitive, followed by *S. enterica* and *S. aureus*, and *E. coli* was the most pressure‐resistant (Figure 2). Pressures of 450, 500, and 600 MPa were required to achieve an average >5‐log reduction of *L. monocytogenes*, *S. enterica*, and *S. aureus*, respectively (pressures are averaged across studies with different temperature and time combinations). 600 MPa also appeared to be the minimum inhibitory pressure for several *E. coli* strains (Figure 4). Notably, individual papers found >5‐log reductions in these bacteria at lower pressures than these average pressure minimums (450, 500, and 600 MPa), but typically with increased temperature and/or time treatments.

Of the bacteria that were investigated in <5 unique papers, *V. parahaemolyticus* and *A. hydrophilia* were observed to be the two most pressure‐sensitive bacteria, with >5‐log reductions, on average, occurring at pressures above 250 MPa. *P. aeruginosa*, *Y. enterocolitica*, and *Y. pseudotuberculosis* were observed to have a moderate degree of pressure resistance, as they averaged >5‐log reductions at pressures 350 MPa and above. *S. flexneri* was the most pressure‐resistant of the pathogens that were investigated in <5 articles, with average reductions >5‐log at 550 MPa and above. Among these lesser‐reported species, *V. parahaemolyticus*, *S. flexneri*, *Y. enterocolitica*, and *Y. pseudotuberculosis* can cause foodborne illness (Chen 2007; Yazdi et al. 2022) and *A. hydrophilia* and *P. aeruginosa* are associated with milk spoilage (Yerlikaya 2014).

#### 3.3.1 Listeria monocytogenes

**FIGURE 3:**
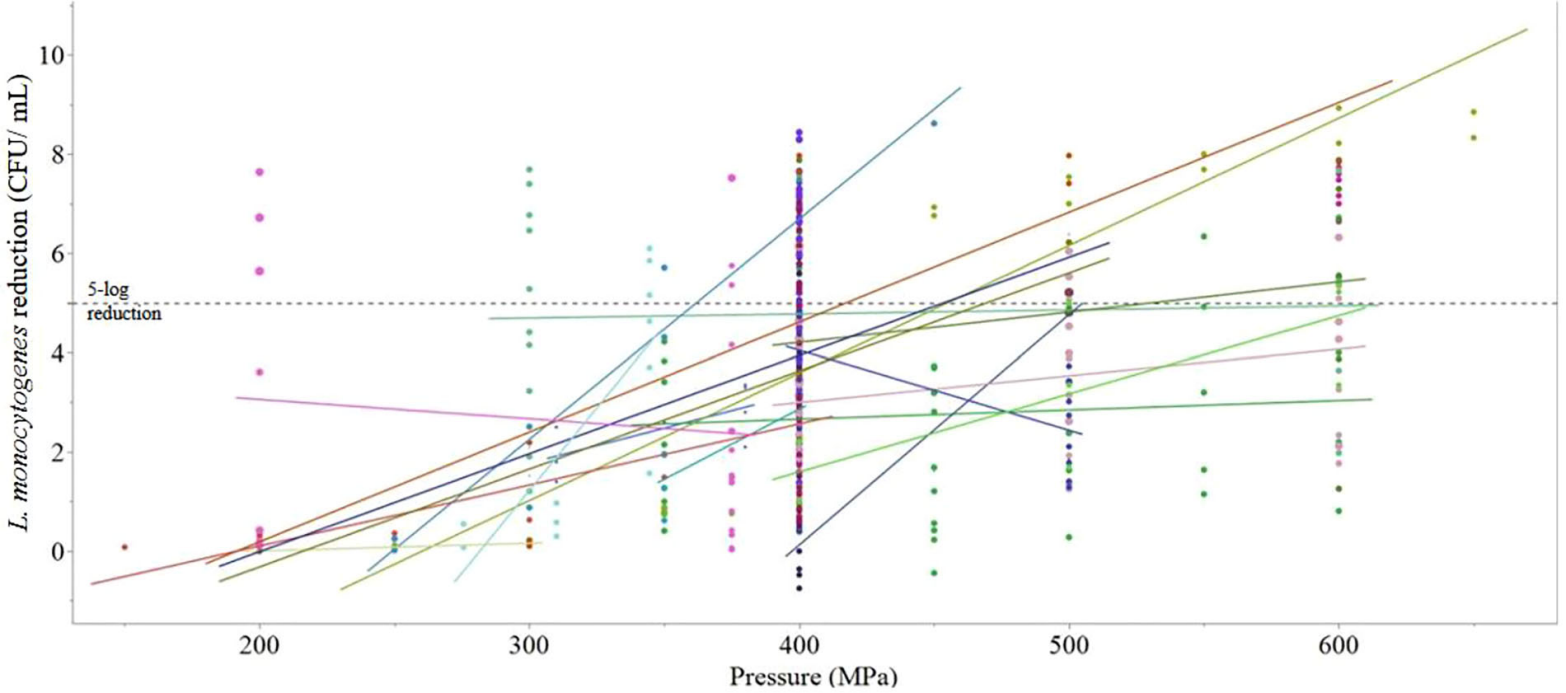
Differences between unique papers for reductions in *L. monocytogenes* (CFU/mL). Colors represent different articles, points are individual reductions (at different time and temperature combinations), and lines are mean regressions.

Overall, *L. monocytogenes* appeared to be more resistant to pressure than to temperature (Table 1 and Figure 2). Among the included studies, there was ample variation in minor parameters, resulting in the inter‐study observed differences in *L. monocytogenes*’s response to pressure (Figure 3).

**TABLE 1:** Impact of operating parameters on reduction of protein and specific bacteria. *P* < 0.05 represents significant contribution to reduction, *F*‐ratio measures relative impact of parameter on reduction. DF are degrees of freedom.

Parameter	*F*‐ratio	*P*‐value
*Listeria monocytogenes* (DF = 250)		
Pressure (MPa)	171.12	<0.0001
Temperature (°C)	15.14	0.0001
Time (min)	11.81	0.0007

*Escherichia coli* (DF = 194)		
Pressure (MPa)	22.28	<0.0001
Temperature (°C)	16.65	<0.0001
Time (min)	5.006	<0.0001

*Staphylococcus aureus* (DF = 164)		
Pressure (MPa)	87.73	<0.0001
Temperature (°C)	25.21	<0.0001
Time (min)	0.26	0.06139

Protein (DF = 198)		
Pressure (MPa)	52.69	<0.0001
Temperature (°C)	13.9	0.0003
Time (min)	3.64	0.0589

**TABLE 2:** Percent retention of protein due to HPP treatments across literature. NR* are values not reported by authors.

**Pressure (MPa)**	**Time (min)**	**Holding temperature (°C)**	**Percent retention (%)**	**Assay type**	**(First author year)**
**Alkaline phosphatase**					
200	10	10	94.98	Fluorometric substrate assay	(Balci 2002)
200	10	10	91.21	Fluorometric substrate assay	(Balci 2002)
200	15	NR*	99	Rapid colorimetric test	(Mussa 1997)
200	30	NR*	98.5	Rapid colorimetric test	(Mussa 1997)
200	45	NR*	97.5	Rapid colorimetric test	(Mussa 1997)
200	60	NR*	97.5	Rapid colorimetric test	(Mussa 1997)
250	20	NR*	98.5	Rapid colorimetric test	(Mussa 1997)
250	40	NR*	97.5	Rapid colorimetric test	(Mussa 1997)
250	55	NR*	96.5	Rapid colorimetric test	(Mussa 1997)
350	5	NR*	99.5	Rapid colorimetric test	(Mussa 1997)
350	13	NR*	98.5	Rapid colorimetric test	(Mussa 1997)
350	28	NR*	97.5	Rapid colorimetric test	(Mussa 1997)
400	5	NR*	99	Rapid colorimetric test	(Mussa 1997)
400	10	10	45.02	Fluorometric substrate assay	(Balci 2002)
400	10	10	37.95	Fluorometric substrate assay	(Balci 2002)
400	10	NR*	72.1	NR*	(Koncz 2007)
400	10	NR*	98.5	Rapid colorimetric test	(Mussa 1997)
400	15	NR*	97.5	Rapid colorimetric test	(Mussa 1997)
400	20	NR*	25.24	NR*	(Koncz 2007)
400	20	NR*	96.5	Rapid colorimetric test	(Mussa 1997)
400	40	NR*	13.91	NR*	(Koncz 2007)
500	2	5	93.37	Fluorometric substrate assay	(Periera 2014)
500	4	5	90.78	Fluorometric substrate assay	(Periera 2014)
500	8	5	78.96	Fluorometric substrate assay	(Periera 2014)
500	10	10	30.86	Fluorometric substrate assay	(Balci 2002)
500	10	10	26.16	Fluorometric substrate assay	(Balci 2002)
500	10	10	18.38	Fluorometric substrate assay	(Balci 2002)
500	10	NR*	62.83	NR*	(Koncz 2007)
500	16	5	70.32	Fluorometric substrate assay	(Periera 2014)
500	20	NR*	14.94	NR*	(Koncz 2007)
500	30	10	16.49	Fluorometric substrate assay	(Balci 2002)
500	32	5	66.57	Fluorometric substrate assay	(Periera 2014)
500	40	NR*	8.24	NR*	(Koncz 2007)
500	50	10	15.46	Fluorometric substrate assay	(Balci 2002)
500	64	5	59.08	Fluorometric substrate assay	(Periera 2014)
500	128	5	54.18	Fluorometric substrate assay	(Periera 2014)
550	2	5	85.59	Fluorometric substrate assay	(Periera 2014)
550	4	5	73.78	Fluorometric substrate assay	(Periera 2014)
550	8	5	74.06	Fluorometric substrate assay	(Periera 2014)
550	10	10	14.95	Fluorometric substrate assay	(Balci 2002)
550	16	5	66.86	Fluorometric substrate assay	(Periera 2014)
550	30	10	10.14	Fluorometric substrate assay	(Balci 2002)
550	32	5	57.35	Fluorometric substrate assay	(Periera 2014)
550	50	10	11.86	Fluorometric substrate assay	(Balci 2002)
550	64	5	45.82	Fluorometric substrate assay	(Periera 2014)
550	128	5	30.55	Fluorometric substrate assay	(Periera 2014)
600	2	5	79.25	Fluorometric substrate assay	(Periera 2014)
600	4	5	71.18	Fluorometric substrate assay	(Periera 2014)
600	8	5	67.7	Fluorometric substrate assay	(Periera 2014)
600	10	10	24.98	Fluorometric substrate assay	(Balci 2002)
600	10	10	16.03	Fluorometric substrate assay	(Balci 2002)
600	10	NR*	92.83	NR*	(Koncz 2007)
600	10	NR*	9.79	NR*	(Koncz 2007)
600	15	25	54.97666667	Elisa	(Yu 2022)
600	16	5	53.31	Fluorometric substrate assay	(Periera 2014)
600	20	NR*	66.42	NR*	(Koncz 2007)
600	20	NR*	6.18	NR*	(Koncz 2007)
600	32	5	41.79	Fluorometric substrate assay	(Periera 2014)
600	40	NR*	26.42	NR*	(Koncz 2007)
600	40	NR*	1.03	NR*	(Koncz 2007)
600	64	5	23.05	Fluorometric substrate assay	(Periera 2014)
600	128	5	7.49	Fluorometric substrate assay	(Periera 2014)
650	2	5	57.93	Fluorometric substrate assay	(Periera 2014)
650	4	5	49.57	Fluorometric substrate assay	(Periera 2014)
650	8	5	45.53	Fluorometric substrate assay	(Periera 2014)
650	16	5	36.02	Fluorometric substrate assay	(Periera 2014)
650	32	5	12.97	Fluorometric substrate assay	(Periera 2014)
650	64	5	3.17	Fluorometric substrate assay	(Periera 2014)
650	128	5	0.86	Fluorometric substrate assay	(Periera 2014)
700	2	5	50.14	Fluorometric substrate assay	(Periera 2014)
700	4	5	37.18	Fluorometric substrate assay	(Periera 2014)
700	8	5	27.67	Fluorometric substrate assay	(Periera 2014)
700	10	10	17.21	Fluorometric substrate assay	(Balci 2002)
700	10	10	12.73	Fluorometric substrate assay	(Balci 2002)
700	10	NR*	17.74	NR*	(Koncz 2007)
700	16	5	14.99	Fluorometric substrate assay	(Periera 2014)
700	20	NR*	16.98	NR*	(Koncz 2007)
700	32	5	4.61	Fluorometric substrate assay	(Periera 2014)
700	40	NR*	14.72	NR*	(Koncz 2007)
700	60	NR*	7.17	NR*	(Koncz 2007)
700	64	5	2.31	Fluorometric substrate assay	(Periera 2014)
750	2	5	44.38	Fluorometric substrate assay	(Periera 2014)
750	4	5	27.09	Fluorometric substrate assay	(Periera 2014)
750	8	5	17	Fluorometric substrate assay	(Periera 2014)
750	16	5	9.22	Fluorometric substrate assay	(Periera 2014)
750	32	5	2.02	Fluorometric substrate assay	(Periera 2014)
750	64	5	0.58	Fluorometric substrate assay	(Periera 2014)
800	2	5	42.07	Fluorometric substrate assay	(Periera 2014)
800	4	5	27.09	Fluorometric substrate assay	(Periera 2014)
800	8	5	11.24	Fluorometric substrate assay	(Periera 2014)
800	10	10	16.5	Fluorometric substrate assay	(Balci 2002)
800	10	10	11.55	Fluorometric substrate assay	(Balci 2002)
800	16	5	4.61	Fluorometric substrate assay	(Periera 2014)
800	32	5	1.44	Fluorometric substrate assay	(Periera 2014)
800	64	5	0	Fluorometric substrate assay	(Periera 2014)
**α‐lactalbumin**					
100	3	70	78.44	HPLC	(Hinrichs 2005)
100	6	70	77.25	HPLC	(Hinrichs 2005)
100	9	70	64.97	HPLC	(Hinrichs 2005)
100	15	25	100	HPLC	(Lopez‐Fandino 1998)
100	15	40	100	HPLC	(Lopez‐Fandino 1998)
100	15	50	100	HPLC	(Lopez‐Fandino 1998)
100	15	70	61.38	HPLC	(Hinrichs 2005)
100	18	70	58.68	HPLC	(Hinrichs 2005)
100	24	70	57.19	HPLC	(Hinrichs 2005)
200	15	40	100	HPLC	(Lopez‐Fandino 1998)
200	15	50	100	HPLC	(Lopez‐Fandino 1998)
200	15	60	96.58	HPLC	(Lopez‐Fandino 1998)
300	15	40	100	HPLC	(Lopez‐Fandino 1998)
300	15	50	100	HPLC	(Lopez‐Fandino 1998)
300	15	60	85.27	HPLC	(Lopez‐Fandino 1998)
400	15	25	100	HPLC	(Garcia‐Risco 2000)
400	15	40	97	HPLC	(Garcia‐Risco 2000)
400	15	40	95.89	HPLC	(Lopez‐Fandino 1998)
400	15	50	85	HPLC	(Garcia‐Risco 2000)
400	15	50	89.04	HPLC	(Lopez‐Fandino 1998)
400	15	60	42	HPLC	(Garcia‐Risco 2000)
400	15	60	42.81	HPLC	(Lope‐Fandino 1998)
500	3	30	94.45	HPLC	(Hinrichs 2005)
500	3	30	93.11	HPLC	(Hinrichs 2005)
500	3	60	32.34	HPLC	(Hinrichs 2005)
500	6	30	95.55	HPLC	(Hinrichs 2005)
500	6	30	94.01	HPLC	(Hinrichs 2005)
500	6	60	26.65	HPLC	(Hinrichs 2005)
500	9	30	100.11	HPLC	(Hinrichs 2005)
500	9	30	100	HPLC	(Hinrichs 2005)
500	9	60	26.05	HPLC	(Hinrichs 2005)
500	11	30	98.9	HPLC	(Hinrichs 2005)
500	12	30	99.71	HPLC	(Hinrichs 2005)
500	12	60	19.46	HPLC	(Hinrichs 2005)
500	15	30	97.99	HPLC	(Hinrichs 2005)
500	15	30	97	HPLC	(Hinrichs 2005)
500	15	60	18.86	HPLC	(Hinrichs 2005)
500	18	30	108.9	HPLC	(Hinrichs 2005)
500	18	30	107.49	HPLC	(Hinrichs 2005)
500	20	30	108.05	HPLC	(Hinrichs 2005)
500	21	30	106.89	HPLC	(Hinrichs 2005)
500	21	60	17.37	HPLC	(Hinrichs 2005)
500	24	30	104.02	HPLC	(Hinrichs 2005)
500	24	30	103.29	HPLC	(Hinrichs 2005)
500	24	60	17.07	HPLC	(Hinrichs 2005)
500	32	30	103.16	HPLC	(Hinrichs 2005)
500	32	30	102.4	HPLC	(Hinrichs 2005)
500	32	60	16.17	HPLC	(Hinrichs 2005)
600	3	10	94.91	HPLC	(Hinrichs 2005)
600	3	50	64.97	HPLC	(Hinrichs 2005)
600	5	40	93.7	LC/MS Q‐ToF	(Liu 2020)
600	6	50	58.98	HPLC	(Hinrichs 2005)
600	9	10	95.5	HPLC	(Hinrichs 2005)
600	9	50	56.29	HPLC	(Hinrichs 2005)
600	12	10	92.51	HPLC	(Hinrichs 2005)
600	12	50	45.29	HPLC	(Hinrichs 2005)
600	15	10	91.02	HPLC	(Hinrichs 2005)
600	15	20	94.31	Radial immunodiffusion assay	(Ramos 2015)
600	15	50	41.32	HPLC	(Hinrichs 2005)
600	18	10	91.32	HPLC	(Hinrichs 2005)
600	18	50	41.62	HPLC	(Hinrichs 2005)
600	21	50	36.53	HPLC	(Hinrichs 2005)
600	24	10	88.62	HPLC	(Hinrichs 2005)
600	24	50	36.53	HPLC	(Hinrichs 2005)
600	32	10	87.43	HPLC	(Hinrichs 2005)
600	32	50	32.04	HPLC	(Hinrichs 2005)
**β‐casein**					
400	15	25	89.81	HPLC	(Garcio‐Risco 2000)
400	15	40	92.28	HPLC	(Garcio‐Risco 2000)
400	15	50	98.15	HPLC	(Garcio‐Risco 2000)
400	15	60	99.16	HPLC	(Garcio‐Risco 2000)
**β‐lactoglobulin**					
100	15	40	99.66	HPLC	(Lopez‐Fandino 1998)
100	15	50	99.66	HPLC	(Lopez‐Fandino 1998)
100	15	60	97.31	HPLC	(Lopez‐Fandino 1998)
100	15	60	99.32	HPLC	(Lopez‐Fandino 1998)
100	30	25	98.06357022	HPLC	(Lopez‐Fandino 1996)
200	10	25	83.59971042	HPLC	(Lopez‐Fandino 1996)
200	15	25	92.93	HPLC	(Lopez‐Fandino 1998)
200	15	40	87.21	HPLC	(Lopez‐Fandino 1998)
200	15	50	78.11	HPLC	(Lopez‐Fandino 1998)
200	15	60	36.03	HPLC	(Lopez‐Fandino 1998)
200	20	25	79.10285682	HPLC	(Lopez‐Fandino 1996)
200	30	25	70.35602645	HPLC	(Lopez‐Fandino 1996)
200	30	25	72.2225316	HPLC	(Lopez‐Fandino 1996)
200	60	25	60.55577212	HPLC	(Lopez‐Fandino 1996)
300	3	30	66.02	HPLC	(Hinrichs 2005)
300	15	25	48.15	HPLC	(Lopez‐Fandino 1998)
300	15	30	33	HPLC	(Hinrichs 2005)
300	15	40	19.19	HPLC	(Lopez‐Fandino 1998)
300	15	50	9.09	HPLC	(Lopez‐Fandino 1998)
300	15	60	5.39	HPLC	(Lopez‐Fandino 1998)
300	18	30	29.61	HPLC	(Hinrichs 2005)
300	21	30	25.49	HPLC	(Hinrichs 2005)
300	24	30	22.82	HPLC	(Hinrichs 2005)
300	30	25	21.3062602	HPLC	(Lopez‐Fandino 1996)
300	32	30	10.68	HPLC	(Hinrichs 2005)
400	3	30	40.05	HPLC	(Hinrichs 2005)
400	6	30	28.64	HPLC	(Hinrichs 2005)
400	9	30	16.5	HPLC	(Hinrichs 2005)
400	10	25	24.338698	HPLC	(Lopez‐Fandino 1996)
400	12	30	17.23	HPLC	(Hinrichs 2005)
400	14	30	8.74	HPLC	(Hinrichs 2005)
400	15	25	24	HPLC	(Garcio‐Risco 2000)
400	15	25	25.25	HPLC	(Lopez‐Fandino 1998)
400	15	30	14.08	HPLC	(Hinrichs 2005
400	15	40	5	HPLC	(Garcio‐Risco 2000)
400	15	40	5.05	HPLC	(Lopez‐Fandino 1998)
400	15	50	4	HPLC	(Garcio‐Risco 2000)
400	15	50	5.05	HPLC	(Lopez‐Fandino 1998)
400	15	60	3	HPLC	(Garcio‐Risco 2000)
400	15	60	3.37	HPLC	(Lopez‐Fandino 1998)
400	18	30	11.65	HPLC	(Hinrichs 2005)
400	20	25	13.22882441	HPLC	(Lopez‐Fandino 1996)
400	21	30	9.95	HPLC	(Hinrichs 2005)
400	30	25	11.62134499	HPLC	(Lopez‐Fandino 1996)
400	30	25	10.58083199	HPLC	(Lopez‐Fandino 1996)
400	32	30	5.83	HPLC	(Hinrichs 2005)
400	60	25	7.671103191	HPLC	(Lopez‐Fandino 1996)
450	3	20	89.7	ELISA	(Mazri 2012)
450	5	20	86.45	ELISA	(Mazri 2012)
450	10	20	77.24	ELISA	(Mazri 2012)
450	15	20	62.06	ELISA	(Mazri 2012)
450	20	20	53.66	ELISA	(Mazri 2012)
450	25	20	46.34	ELISA	(Mazri 2012)
450	30	20	39.57	ELISA	(Mazri 2012)
500	3	1	65.31	HPLC	(Hinrichs 2005)
500	3	10	61.24	HPLC	(Hinrichs 2005)
500	3	20	48.33	HPLC	(Hinrichs 2005)
500	3	30	14.94	HPLC	(Hinrichs 2005)
500	3	30	27.59	HPLC	(Hinrichs 2005)
500	3	30	27.59	HPLC	(Hinrichs 2005)
500	3	30	27.91	HPLC	(Hinrichs 2005)
500	3	30	27.75	HPLC	(Hinrichs 2005)
500	3	40	9.57	HPLC	(Hinrichs 2005)
500	3	50	4.45	HPLC	(Hinrichs 2005)
500	3	60	2.15	HPLC	(Hinrichs 2005)
500	5	20	57.45	ELISA	(Mazri 2012)
500	6	10	61	HPLC	(Hinrichs 2005)
500	6	20	42.58	HPLC	(Hinrichs 2005)
500	6	40	7.18	HPLC	(Hinrichs 2005)
500	6	50	2.87	HPLC	(Hinrichs 2005)
500	6	60	1.67	HPLC	(Hinrichs 2005)
500	9	1	61.48	HPLC	(Hinrichs 2005)
500	9	10	56.22	HPLC	(Hinrichs 2005)
500	9	20	35.17	HPLC	(Hinrichs 2005)
500	9	40	5.02	HPLC	(Hinrichs 2005)
500	9	50	2.39	HPLC	(Hinrichs 2005)
500	9	60	1.2	HPLC	(Hinrichs 2005)
500	10	20	46.61	ELISA	(Mazri 2012)
500	12	1	58.61	HPLC	(Hinrichs 2005)
500	12	10	54.54	HPLC	(Hinrichs 2005)
500	12	20	30.38	HPLC	(Hinrichs 2005)
500	12	40	3.35	HPLC	(Hinrichs 2005)
500	12	50	2.15	HPLC	(Hinrichs 2005)
500	12	60	0.96	HPLC	(Hinrichs 2005)
500	15	1	54.31	HPLC	(Hinrichs 2005)
500	15	10	54.54	HPLC	(Hinrichs 2005)
500	15	20	41.19	ELISA	(Mazri 2012)
500	15	20	28.47	HPLC	(Hinrichs 2005)
500	15	30	4.02	HPLC	(Hinrichs 2005)
500	15	30	7.47	HPLC	(Hinrichs 2005)
500	15	30	7.47	HPLC	(Hinrichs 2005)
500	15	30	8.01	HPLC	(Hinrichs 2005)
500	15	30	7.66	HPLC	(Hinrichs 2005)
500	15	40	2.87	HPLC	(Hinrichs 2005)
500	15	50	1.91	HPLC	(Hinrichs 2005)
500	15	60	0.96	HPLC	(Hinrichs 2005)
500	18	1	55.74	HPLC	(Hinrichs 2005)
500	18	10	54.07	HPLC	(Hinrichs 2005)
500	18	20	26.08	HPLC	(Hinrichs 2005)
500	18	30	4.02	HPLC	(Hinrichs 2005)
500	18	30	7.47	HPLC	(Hinrichs 2005)
500	18	30	7.47	HPLC	(Hinrichs 2005)
500	18	30	7.77	HPLC	(Hinrichs 2005)
500	18	30	7.18	HPLC	(Hinrichs 2005)
500	18	40	2.15	HPLC	(Hinrichs 2005)
500	18	50	1.44	HPLC	(Hinrichs 2005)
500	18	60	0.96	HPLC	(Hinrichs 2005)
500	20	20	34.96	ELISA	(Mazri 2012)
500	21	1	52.63	HPLC	(Hinrichs 2005)
500	21	10	51.67	HPLC	(Hinrichs 2005)
500	21	20	22	HPLC	(Hinrichs 2005)
500	21	30	3.45	HPLC	(Hinrichs 2005)
500	21	30	6.32	HPLC	(Hinrichs 2005)
500	21	30	6.32	HPLC	(Hinrichs 2005)
500	21	30	6.31	HPLC	(Hinrichs 2005)
500	21	30	6.22	HPLC	(Hinrichs 2005)
500	21	40	2.39	HPLC	(Hinrichs 2005)
500	21	50	1.2	HPLC	(Hinrichs 2005)
500	21	60	0.48	HPLC	(Hinrichs 2005)
500	24	1	56.22	HPLC	(Hinrichs 2005)
500	24	10	53.11	HPLC	(Hinrichs 2005)
500	24	20	20.33	HPLC	(Hinrichs 2005)
500	24	30	2.3	HPLC	(Hinrichs 2005)
500	24	30	4.89	HPLC	(Hinrichs 2005)
500	24	30	4.89	HPLC	(Hinrichs 2005)
500	24	30	5.1	HPLC	(Hinrichs 2005)
500	24	30	5.02	HPLC	(Hinrichs 2005)
500	24	40	2.39	HPLC	(Hinrichs 2005)
500	24	50	1.2	HPLC	(Hinrichs 2005)
500	24	60	0.48	HPLC	(Hinrichs 2005)
500	25	20	28.18	ELISA	(Mazri 2012)
500	30	20	22.22	ELISA	(Mazri 2012)
500	32	1	46.41	HPLC	(Hinrichs 2005)
500	32	10	42.12	HPLC	(Hinrichs 2005)
500	32	20	14.35	HPLC	(Hinrichs 2005)
500	32	30	2.01	HPLC	(Hinrichs 2005)
500	32	30	3.45	HPLC	(Hinrichs 2005)
500	32	30	3.45	HPLC	(Hinrichs 2005)
500	32	30	2.91	HPLC	(Hinrichs 2005)
500	32	30	4.07	HPLC	(Hinrichs 2005)
500	32	40	1.44	HPLC	(Hinrichs 2005)
500	32	50	1.2	HPLC	(Hinrichs 2005)
500	32	60	0.48	HPLC	(Hinrichs 2005)
550	2	20	69.11	ELISA	(Mazri 2012)
550	4	20	54.74	ELISA	(Mazri 2012)
550	6	20	47.43	ELISA	(Mazri 2012)
550	8	20	44.17	ELISA	(Mazri 2012)
550	10	20	38.48	ELISA	(Mazri 2012)
550	15	20	27.37	ELISA	(Mazri 2012)
550	20	20	19.24	ELISA	(Mazri 2012)
600	3	20	55.28	ELISA	(Mazri 2012)
600	3	30	19.17	HPLC	(Hinrichs 2005)
600	5	40	50	LC/MS Q‐ToF	(Liu 2020)
600	5	40	59	LC/MS Q‐ToF	(Liu 2020)
600	6	20	38.48	ELISA	(Mazri 2012)
600	8	20	32.25	ELISA	(Mazri 2012)
600	10	20	26.83	ELISA	(Mazri 2012)
600	12	30	11.65	HPLC	(Hinrichs 2005)
600	15	20	19.51	ELISA	(Mazri 2012)
600	15	20	20.05	Radial immunodiffusion assay	(Ramos 2015)
600	15	25	76.51451303	ELISA	(Yu 2022)
600	15	30	5.83	HPLC	(Hinrichs 2005)
600	18	30	4.61	HPLC	(Hinrichs 2005)
600	21	30	4.13	HPLC	(Hinrichs 2005)
600	24	30	3.64	HPLC	(Hinrichs 2005)
600	32	30	1.94	HPLC	(Hinrichs 2005)
650	2	20	65.31	ELISA	(Mazri 2012)
650	4	20	49.32	ELISA	(Mazri 2012)
650	6	20	34.96	ELISA	(Mazri 2012)
650	8	20	32.25	ELISA	(Mazri 2012)
650	10	20	27.1	ELISA	(Mazri 2012)
650	12	20	22.76	ELISA	(Mazri 2012)
650	15	20	17.62	ELISA	(Mazri 2012)
700	1	20	78.05	ELISA	(Mazri 2012)
700	3	20	47.15	ELISA	(Mazri 2012)
700	5	20	37.13	ELISA	(Mazri 2012)
700	7	20	32.25	ELISA	(Mazri 2012)
700	10	20	23.85	ELISA	(Mazri 2012)
700	15	20	14.36	ELISA	(Mazri 2012)
800	3	30	10.68	HPLC	(Hinrichs 2005)
800	12	30	4.85	HPLC	(Hinrichs 2005)
800	15	30	4.13	HPLC	(Hinrichs 2005)
800	18	30	3.64	HPLC	(Hinrichs 2005)
800	21	30	3.16	HPLC	(Hinrichs 2005)
800	24	30	2.91	HPLC	(Hinrichs 2005)
800	32	30	1.94	HPLC	(Hinrichs 2005)
**γ‐glutamlytransferase**					
400	2	20	92.88	Fluorometric substrate assay	(Rademacher 2006)
400	4	20	99.43	Fluorometric substrate assay	(Rademacher 2006)
400	8	20	86.04	Fluorometric substrate assay	(Rademacher 2006)
400	16	20	91.17	Fluorometric substrate assay	(Rademacher 2006)
400	32	20	81.2	Fluorometric substrate assay	(Rademacher 2006)
400	64	20	74.93	Fluorometric substrate assay	(Rademacher 2006)
400	128	20	66.1	Fluorometric substrate assay	(Rademacher 2006)
500	2	20	96.01	Fluorometric substrate assay	(Rademacher 2006)
500	4	20	87.46	Fluorometric substrate assay	(Rademacher 2006)
500	8	20	72.08	Fluorometric substrate assay	(Rademacher 2006)
500	16	20	73.5	Fluorometric substrate assay	(Rademacher 2006)
500	32	20	50.14	Fluorometric substrate assay	(Rademacher 2006)
500	64	20	15.95	Fluorometric substrate assay	(Rademacher 2006)
500	128	20	0	Fluorometric substrate assay	(Rademacher 2006)
550	2	20	63.53	Fluorometric substrate assay	(Rademacher 2006)
550	2	20	18.8	Fluorometric substrate assay	(Rademacher 2006)
550	4	20	84.33	Fluorometric substrate assay	(Rademacher 2006)
550	4	20	42.74	Fluorometric substrate assay	(Rademacher 2006)
550	4	20	8.83	Fluorometric substrate assay	(Rademacher 2006)
550	8	20	53.56	Fluorometric substrate assay	(Rademacher 2006)
550	8	20	8.83	Fluorometric substrate assay	(Rademacher 2006)
550	8	20	3.99	Fluorometric substrate assay	(Rademacher 2006)
550	16	20	23.65	Fluorometric substrate assay	(Rademacher 2006)
550	16	20	2.85	Fluorometric substrate assay	(Rademacher 2006)
550	32	20	6.27	Fluorometric substrate assay	(Rademacher 2006)
550	32	20	0	Fluorometric substrate assay	(Rademacher 2006)
550	32	20	0.28	Fluorometric substrate assay	(Rademacher 2006)
550	64	20	0.28	Fluorometric substrate assay	(Rademacher 2006)
**Immunoglobulin G**					
300	30	20	102.719887	Radial immunodiffusion assay	(Foster 2016)
300	45	20	103.4263511	Radial immunodiffusion assay	(Foster 2016)
300	60	20	100.3532321	Radial immunodiffusion assay	(Foster 2016)
400	10	20	98.97562699	Radial immunodiffusion assay	(Foster 2016)
400	10	20	62.78	ELISA	(Gosch 2014)
400	10	20	54.54	ELISA	(Gosch 2014)
400	10	20	100	ELISA	(Gosch 2014)
400	10	20	87.22929225	ELISA	(Gosch 2014)
400	15	20	91.8050159	Radial immunodiffusion assay	(Foster 2016)
400	15	30	59	ELISA	(Bogahwaththa 2018)
400	20	20	87.70752384	Radial immunodiffusion assay	(Foster 2016)
500	10	20	26.99	ELISA	(Gosch 2014)
500	10	20	25	ELISA	(Gosch 2014)
500	10	20	32.69803454	ELISA	(Gosch 2014)
500	10	20	36.7716008	ELISA	(Gosch 2014)
500	15	30	24	ELISA	(Bogahwaththa 2018)
600	15	20	57.02	Spectrophotometer	(Ramos 2015)
600	15	30	4	ELISA	(Bogahwaththa 2018)
**Lactoferrin**					
250	5	15	100	ELISA	(Bravo 2015)
450	5	15	100	ELISA	(Bravo 2015)
450	5	20	100	ELISA	(Mazri 2012)
450	10	20	98.26	ELISA	(Mazri 2012)
450	15	20	98.96	ELISA	(Mazri 2012)
450	20	20	97.92	ELISA	(Mazri 2012)
450	25	20	95.14	ELISA	(Mazri 2012)
450	30	20	93.4	ELISA	(Mazri 2012)
450	40	20	90.97	ELISA	(Mazri 2012)
500	5	20	100	ELISA	(Mazri 2012)
500	10	20	98.96	ELISA	(Mazri 2012)
500	15	20	96.87	ELISA	(Mazri 2012)
500	20	20	96.18	ELISA	(Mazri 2012)
500	25	20	91.67	ELISA	(Mazri 2012)
500	30	20	89.58	ELISA	(Mazri 2012)
550	1.8	20	97.57	ELISA	(Mazri 2012)
550	5	15	78.57142857	ELISA	(Bravo 2015)
550	6	20	93.06	ELISA	(Mazri 2012)
550	8	20	93.4	ELISA	(Mazri 2012)
550	10	20	92.36	ELISA	(Mazri 2012)
550	15	20	88.54	ELISA	(Mazri 2012)
550	20	20	79.51	ELISA	(Mazri 2012)
550	25	20	72.57	ELISA	(Mazri 2012)
600	3	20	95.49	ELISA	(Mazri 2012)
600	6	20	87.85	ELISA	(Mazri 2012)
600	10	20	79.17	ELISA	(Mazri 2012)
600	15	20	75.35	ELISA	(Mazri 2012)
600	15	20	30.39	ELISA	(Ramos 2015)
600	20	20	73.61	ELISA	(Mazri 2012)
600	25	20	61.46	ELISA	(Mazri 2012)
650	4	20	94.44	ELISA	(Mazri 2012)
650	6	20	87.15	ELISA	(Mazri 2012)
650	8	20	84.72	ELISA	(Mazri 2012)
650	10	20	75.35	ELISA	(Mazri 2012)
650	12	20	67.71	ELISA	(Mazri 2012)
650	15	20	63.89	ELISA	(Mazri 2012)
700	1	20	89.93	ELISA	(Mazri 2012)
700	3	20	83.68	ELISA	(Mazri 2012)
700	5	15	21.42857143	ELISA	(Bravo 2015)
700	5	20	78.47	ELISA	(Mazri 2012)
700	7	20	73.61	ELISA	(Mazri 2012)
700	10	20	47.92	ELISA	(Mazri 2012)
700	15	20	32.99	ELISA	(Mazri 2012)
800	5	15	7.142857143	ELISA	(Bravo 2015)
900	5	15	7.142857143	ELISA	(Bravo 2015)
**Lactoperoxidase**					
600	15	20	101.2	Radial immunodiffusion assay	(Ramos 2015)
**Phosphohexoseisomerase**					
420	2	20	79.34	Fluorometric substrate assay	(Rademacher 2006)
420	4	20	61.97	Fluorometric substrate assay	(Rademacher 2006)
420	8	20	56.57	Fluorometric substrate assay	(Rademacher 2006)
420	16	20	29.34	Fluorometric substrate assay	(Rademacher 2006)
420	32	20	42.02	Fluorometric substrate assay	(Rademacher 2006)
460	2	20	51.17	Fluorometric substrate assay	(Rademacher 2006)
460	2	20	8.45	Fluorometric substrate assay	(Rademacher 2006)
460	4	20	26.53	Fluorometric substrate assay	(Rademacher 2006)
460	4	20	7.98	Fluorometric substrate assay	(Rademacher 2006)
460	8	20	31.46	Fluorometric substrate assay	(Rademacher 2006)
460	8	20	4.46	Fluorometric substrate assay	(Rademacher 2006)
460	16	20	25.35	Fluorometric substrate assay	(Rademacher 2006)
460	16	20	0	Fluorometric substrate assay	(Rademacher 2006)
460	32	20	16.9	Fluorometric substrate assay	(Rademacher 2006)
460	32	20	5.4	Fluorometric substrate assay	(Rademacher 2006)
250	5	15	96.2962963	ELISA	(Bravo 2015)
450	5	15	81.48148148	ELISA	(Bravo 2015)
550	5	15	48.14814815	ELISA	(Bravo 2015)
700	5	15	40.74074074	ELISA	(Bravo 2015)
800	5	15	37.03703704	ELISA	(Bravo 2015)
900	5	15	37.03703704	ELISA	(Bravo 2015)
**Xanthine oxidase**					
500	30	25	96.08695652	Substrate assay	(Olsen 2004)
500	60	25	91.73913043	Substrate assay	(Olsen 2004)
600	5	25	85.2173913	Substrate assay	(Olsen 2004)
600	10	25	76.08695652	Substrate assay	(Olsen 2004)
600	15	25	66.86956522	Substrate assay	(Olsen 2004)
700	2	25	81.95652174	Substrate assay	(Olsen 2004)
700	4	25	68.04347826	Substrate assay	(Olsen 2004)
700	6	25	60.86956522	Substrate assay	(Olsen 2004)

The lowest pressure treatments that achieved a >5‐log reduction in *L. monocytogenes* were at 200 MPa for 15 min at temperatures of 45 and 55°C (7.64‐ and 5.64‐log reduction, respectively; Simpson and Gilmour 1997). The lowest temperature treatment used for achieving a >5‐log reduction in *L. monocytogenes* was 20°C (with 600 MPa for 15 min), and the lowest time treatment achieving a >5‐log reduction was conducted for 2 min (with 600 MPa at 27°C).

Other articles that investigated pressure treatments of 200 MPa did not achieve 5‐log reductions (Karatzas et al. 2002; Komora et al. 2020, p. 100; Ramos et al. 2015), likely as they did not apply elevated temperatures. At pressures 300–350 MPa, >5‐log reductions were achieved in four articles (Chen and Hoover 2004; Dogan and Erkmen 2004; Huang et al. 2015; Styles et al. 1991), all of which employed extreme time treatments (40–600 min, temperature 10–20°C).

For *L. monocytogenes*, pressures 301–450 MPa were the most investigated and contained the greatest variability in reductions (0.39–8.87 log) between papers. Reasons for variability include exploration of extreme temperature and time treatments, evaluation of different agar types, bacterial incubation times/temperatures and different strains of *L. monocytogenes* (i.e. Scott A, paratuberculosis, enteritidis, typhimurium, 4b). At pressures 301–450 MPa, fourteen articles had at least one treatment combination that achieved >5‐log reductions (Amina et al. 2010; Chen et al. 2006; Chen and Hoover 2003; Chen and Hoover 2004; Dogan and Erkmen 2004; Erkmen and Dogan 2004; Hayman et al. 2007; Huang et al. 2015; Mcclements et al. 2001; Mishra et al. 2013; Patterson et al. 1995; Ramos et al. 2015; Simpson and Gilmour 1997; Wen et al. 2009) and twenty‐two had at least one treatment combination that achieved <5 log reductions (Allison et al. 2018; Amina et al. 2010; Buzrul et al. 2009; Chen et al. 2006; Chen and Hoover 2003; Chen and Hoover 2004; Dogan and Erkmen 2004; Erkmen and Dogan 2004; Hayman et al. 2007, Hayman et al. 2008; Koseki et al. 2008; Liu et al. 2017; Mcclements et al. 2001; Mishra et al. 2013; Misiou et al. 2018; Patterson et al. 1995; Ramos et al. 2015; Shearer et al. 2009; Stratakos et al. 2019; Wen et al. 2009). Specific pressure, temperature and time treatments for all bacteria are reported in Table S4.

At the higher pressures of 451–550 MPa, ten articles had at least one treatment combination that achieved >5‐log reductions (Amina et al. 2010; Chen et al. 2006; Chen and Hoover 2003; Chen and Hoover 2004; Kabir et al. 2021; Koseki et al. 2008; Liu et al. 2017; Mishra et al. 2013; Misiou et al. 2018; Ramos et al. 2015), and seven articles had at least one treatment combination with <5‐log reductions (Amina et al. 2010; Chen and Hoover 2003; Chen and Hoover 2004; Kabir et al. 2021; Mishra et al. 2013; Shearer et al. 2009; Stratakos et al. 2019). The articles that achieved >5‐log reduction applied pressure for 5–15 min at 20–47°C, which are considered moderate temperatures and time treatments compared to temperatures >60°C and times >25 min (Table S4). Based on these observations, it is apparent that pressures of >450 MPa can sufficiently reduce certain strains *L. monocytogenes*.

At pressures >551 MPa, nine articles included at least one treatment combination that achieved >5‐log reductions (Amina et al. 2010; Chen et al. 2006; Chen 2007; Chen and Hoover 2004; Dogan and Erkmen 2004; Erkmen and Dogan 2004; Mishra et al. 2013; Ramos et al. 2015; Stratakos et al. 2019) and six articles included at least one treatment combination that achieved <5‐log reductions (Amina et al. 2010; Chen and Hoover 2004; Dogan and Erkmen 2004; Erkmen and Dogan 2004; Mishra et al. 2013; Stratakos et al. 2019). For the articles that did not achieve 5‐log reductions, the times tested were 0–2 min at 20–30°C (Table S4). This finding shows that pressurization and depressurization alone for a single cycle, even at high pressures, is not enough to inactivate *L. monocytogenes*; longer treatment times (>5 min) are required when treating at high pressures and moderate temperatures (20–30°C).

The inter‐study variation of the effect of these pressure ranges on *L. monocytogenes* log reduction in milk illustrate both the need for standardized approaches to HPP application parameters for validation of specific strain inactivation and the nearly limitless potential to constantly refine operating parameters to maximize the efficacy of HPP. Variation between papers was further shown in Figure 3, and although there is variation in reduction of *L. monocytogenes* at different pressures, the trends are generally consistent between papers, with two exceptions (Shearer et al. 2009; Simpson and Gilmour 1997). The reason that these two articles showed decreasing inactivation of *L. monocytogenes* as pressures increased is unclear. For most papers, increasing pressure was strongly correlated with increasing reductions. The rate at which bacterial reduction increased with increased pressure was amplified in treatments at higher temperatures (as seen by increased linear slope; Huang et al. 2015), showing a synergistic effect between pressure and thermal treatments.

Results show that at treatments of 300–450 MPa, inactivation of *L. monocytogenes* is possible, however minimum time/temperature intensities required must be evaluated based on nutritional outcomes and practicality (i.e. treatments times of 40+ minutes may be impractical).

#### 3.3.2 Salmonella enterica

*S. enterica* had a similar response to HPP as *L. monocytogenes*, but was slightly more pressure‐resistant (Figure 2). Also, on average across studies, a >5‐log reduction of *S. enterica* required pressure treatments >500 MPa (Figure 2).

For *S. enterica*, the minimum pressure treatment that achieved a >5‐log reduction was 300 MPa (at 20°C for 30 min; Foster et al. 2016). The minimum time treatment to achieve >5‐log reduction was 4 min (at 600 MPa at 20°C; Chen 2007). The minimum temperature treatment investigated to achieve >5 log reduction was 3°C (at 600 MPa for 5 min; Stratakos et al. 2019). These treatments illustrate how each major operating parameter individually may be conducted at lower intensities, but if the remaining parameters are increased to compensate, a 5‐log reduction is still possible. Notably, increasing temperatures >40°C began to negate the non‐thermal benefits of HPP.

Of the papers that evaluated *S*. *enterica*, no literature reported a >5‐log reduction at pressures <300 MPa. Three articles (Chen et al. 2006; Liu et al. 2017; Nakimbugwe et al. 2006) reported reductions of <5‐log at treatments <251 MPa.

From 300–400 MPa, 3 articles (Erkmen and Dogan 2004; Foster et al. 2016; Liu et al. 2017) reported a >5‐log reduction at times between 30–45 min (20°C for all; Table S4) and 5 articles (Chen et al. 2006; Erkmen and Dogan 2004; Guan et al. 2005; Liu et al. 2017; Stratakos et al. 2019) reported a reduction of <5‐log at times 10–120 min (20°C for all; Table S4). It is unknown why time treatments of 120 min were unable to achieve >5 log reductions at these pressures.

From 401–600 MPa, 4 articles (Chen et al. 2006; Chen 2007; Guan et al. 2005; Stratakos et al. 2019) reported a >5‐log reduction in *S. enterica*, but each of these papers reported on specific conditions within this pressure range that produced both greater than and less than 5‐log reductions. Less than 5‐log reductions were reported at 401–600 MPa, included time treatments of 5–120 min and temperatures 18–50°C (or temperature not reported). Parameters that led to >5‐log reductions at this pressure range included 1–50 min and 18–50°C. The overlap in temperature and time parameters shown may be because the treatments that did not achieve >5‐log reductions included only an increase in temperature or time treatment. However, the treatments that did achieve reductions >5‐log applied elevated time and temperature treatments. Only a single article by Chen et al. (2006) investigated pressures above 600 MPa (650 and 690 MPa), and all treatments investigated achieved >5 log reductions at 10 min and 20°C.

#### 3.3.3 Staphylococcus aureus

The minimum pressure, temperature, and time treatment for achieving a >5‐log reduction in *S. aureus* was 200 MPa, 20°C, and 4 min respectively. Each of these parameters were able achieve >5‐log reduction by increasing the remaining two operating parameters to extreme levels.

For *S. aureus*, the literature reviewed showed that from <300 MPa, 2 articles (Gao et al. 2005; Patterson and Kilpatrick 1998) included at least one treatment condition that achieved a >5‐log reduction, and 6 articles (Chen et al. 2006; Erkmen and Karataş 1997; Gao et al. 2005; Liu et al. 2017; Patterson and Kilpatrick 1998; Ramos et al. 2015) included a treatment condition that resulted in a <5‐log reduction. As expected, the articles with treatments that achieved >5‐log reductions were conducted at an elevated temperature (both at 60°C). HPP parameters employing such high temperatures would no longer be considered “non‐thermal” and would likely induce similar protein and sensory degradations as thermal pasteurization.

**FIGURE 4:**
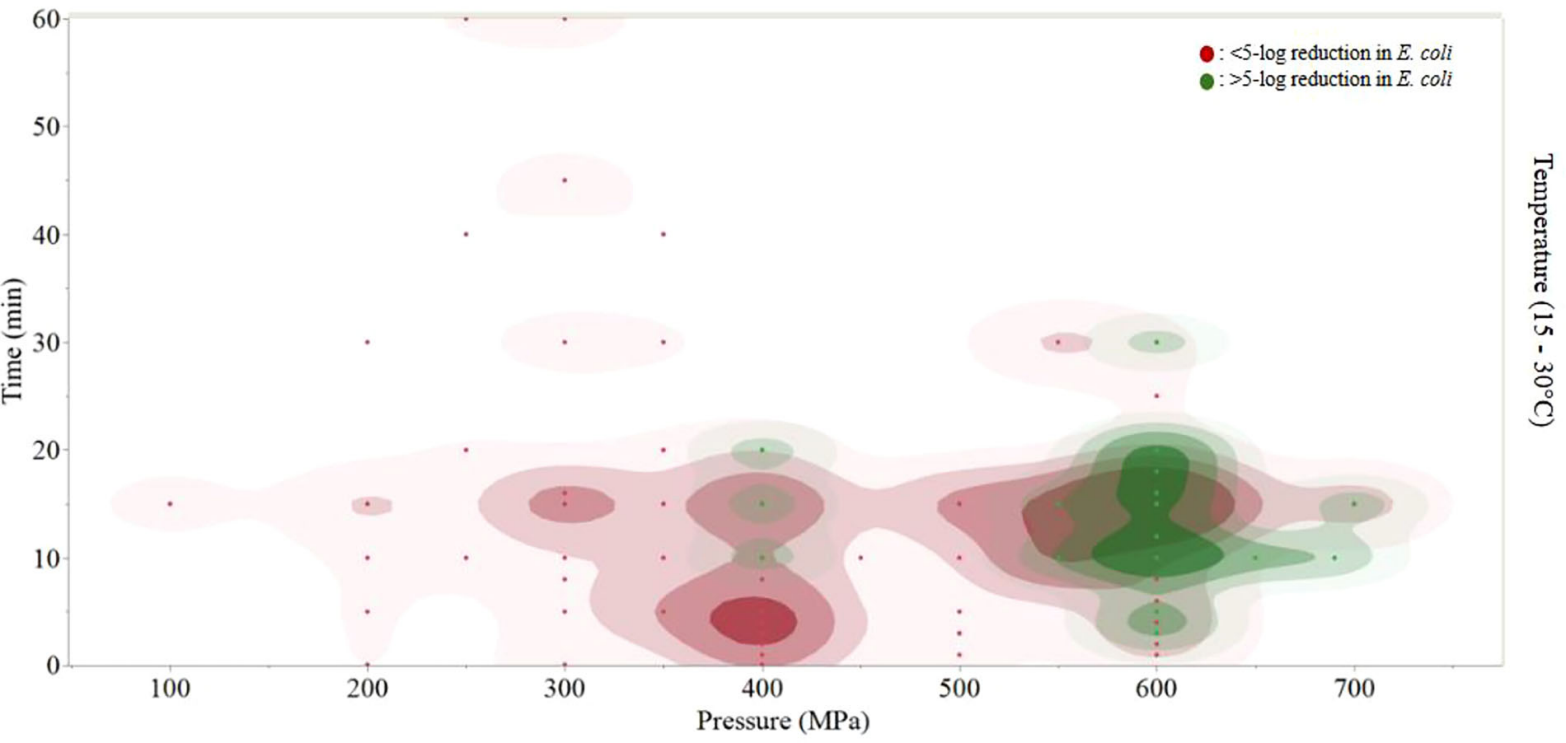
Contour plot of treatments that achieved >5 log reductions (green) and <5 log reductions (red) for *E. coli* at temperatures of 15—30°C.

At 300–400 MPa, results in *S. aureus* inactivation varied. Four articles (Alpas and Bozoglu 2000; Erkmen and Karataş 1997; Gao et al. 2005; Patterson et al. 1995) reported a treatment combination that achieved >5‐log reduction, and 7 articles (Chen et al. 2006; Erkmen and Karataş 1997; Gao et al. 2005; Liu et al. 2017; Patterson and Kilpatrick 1998; Simpson and Gilmour 1997) reported a <5‐log reduction of pathogens. Note that three of the articles that achieved >5‐log reductions also presented lower intensity parameter combinations within this pressure range that resulted in <5‐log reductions. Between different papers, treatments that were more extreme in time, temperature and pressure (example: 400 MPa, 50°C, 15 min, (Patterson and Kilpatrick 1998)) achieved smaller reductions than papers that performed less intense treatments (example: 300 MPa, 30°C, 15 min; Gao et al. 2005) even when milk fat and pressurization rate remained the same (Figure 4).

Inconsistency in reductions of *S. aureus* continued at pressures from 401–600 MPa. Seven articles (Chen et al. 2006; Chen 2007; Guan et al. 2006; Kabir et al. 2021; Patterson et al. 1995; Patterson and Kilpatrick 1998; Ramos et al. 2015; Tabla et al. 2012) included at least one treatment combination resulting in a >5‐log reduction, and eight articles (Chen et al. 2006; Chen 2007; Guan et al. 2006; Kabir et al. 2021; Patterson et al. 1995; Patterson and Kilpatrick 1998; Ramos et al. 2015; Tabla et al. 2012) reported a treatment combination with <5‐log reduction. Temperatures tested ranged from 20–22°C, and times ranged from 2–50 min (treatments shown Table S4), with time ranges from 2–22 min not achieving reductions >5‐log and time treatments between 8–50 min achieving reductions >5‐log.

For elevated pressures of *S. aureus* (>600 MPa), three articles (Chen et al. 2006; Patterson and Kilpatrick 1998; Syed et al. 2014) included at least one treatment combination that achieved >5‐log reduction, and 1 article (Syed et al. 2014) reported at least one treatment combination with a <5‐log reduction. The article reporting <5‐log reduction applied a minimal time and temperature (700 MPa, 4°C, 5 min). Moderate temperatures (20–25°C) and times (10–15 min) were conducted for all treatments that achieved >5‐log reductions.

#### 3.3.4 Escherichia coli

Averaged between studies, no treatments of *E. coli* resulted in a >5‐log reduction, but this was likely due to inter‐strain variation. There were pockets of high inactivation overlayed with high activity of *E. coli* at 600 MPa (Figure 4) which is likely the cause of the averaged inactivation being <5‐log CFU/mL. Likely, several studies evaluated baro‐resistant and baro‐sensitive strains (García Graells et al. 1999).

The minimum pressure required to achieve >5‐log reduction in *E. coli* was 200 MPa (at 50°C and 15 min). The minimum temperature required to achieve >5‐log reduction was 20°C (at 400 MPa for 20 min) and the minimum time required was 5 min (at 400 MPa at an unreported temperature). Each of these parameters were able achieve >5‐log reduction by increasing the remaining two operating parameters to extreme levels.

For *E. coli*, from <300 MPa, 3 articles (Bulut 2014; Park et al. 2009; Patterson and Kilpatrick 1998) reported at least one treatment combination with >5‐log reduction, whereas 11 articles (Chen et al. 2006; Foster et al. 2016; García Graells et al. 1999; García‐Graells et al. 2000; Pandey et al. 2003; Bulut 2014.; Li et al. 2020; Liu et al. 2017; Park et al. 2009; Patterson and Kilpatrick 1998; Ramaswamy et al. 2009) reported a treatment combination with <5‐log reduction. The article parameters that resulted in >5 log reductions at these pressures used temperatures between 50–55°C (Table S4). The >5‐log reductions at low pressures require intense temperature.

At 301–450 MPa, 3 articles (Foster et al. 2016; Liu et al. 2017; Patterson and Kilpatrick 1998) reported a at least one treatment combination with >5‐log reduction, and 10 articles (Buzrul et al. 2009; Chen et al. 2006; García Graells et al. 1999; García‐Graells et al. 2000; Pandey et al. 2003; Li et al. 2020; Nakimbugwe et al. 2006; Patterson and Kilpatrick 1998; Ramaswamy et al. 2009; Stratakos et al. 2019) reported a treatment combination with <5‐log reduction. For papers that achieved >5‐log reduction, extreme temperature treatments were conducted (50°C for all articles; Table S4).

From 451–600 MPa, 8 articles (Chen et al. 2006; Chen 2007; García Graells et al. 1999; García‐Graells et al. 2000; Guan et al. 2006; Liu et al. 2017; Patterson and Kilpatrick 1998; Stratakos et al. 2019) reported parameter combinations that achieved a >5‐log reduction, whereas 9 articles (Chen et al. 2006; Chen 2007; Erkmen and Karataş 1997; García‐Graells et al. 2000; Guan et al. 2006; Patterson et al. 1995; Patterson and Kilpatrick 1998; Stratakos et al. 2019; Syed et al. 2013) reported combinations that resulted in <5‐log reductions (Table S4). At 600 MPa, at similar treatments (3–20 min, 15–30°C), there are differences in log‐reductions of *E. coli* (Chen et al. 2006; Chen 2007; García Graells et al. 1999; García‐Graells et al. 2000; Guan et al. 2006; Patterson et al. 1995; Patterson and Kilpatrick 1998; Stratakos et al. 2019). Figure 4 shows that at 600 MPa, there are treatments conducted at extremely similar parameters (pressure, temperature and time) that achieved reductions in *E. coli* both greater and less than 5‐log, which illustrates the inconsistency throughout the data in the efficacy of HPP for reductions of bacteria.

Finally, at >601 MPa, 2 articles (Chen et al. 2006; Patterson and Kilpatrick 1998) reported a >5‐log reduction, and 2 articles (García Graells et al. 1999; Patterson and Kilpatrick 1998) reported a <5‐log reduction. Parameter combinations that did achieve >5‐log reductions at pressures >600 MPa were at 650–700 MPa, 22–40°C, and 10–15 min. One article that did not achieve a >5‐log reduction at 700 MPa, 15 min, 20°C reported that this was due to the use of a pressure‐resistant strain of *E. coli* (LM1010) (García Graells et al. 1999).

Differences in effect of HPP on *E. coli* due to milk fat content (3.6, 1.55 and 0.05%) was investigated by Garcia et al. (1999). They observed that milk fat had a protective effect for *E. coli*. Treating whole fat milk (3.6% milk fat) at 600 MPa for 15 min at 20°C achieved a 1.6‐log reduction in *E. coli*, whereas treating half‐whole (1.55% milk fat) and skim milk (0.05% milk fat) at the same parameters achieved 2.3‐ and 3.0‐log reductions, respectively. Gervilla et al. (2000) suggests that fat acts as a buffer between bacteria and environmental substances, reducing transfer of inter and intracellular substances compared to skim milk samples. However, there is some contradictory evidence suggesting that milk fat has no protective role, but that it could be another factor within in the milk matrix (Garcia et al. 1999; Jiao et al. 2016; Olsen et al. 2004; Ramaswamy et al. 2009). Further research is needed to determine whether milk fat is a contributor or whether other factors are responsible for the protective effects.

Overall, *E. coli* was the least predictable and most pressure‐resistant bacterial species when observing the effects of pressure on microbial safety (Figure 4). This strain‐to‐strain variation should be further characterized in future research. Additionally, food processing conditions should be validated using the pathogen of concern for that food matrix that demonstrates the highest degree of resistance to the chosen process. The finding that *E. coli* was the most resistant vegetative bacteria to pressure treatments in past studies suggests that future studies should focus on optimizing HPP parameters for consistent reductions in *E. coli* in milk.

### 3.4 Impact of HPP Cycling on Bacterial Reduction

Pressure cycling is a pressure treatment that consists of the repeated application and release of pressure in a controlled manner. Three papers investigated the effects of pressure cycling on *E. coli* and/or *L. innocua* (Buzrul et al. 2009; García Graells et al. 1999; Li et al. 2020). Buzrul et al. and Li et al. investigated the effect of cycling at 400 MPa on *E. coli*. Both authors found that increasing from single‐ to dual‐cycles increased the reductions in bacteria by 1 to 1.5 log at the same treatment times. Buzrul et al. additionally investigated 1, 2, 4, 5, and 10 cycles for times of 20, 10, 5, 4, and 2 min per cycle (total pressure holding time 20 min for all treatments at 400 MPa) and observed increases in reductions from 1 to 4.3 log in *E. coli* from 1 to 10 cycles, with decreasing rates of additional inactivation as cycles increased, illustrating diminishing returns of increasing cycled treatments. The diminishing rates of effectiveness was proposed to be because of the variation in pressure resistance in the cellular population (even within the same strain) causing inactivation of pressure‐sensitive cells but leaving pressure‐resistant cells unaffected. No treatments tested by Buzrul et al. or Li et al. achieved >5 log reductions, showing that at moderate temperatures (20°C) and total times 20 min, pressures >400 MPa are required for inactivation of *E. coli* and *L. innocua*, even with cycled treatments.

One paper investigated the effect of HPP cycling on different strains of *E. coli*. Garcia et al. investigated 1, 2, and 3 cycles at 10 min per cycle at 550 MPa at 20°C for 3 pressure‐resistant strains of *E. coli* (LMM1010, LMM1020 and LMM 1030) and a parent strain of *E. coli* (MG 1655). Comparisons were made to a single‐cycle 30 min treatment (550 MPa, 20°C). At 30 min, there were differences of 3.9‐log reductions between 1 and 3 cycles with final reductions of 6‐log for the three 10‐min cycle treatment for the parent strain (MG 1655). The parent strain was the only bacteria tested to achieve >5 log reduction, and this only occurred at the highest cycled treatment. The most pressure‐resistant strain (LMM1030) had no reductions at 30 min, 1 cycle, but 0.8‐log reduction for the three 10‐min cycle treatment. These drastic differences in pressure sensitivity between strains illustrates the need for reporting of subspecies used in bacterial analysis for HPP and the need to test against the most pressure‐resistant pathogenic strains found in milk.

### 3.5 HPP Using Antimicrobial Additives

Though the current use of additives (except vitamins, vitamin carriers and milk flavoring ingredients) to milk is not legal in the U.S. food system (Milk 1998), there is a growing body of literature on antimicrobial additives (Ludikhuyze et al. 2001). The addition of antimicrobial proteins, peptides, phages and other molecules in an HPP system has been hypothesized to increase effectiveness in bacterial reductions due to the additional stressor applied (Oliveira et al. 2015). The use of all additives investigated (with the exception of nisin) are accepted for use in Canada, United States, Australia and New Zealand, but not the European Union, by their governing bodies (Quinto et al. 2019). Currently, antimicrobial additives are allowed in select food products such as cheeses, wines, seafood, fruits and vegetables to assist with processing (Program 2024b) in these countries. In the literature collected in this review, the antimicrobials tested were lambda lysozyme (LaL), endolysin PlyP825, epsilon poly‐l‐lysine (ε‐PL), potassium sorbate (C_6_H_7_O_2_K), pediocin PA‐1, P100 bacteriophage, LPO, H_2_O_2_, nisin, bactericidal/permeability‐increasing antimicrobial (BP_1_) and phages philPLA35 and philPLA88. No authors investigated multiple of these antimicrobials within the same manuscript, so comparison of the same antimicrobial across studies was impossible. Pathogens investigated include *E. coli* (*n* = 5), *L. monocytogenes* (*n* = 5), *S. aureus* (*n* = 3), *S. enterica* (*n* = 2), *L. innocua* (*n* = 1), *Y. enterocolitica* (*n* = 1) and *S. flexneri* (*n* = 1). In all cases, HPP with additional antimicrobials increased bacterial reductions compared to HPP treatments without antimicrobials added (0.8 to 3 log). The finding also indicates that these antimicrobials are resistant to pressure inactivation and function during pressurization (300–550 MPa) as they were compared to non‐pressurized controls with antimicrobials added. Treatments that achieved >5‐log reductions in *E. coli* were found by Garcia et al. at 550 MPa, 20°C, 15 min with 20 µg/mL of LPO (García‐Graells et al. 2000) and Alpas et al. at 345 MPa, 50°C, 5 min with nisin and pediocin (BP_1_) in unreported concentrations (Alpas and Bozoglu 2002b). Notably, Alpas et al. also achieved a >5 log reduction without additional antimicrobials (exact values for reductions not given), so it is not possible to examine the isolated effect of BP_1_ on milk in a HPP system from this article. The use of antimicrobial additives remains underexplored, with the potential to benefit the dairy industry by opening up the possibility of an optimized treatment that includes combinations of pressure, time and temperature exposure with antimicrobial additives to allow sufficient microbial reductions at minimal processing conditions.

### 3.6 Additional Factors Affecting Microbial Reduction

Some causes for variation in pressure resistance between bacterial species can be attributed to gram‐positive bacteria typically being more pressure‐resistant than gram‐negative bacteria due to increased levels of peptidoglycan in the cell walls (Wuytack et al. 2002), spherical (cocci) shaped bacteria being more resistant than rod (bacilli) or spiral (spirilla) shaped bacteria due to physical compression dynamics (Young 2006), differences in membrane fluidity (Pucciarelli et al. 2007) or specialized responses to external stressors. However, the interconnectedness of these factors makes it difficult to distinguish their individual effects on bacterial reductions, and some may have a greater effect than others. Further research is required to clarify the impacts of these factors individually and in aggregate.

Bacterial cocktails showed greater resistance to HPP than single‐strain inoculations due to the inter‐strain diversity in genetic expression with some strains better adapted for pressure resistance (Kabir et al. 2021; López‐Pedemonte et al. 2006; De Lamo‐Castellvì et al. 2005). This greater resistance is due to HPP selecting for the most pressure‐resistant strain in the cocktail (García Graells et al. 1999; Kabir et al. 2021). Major HPP operating parameters that affected results included pressure, temperature and time. Mixed methods analysis revealed that pressure was the strongest contributor to logarithmic reductions in *L. monocytogenes*, *E. coli*, and *S. aureus* (*p‐*value <0.05; Table 1) among the parameters tested in literature. Temperature significantly contributed to reductions in bacteria tested (*p‐*value <0.05). Time significantly contributed to rates of denaturation as pressure increased for all bacteria except for *S. aureus* (*p‐*value = 0.06).

Minor operating parameters across literature include pressurization and depressurization rates, packaging type and operating equipment. Due to inconsistency between reports in the literature (if they were included), only narrative analysis was conducted on all minor parameters. Effect of pressurization and depressurization rates were examined in spores by Syed et al. (Syed et al. 2012). At pressures of 600 MPa, temperatures of 70°C and dwell time of 3 min, it was observed that slower pressurization and depressurization rates caused greater reductions in *B. subtilis* spores than faster pressurization and depressurization rates (5 MPa/s to 33 MPa/s pressurization and 5 to 70 MPa/s depressurization). While it is currently unclear what mechanism causes the increased inactivation (1.4 log) in spores during slower pressurization and depressurization rate treatments, it's possible that slower pressurization would increase the pressure and thermal dosage and correspondingly increase lethality (Janahar et al. 2023).

### 3.7 Effect of Pressure on Protein Structural Retention

**FIGURE 5:**
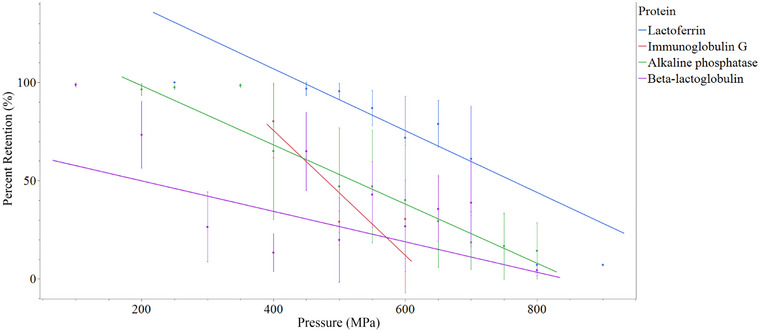
Average reductions (points) with ±SD in IgG, LF, ALP and β‐Lg across collected literature. Lines show linear average trends for LF (blue), IgG (red), ALP (green), β‐Lg (purple).

Among the top 4 most frequently reported proteins from literature collected (*n* = 20), LF was the most resistant to pressure, followed by ALP, IgG, and β‐Lg (Figure 5). An average protein denaturation of 50% occurred for β‐Lg between 200–300 MPa, compared to at 400, 600, and 800 MPa for IgG, ALP, and LF, respectively.

Ramos et al. investigated the effect of HPP on bacteria and multiple proteins (Ramos et al. 2015). They found that pressures of 600 MPa for 15 min at 20°C was the only treatment successful at reducing *L. monocytogenes*, *P. aeruginosa*, and *S. aureus* by more than 5‐log in mature skim milk. At this treatment, percent retentions in IgG, LF, LPO, α‐LA, and β‐Lg were seen to be 57.02%, 39.0%, 100.0%, 94.0%, and 20.1%, respectively. This article illustrates the potential for HPP to preserve activity of some proteins (IgG, LPO, α‐La), but not others (β‐Lg), at pressures capable of generating microbial safety.

Specific protein retentions are discussed in context of treatments that achieved >5 log reductions in *L. monocytogenes*, *S. enterica*, and *S. aureus*. No pressure treatments achieved average reductions >5 log for *E. coli* so only these three species were considered. All pressure treatments equal to or above 600 MPa were considered as average reductions in bacterial load were greater than 5‐log for *S. aureus*, *S. enterica*, and *L. monocytogenes* at these pressures.

#### 3.7.1 Beta‐Lactoglobulin

Although β‐Lg was the most degraded across pressure (Lopez‐Fandiño et al. 1996), the rate of denaturation of β‐Lg as pressure increased was lower than for ALP, LF, or IgG (Figure 5). This finding shows that after initial denaturation, β‐Lg was least impacted by increasing pressures.

Although β‐Lg was also the most investigated protein (*n* = 8 articles), only 2 articles investigated pressures above 600 MPa (Hinrichs and Rademacher 2005; Mazri et al. 2012b). Mazri et al. conducted HPP treatments at 650 MPa for 2–15 min at 20°C, observing β‐Lg retentions of 65.31 to 17.62% as time treatments increased. Hinrichs et al. investigated treatments up to 800 MPa, where β‐Lg was massively reduced, observing retentions of 4.8–1.9% as time treatments increased from 12–32 min. These treatments illustrate the deleterious effect of very high pressures on protein retention.

Ramos et al. investigated the effect of HPP on bacteria and proteins (2015). They found that HPP at 600 MPa for 15 min at 20°C was the only treatment successful at reducing *L. monocytogenes*, *P. aeruginosa*, and *S. aureus* by more than 5‐log in skim milk. At this treatment, percent retention of β‐Lg was 20.1%. As the extended treatment times generally result in reduced native β‐Lg, further refinements of HPP parameters, perhaps for high pressure and short time, may better preserve the protein structure.

#### 3.7.2 Alkaline Phosphatase

For ALP, three articles investigated pressure treatments >600 MPa (Martínez‐Monteagudo et al. 2014; Koncz et al. 2007; Rademacher and Hinrichs 2006). Treatments investigated included pressures 620–800 MPa, temperatures 10–20°C and times 2–128 min. Denaturation of ALP ranged from 42% to >99.2% based on intensity of treatment (treatment with longest time and highest temperature causing the greatest reduction).

To test the pressure‐resistance of ALP compared to pathogens, kinetic inactivation studies were conducted by Rademacher and Hinrichs (2006) at pressures from 400–800 MPa, temperatures 5–40°C and times 2–128 min. In this study, Rademacher et al. observed that ALP denaturation was accelerated by higher temperatures, and that there were synergistic effects between time and temperature on native ALP reduction (Rademacher and Hinrichs 2006; Rankin et al. 2010; Mussa and Ramaswamy 1997).

There is some evidence to suggest a functional benefit from consuming ALP from bovine milk for adults. ALP is reported to have prebiotic, probiotic and immuno‐protective effects (Wu et al. 2022). Thus, further optimization of native ALP retention could result in additional functional benefits to consuming safely pasteurized milk.

#### 3.7.3 Lactoferrin

Compared to other proteins investigated, LF had the greatest resistance to HPP treatments across all pressures. At pressure treatments above 600 MPa, two articles investigated LF. Treatments include pressures 650–900 MPa, temperatures 15–20°C and times 4–15 min. Percent retentions of LF from the treatments >600 MPa were between 94.44% (at 650 MPa, 20°C for 4 min) and 7.14% (at 900 MPa, 15°C for 5 min). The high retention of LF should be verified by additional studies conducting treatments at 650 MPa. The implications for LF preservation also extend to preservation of milk bioactivity, including possible antimicrobial and immunomodulatory effects (Haug et al. 2007). Future studies should consider directly comparing HPP and thermally pasteurized milk functionality.

#### 3.7.4 Immunoglobulin G

For IgG, there were no studies that investigated pressures above 600 MPa. From studies collected, pressures of 500 or 600 MPa retentions of IgG ranged from 57.02% to 4% (10–15 min and 20–30°C). Ramos et al. found that pressures of 600 MPa for 15 min at 20°C was successful in reducing vegetative bacteria more than 5‐log, and at this treatment percent retention of IgG was 57.02% (2015). Bovine IgG has the ability to reduce instances of inflammation and infection by binding to human pathogens and some allergens (Ulfman et al. 2018). Further studies should be conducted observing the effect of HPP on IgG, including refining parameters for improved preservation.

### 3.8 Additional Factors Affecting Native Protein Retention

For protein analysis, pressure was the most significant contributor to denaturation (*F* = 52.69) followed by temperature (*F* = 13.9) (Garcìa‐Risco et al. 2000; López‐Fandiño and Olano 1998). Time did not significantly contribute to reductions in native proteins based on treatments collected across literature (*p* = 0.06).

Protein pressure resistance is affected by multiple factors. Researchers have hypothesized that dimeric proteins such as β‐Lg and ALP are more pressure‐sensitive, due to their increased size and fragile bonds that link monomeric isomers together, compared to naturally monomeric proteins such as LF or IgG (Cioni and Strambini 1994). This pattern of sensitivity has been validated for immunoglobulins in milk: IgM (pentameric) is more pressure‐sensitive than IgA (dimeric), which is more pressure‐sensitive than IgG (monomeric; Mazri et al. 2012).

In addition to other causes of variance discussed, differences in protein assessment (e.g. HPLC, ELISA, radial diffusion) may cause some observed variance in the effects of specific HPP parameters on protein retention in bovine milk. For example, two studies (Hinrichs and Rademacher 2005; Mazri et al. 2012b) performed identical HPP treatments (500 MPa, 15 min, 20°C) and achieved different results in percent protein structural retention. For β‐Lg, 41.19% retention was measured by ELISA and 28.47% was measured by HPLC. Additionally, Mazri et al. (2012). observed a 19.51% structural retention of β‐Lg via ELISA, and Ramos et al. (2015) observed 20.05% structural retention using radial immunodiffusion after both applying 600 MPa of pressure for 15 min at 20°C. Future research is required to investigate the impact of assessment type on protein retention determinations after HPP treatment.

### 3.9 Strengths and Limitations

Variation in outcomes from literature collected was found throughout the review. It is crucial for continued HPP research to report all relevant operating parameters and identify the most pressure‐resistant bacterial strains that are of concern in the milk matrix.

Discovery of the minimum intensity for each operating parameter needed to inactivate the most pressure‐resistant bacteria of concern reported herein will provide evidence to regulatory bodies such as the FDA for the possible use of HPP in milk. Additionally, all nutritional or sensory outcomes should be paired with bacterial investigation to confirm that adequate bacterial reductions are achieved at included treatments (Lim et al. 2023; Linton et al. 2008; Yu et al. 2022).

Antimicrobial additives and cycling are more potential ways to further optimize HPP for bacterial reduction and protein retention. If lower intensities of other operating parameters (pressure, temperature, time) can be used in addition to cycling or antimicrobial additives for achieving >5‐log reductions, there may be a benefit to the retention of bioactive proteins and other nutritional components.

A limitation of this review is that there is no direct comparison provided of HPP with other types of pasteurization (HTST, LTLT etc.). Other types of pasteurization may better preserve native structure of baro‐sensitive bioactive proteins while HPP may better preserve heat‐labile bioactive proteins, and the optimal preservation of milk's healing components may be a combination of both (Evelyn et al. 2017; Donaghy et al. 2007). Future research should consider these side‐by‐side comparisons of alternative pasteurization methods to better understand how to optimally preserve bioactivity.

## 4 Conclusion

Although HPP in milk has been studied in milk for more than 75 years, there is still much to discover about what the optimal treatment parameters are for bovine milk. Research indicated that higher pressures (>600 MPa) were required to achieve microbially safe products (>5‐log reductions). At extreme temperatures (>60°C) or extreme times (>40 min), >5‐log reductions were achieved, regardless of other treatment parameters. Although extreme pressure, temperature or time treatments were effective at reducing bacteria, they also showed considerable reductions in proteins identified.

This systematic review highlights gaps in consistency and reporting practices that hinder direct comparison across studies. Moving forward, future studies should continue to focus on further optimization of HPP parameters (e.g. pressure, time, temperature, pressurization/depressurization rates) for bacterial reduction and native protein preservation in bovine milk. Studies should employ bacterial strains known to be pressure resistant (e.g. *E. coli*) and relevant to bovine milk. Such efforts are needed to determine whether HPP can improve bioactive protein preservation while maintaining microbial safety compared with current thermal treatments. Once optimized, studies will need to be conducted to determine how HPP‐treated bovine milk impacts the health of consumers compared with standard thermal pasteurization.

## Author Contributions

**Rudy Sykora**: methodology, validation, formal analysis, writing – review and editing, writing – original draft, conceptualization, project administration, investigation. **Caleb Mark**: investigation, methodology, validation, data curation, formal analysis. **Marie Ryan**: writing – review and editing, project administration, resources. **Bishal Barman**: writing – review and editing, investigation, formal analysis. **Michael Pitino**: conceptualization, methodology, writing – review and editing, project administration. **David C. Dallas**: conceptualization, funding acquisition, writing – review and editing, project administration.

## Funding

This work was supported by BUILD Dairy and Oregon Dairy Council.

## Conflicts of Interest

The authors declare no conflicts of interest.

## Supporting information



crf370324‐sup‐0001‐SuppMat.docx
