# Supplementary material for: Effect of High‐Pressure Processing Operating Parameters on Microbial Inactivation and Bioactive Protein Preservation in Bovine Milk: A Systematic Review
Source: Compr Rev Food Sci Food Saf. 2025 Nov 14;25(1):e70324. doi: 10.1111/1541-4337.70324 (PMC12616593; doi:10.1111/1541-4337.70324)
Supplement: Supplementary file 1 — crf370324‐sup‐0001‐SuppMat.docx [file CRF3-25-e70324-s001.docx]

**Supplementary Table 1:** Search strings and article results for indexed databases

| **Web of Science search string:** | | |
| --- | --- | --- |
| Line: | Search String: | Results: |
| Line 1: | ((ALL=(milk) OR ALL=(dairy)) OR ALL=(colostrum)) | 458,624 |
| Line 2: | (((((((ALL=(high pressure processing)) OR ALL=(HPP)) OR ALL=(high hydrostatic pressure)) OR ALL=(HHP)) OR ALL=(ultra high pressure)) OR ALL=(UHP)) OR ALL=(UV)) OR ALL=(Ultraviolet) | 1,052,251 |
| Line 3: | 1 AND 2 | 6,981 |
| **Search executed Feb 15 2024**  **WOS 1909 to 2024**   **(7 .ris files, 1000 records each)** | | |
| **SCOPUS search string:** | | |
| Line: | Search String: | Results: |
| Line 1: | (TITLE-ABS-KEY ("milk" OR "dairy" OR "colostrum")) | 409,171 |
| Line 2: | (TITLE-ABS-KEY ("high pressure processing" OR "HPP" OR "high hydrostatic pressure" OR "HHP" OR "ultra high pressure" OR "UHP" OR "UV" OR "Ultraviolet")) | 1,032,475 |
| Line 3: | 1 AND 2 | 4898 |
| **Search executed Feb 15 2024**  **Scopus 1926 to 2024**   **Yrs: (1) 2024-2018; (2) 2017-2005; (3) 2004-1926** | | |
| **MEDLINE search string:** | | |
| Line: | Search String: | Results: |
| Line 1: | (milk or dairy or colostrum).mp | 214331 |
| Line 2: | (high pressure processing or HPP or high hydrostatic pressure or HHP or ultra high pressure or UHP or UV or ultraviolet).mp | 330128 |
| Line 3: | 1 AND 2 | 2122 |
| **Search executed Feb 15 2024**  **Ovid MEDLINE (R) ALL 1946 to February Week 2 2024**  **(1) 1-2000; (2) 2000-2120** | | |
| **EMBASE search string:** | | |
| Line: | Search String: | Results: |
| Line 1: | (milk or dairy or colostrum).mp | 277132 |
| Line 2: | (high pressure processing or HPP or high hydrostatic pressure or HHP or ultra high pressure or UHP or UV or ultraviolet).mp | 428114 |
| Line 3: | 1 AND 2 | 3197 |
| **Search executed Feb 15 2024**  **Embase Classic + Embase 1946 to February Week 2 2024**  **(1) 1-2000; (2) 2001-3197** | | |

**Supplementary Table 2:** Eligibility criteria for PRISMA flow diagram based on PEO question structure framework.

|  | **Include** | **Exclude** |
| --- | --- | --- |
| **Population** | 1) Must include bovine milk or bovine colostrum (skim and reduced milk fat will be considered bovine milk/colostrum) | 1) Exclude articles that only analyze suspended bacteria or proteins in "milk buffer" (eg, non-native bovine milk) solutions.  2) Exclude articles that only look non-fat related fractionations (WPC, WPI, Serum, etc.)  3) Exclude articles that only analyze fortified or enriched milk samples. |
| **Intervention/exposure** | 1) Must include HPP or UV-C processing (if articles apply additional processing such as UHT prior  2) Articles can include application of additional processing (UHT, homogenization, etc.) before HPP/UV-C application | 1) Exclude articles that only assess high pressure homogenization (pressures up to 400 MPa in homogenizer equipment) as treatment |
| **Outcome** | 1) Articles must analyze protein retention, concentration, or activity and/or pathogen reduction  2) Articles must include outcome analysis before and after treatments and before digestion | 1) Exclude articles that only measure outcomes secondary to nutrient/bioactive component concentration (e,g,. binding activity, oxidation capacity, digestibility, etc.)  2) Exclude articles that only measure vitamins, lipids, carbohydrates  3) Exclude articles that only assess non-pathogenic bacteria (bifidobacterium, lactic acid bacteria (LAB)) |

**Supplementary Table 3:** Rotated column key for raw data table collecting qualitative and quantitave article information

| **Column number:** | **Heading title:** | **Definition** of column | **Selection options (when applicable)** |
| --- | --- | --- | --- |
| 1 | Code | The unique, numerical identifier for each article. These may found under "Article coding" |  |
| 2 | Author | The first author listed on the article |  |
| 3 | Year | The year the article was published by the journal |  |
| 4 | Country | The country where the majority of the authors are located or where the university is located |  |
| 5 | Purpose | The objectives of the research, usually listed near the end of the background/introduction section. This can be copied and quoted, or can be written as a summary |  |
| 6 | Milk_type | The type of bovine milk used in the study. This is determined by the time collected. | Colostrum - produced during the first 1-4 days after birth  Transitional/premature - produced between 4-14 days after birth  Mature - produced following 14+ days post-partum |
| 7 | Milk_source | Whether or not the milk was collected fresh or collected from store bought milk |  |
| 8 | Pasteurization | If the milk was treated prior to HPP/UV-C treatment to eliminate all potential microbes (usually only for store bought milk) | none - no heat treatment  HTST - roughly 72˚C for 15s   UHT - 130-150 °C for 0.5-1.0s  other - other time/temperature combinations that are not included under these definitions |
| 9 | Fat | Milk-fat content description | Whole, skim, semi-skim |
| 10 | Processing | Qualitative summary of freezing and transport conditions of milk |  |
| 11 | n | Number of samples treated by either HPP or UV-C |  |
| 12 | pre-processing | description of pre-processing metods |  |
| 13 | Equipment | Brand and model of equipment used |  |
| 14 | Packaging | Packaging type or shape | Bag - plastic HPP bags  Bottle - plastic HPP bottles |
| 15 | Material | material used for packaging (example: polyethylene) |  |
| 16 | Volume | Value for sample volume |  |
| 17 | Transmission fluid | Fluid used in HPP chamber to pressurize |  |
| 18 | Unit for pressure | Examples: Mpa, atmosphere |  |
| 19 | Pressure_C | Holding pressure for cycles |  |
| 20 | Time | total time held at pressure |  |
| 21 | number_of_cycles | Number of cycle treatments |  |
| 22 | Pressuriaztion_time | Total time (s) to reach max pressure |  |
| 23 | Pressurization_mag | MPa/s if applicable |  |
| 24 | Pressurization units | Units reported for pressurization |  |
| 25 | Depressurization time | Time to fully depressurize |  |
| 26 | Magnitude of depressurizaiton rate | Rate of depressurization |  |
| 27 | Unit of depressurization rate | Units reported for depressurization |  |
| 28 | Temp | Vessel temperature (C) |  |
| 29 | Functional_additives | Are functionall additives present? | Yes/no |
| 30 | storage summary | Summary of post-processing storage before analytical treatment |  |
| 31 | Bacteria? | Analyte is bacteria? | Yes/no |
| 32 | Protein | Name of protein |  |
| 33 | Activity | Type of analytical method |  |
| 34 | Reported stat | How was data reported? | Mean, median, etc. |
| 35 | Magnitude of measurement of milk component/function (pre-processing) | Pre-processing value |  |
| 36 | Type of variation/range reported (e.g., confidence interval, SD, etc.) | Variation reported | SD, SE, etc. |
| 37 | Magnitude for variation/range (pre-processing) | Pre-processing variation value |  |
| 38 | Magnitude of measurement of milk component (Post-processing) | Post-processing value |  |
| 39 | Magnitude for variation/range (post-processing) | Post-processing variation value |  |
| 40 | Protein unit | Unit of analyte reported |  |
| 41 | Percent reduction (%, where applicable) | N/A |  |
| 42 | Percent Retention | % retention of protein |  |
| 43 | Value for variation in percent retention/reduction (%) | N/A |  |
| 44 | Assay | Analytical assay |  |
| 45 | LOD | Limit of detection for bacteria |  |
| 46 | LOD unit | N/A |  |
| 47 | Bacteria | Bacteria name |  |
| 48 | Subspecies | N/A |  |
| 49 | Brand number | Bacteria brand number used |  |
| 50 | Origin |  | Native or inoculated |
| 51 | Bacteria Phase | Growth phase of bacteria (if applicable) |  |
| 52 | Type of value reported (e.g, mean, median, etc.) | Statistical reported value |  |
| 53 | Measurement of bacteria (pre-processing) | N/A |  |
| 54 | Type of variation/range reported for bacteria (e.g., CI, SD, etc) | N/A |  |
| 55 | Value for variation/range for bacteria/viruses (pre-processing) | N/A |  |
| 56 | Measurement of bacteria (post-processing) | N/A |  |
| 57 | Value for variation for bacteria (post-processing) | N/A |  |
| 58 | Unit of measurement for bacterial reduction | N/A |  |
| 59 | Reduction | Reduction (CFU/ml) |  |
| 60 | Value for variation/range for bacteria reduction | N/A |  |
| 61 | Concerns | Reasons that paper may be outlier based on methodology |  |
| 62 | Other comments | Other comments if applicable |  |

**Supplementary Table 4:** Bacteria table including major operating parameters (pressure, temperature, time) and reductions of bacteria (CFU/mL). Green represents >5-log reductions, red indicates results that reached limit of detection as written in article.

| **Pressure (MPa)** | **Time (min)** | **Holding temperature (˚C)** | **Measurement of bacteria (pre-processing)** | **Measurement of bacterial reduction** | **First author, year** |
| --- | --- | --- | --- | --- | --- |
| ***Aeromonas hydrophila*** | | | | | |
| 250 | 10 | 25 | 9 | 2.76 | Duraes-Carvalho, 2012 |
| 250 | 15 | 0 | 9 | 5.52 | Duraes-Carvalho, 2012 |
| 250 | 15 | 20 | 9 | 5.04 | Duraes-Carvalho, 2012 |
| 250 | 15 | 25 | 9 | 5.05 | Duraes-Carvalho, 2012 |
| 250 | 15 | 25 | 9 | 5.99 | Duraes-Carvalho, 2012 |
| 250 | 15 | 30 | 9 | 7.97 | Duraes-Carvalho, 2012 |
| 250 | 15 | 40 | 9 | 9 | Duraes-Carvalho, 2012 |
| 250 | 20 | 25 | 9 | 4.68 | Duraes-Carvalho, 2012 |
| 250 | 30 | 25 | 9 | 9 | Duraes-Carvalho, 2012 |
| 350 | 1 | 25 | 9 | 0.05 | Duraes-Carvalho, 2012 |
| 350 | 2 | 25 | 9 | 0 | Duraes-Carvalho, 2012 |
| 350 | 8 | 25 | 9 | 9 | Duraes-Carvalho, 2012 |
| 350 | 16 | 25 | 9 | 9 | Duraes-Carvalho, 2012 |
| 350 | 32 | 25 | 9 | 9 | Duraes-Carvalho, 2012 |
| ***Campylobacter jejuni*** | | | | | |
| 50 | 10 | 25 | 8 | 0 | Soloman, 2004 |
| 50 | 10 | 25 | 8 | 0.15 | Soloman, 2004 |
| 100 | 10 | 25 | 8 | 0 | Soloman, 2004 |
| 100 | 10 | 25 | 8 | 0.15 | Soloman, 2004 |
| 200 | 10 | 20 | 8 | 0.05 | Martinez-Rodriguez, 2005 |
| 200 | 10 | 20 | 8 | 0.09 | Martinez-Rodriguez, 2005 |
| 200 | 10 | 20 | 8 | 0.64 | Martinez-Rodriguez, 2005 |
| 200 | 10 | 20 | 8 | 1.56 | Martinez-Rodriguez, 2005 |
| 200 | 10 | 25 | 8 | 0.1 | Soloman, 2004 |
| 200 | 10 | 25 | 8 | 0.13 | Soloman, 2004 |
| 250 | 10 | 20 | 8 | 0.13 | Martinez-Rodriguez, 2005 |
| 250 | 10 | 20 | 8 | 0.64 | Martinez-Rodriguez, 2005 |
| 250 | 10 | 25 | 8 | 0.15 | Soloman, 2004 |
| 250 | 10 | 25 | 8 | 0.23 | Soloman, 2004 |
| 300 | 10 | 20 | 8 | 0.44 | Martinez-Rodriguez, 2005 |
| 300 | 10 | 20 | 8 | 1.02 | Martinez-Rodriguez, 2005 |
| 300 | 10 | 25 | 8 | 0.38 | Soloman, 2004 |
| 300 | 10 | 25 | 8 | 1.21 | Soloman, 2004 |
| 325 | 10 | 25 | 8 | 1.31 | Soloman, 2004 |
| 325 | 10 | 25 | 8 | 2.28 | Soloman, 2004 |
| 350 | 10 | 25 | 8 | 2.41 | Soloman, 2004 |
| 350 | 10 | 25 | 8 | 4.05 | Soloman, 2004 |
| 375 | 10 | 25 | 8 | 7.97 | Soloman, 2004 |
| 375 | 10 | 25 | 8 | 8 | Soloman, 2004 |
| ***Escherichia coli*** | | | | | |
| 100 | 15 | 10 | 9 | 0.03 | Patterson, 1998 |
| 100 | 15 | 10 | 9 | 0.03 | Patterson, 1998 |
| 100 | 15 | 20 | 9 | 0 | Patterson, 1998 |
| 100 | 15 | 20 | 9 | 0 | Patterson, 1998 |
| 100 | 15 | 40 | 9 | 0 | Patterson, 1998 |
| 100 | 15 | 40 | 9 | 0 | Patterson, 1998 |
| 100 | 15 | 50 | 9 | 0 | Patterson, 1998 |
| 100 | 15 | 50 | 9 | 0.3 | Patterson, 1998 |
| 100 | 15 | 55 | 9 | 0.83 | Patterson, 1998 |
| 100 | 15 | 55 | 9 | 0.93 | Patterson, 1998 |
| 100 | 15 | 60 | 9 | 1.92 | Patterson, 1998 |
| 100 | 15 | 60 | 9 | 2.18 | Patterson, 1998 |
| 200 | 0.1 | 25 | 6 | 0.5 | Ramaswamy, 2009 |
| 200 | 5 | 20 | 8.3 | 0.26 | Park, 2009 |
| 200 | 5 | 30 | 8.3 | 0.93 | Park, 2009 |
| 200 | 5 | 40 | 8.3 | 3.24 | Park, 2009 |
| 200 | 5 | 50 | 8.3 | 3.27 | Park, 2009 |
| 200 | 5 | not reported | 6.66 | 0.26 | Liu, 2017 |
| 200 | 10 | 21.5 | 9 | 0.02 | Chen, 2006 |
| 200 | 15 | 10 | 9 | 0 | Patterson, 1998 |
| 200 | 15 | 10 | 9 | 0.12 | Patterson, 1998 |
| 200 | 15 | 20 | 9 | 0.08 | Patterson, 1998 |
| 200 | 15 | 20 | 9 | 0.19 | Patterson, 1998 |
| 200 | 15 | 20 | 8.3 | 0.52 | Park, 2009 |
| 200 | 15 | 25 | 6 | 1.2 | Ramaswamy, 2009 |
| 200 | 15 | 30 | 8.3 | 1.14 | Park, 2009 |
| 200 | 15 | 40 | 9 | 0 | Patterson, 1998 |
| 200 | 15 | 40 | 9 | 0.05 | Patterson, 1998 |
| 200 | 15 | 40 | 8.3 | 3.54 | Park, 2009 |
| 200 | 15 | 50 | 9 | 0.35 | Patterson, 1998 |
| 200 | 15 | 50 | 9 | 0.73 | Patterson, 1998 |
| 200 | 15 | 50 | 8.3 | 5.07 | Park, 2009 |
| 200 | 15 | 55 | 9 | 2.32 | Patterson, 1998 |
| 200 | 15 | 55 | 9 | 4.93 | Patterson, 1998 |
| 200 | 15 | 60 | 9 | 7.92 | Patterson, 1998 |
| 200 | 15 | 60 | 9 | 7.93 | Patterson, 1998 |
| 200 | 30 | 20 | 8.3 | 0.56 | Park, 2009 |
| 200 | 30 | 30 | 8.3 | 1.36 | Park, 2009 |
| 200 | 30 | 40 | 8.3 | 4.23 | Park, 2009 |
| 200 | 30 | 50 | 8.3 | 7.91 | Park, 2009 |
| 250 | 10 | 21.5 | 9 | 0.12 | Chen, 2006 |
| 300 | 0.1 | 25 | 6 | 0.5 | Ramaswamy, 2009 |
| 300 | 0.1 | 25 | 6 | 1.07 | Ramaswamy, 2009 |
| 300 | 5 | 0 | 8 | 1.83 | Bulut, 2014 |
| 300 | 5 | 0 | 8 | 5.33 | Bulut, 2014 |
| 300 | 5 | 25 | 6 | 1 | Ramaswamy, 2009 |
| 300 | 5 | not reported | 6.66 | 2.14 | Liu, 2017 |
| 300 | 5 | not reported | 6.65 | 2.14 | Liu, 2017 |
| 300 | 5 | not reported | 6.65 | 3.25 | Liu, 2017 |
| 300 | 5 | not reported | 6.65 | 3.35 | Liu, 2017 |
| 300 | 5 | not reported | 6.65 | 3.4 | Liu, 2017 |
| 300 | 5 | not reported | 6.65 | 3.52 | Liu, 2017 |
| 300 | 5 | not reported | 6.65 | 3.59 | Liu, 2017 |
| 300 | 5 | not reported | 6.65 | 3.95 | Liu, 2017 |
| 300 | 5 | not reported | 6.65 | 4 | Liu, 2017 |
| 300 | 8 | 25 | 6 | 2.72 | Ramaswamy, 2009 |
| 300 | 10 | 21.5 | 9 | 0.12 | Chen, 2006 |
| 300 | 15 | 10 | 9 | 0.35 | Patterson, 1998 |
| 300 | 15 | 10 | 9 | 0.56 | Patterson, 1998 |
| 300 | 15 | 20 | 9 | 0.03 | Garcia-Graells, 1999 |
| 300 | 15 | 20 | 9 | 0.1 | Garcia-Graells, 1999 |
| 300 | 15 | 20 | 9 | 0.14 | Patterson, 1998 |
| 300 | 15 | 20 | 9 | 0.19 | Patterson, 1998 |
| 300 | 15 | 20 | 9 | 0.3 | Garcia-Graells, 1999 |
| 300 | 15 | 20 | 9 | 0.35 | Garcia-Graells, 2000 |
| 300 | 15 | 20 | 9 | 0.35 | Garcia-Graells, 2000 |
| 300 | 15 | 20 | 9 | 0.35 | Garcia-Graells, 2000 |
| 300 | 15 | 20 | 9 | 0.5 | Garcia-Graells, 1999 |
| 300 | 15 | 40 | 9 | 0.03 | Patterson, 1998 |
| 300 | 15 | 40 | 9 | 0.68 | Patterson, 1998 |
| 300 | 15 | 50 | 9 | 1.07 | Patterson, 1998 |
| 300 | 15 | 50 | 9 | 3.4 | Patterson, 1998 |
| 300 | 15 | 55 | 9 | 7.82 | Patterson, 1998 |
| 300 | 15 | 55 | 9 | 7.86 | Patterson, 1998 |
| 300 | 16 | 25 | 6 | 4.03 | Ramaswamy, 2009 |
| 300 | 30 | 20 | 5.84 | 2.59 | Foster, 2016 |
| 300 | 45 | 20 | 5.84 | 3.77 | Foster, 2016 |
| 300 | 60 | 20 | 5.84 | 4.59 | Foster, 2016 |
| 350 | 5 | 22 | 7 | 0.39 | Buzrul, 2009 |
| 350 | 5 | 22 | 7 | 1.32 | Buzrul, 2009 |
| 350 | 5 | 22 | 7 | 2.29 | Buzrul, 2009 |
| 350 | 10 | 21.5 | 9 | 0.1 | Chen, 2006 |
| 350 | 15 | 25 | 7 | 0.84 | Nakimbugwe, 2006 |
| 350 | 15 | 25 | 7 | 0.87 | Nakimbugwe, 2006 |
| 350 | 15 | 25 | 7 | 1.33 | Nakimbugwe, 2006 |
| 400 | 0.1 | 25 | 6 | 1.05 | Ramaswamy, 2009 |
| 400 | 0.1 | 25 | 6 | 1.41 | Ramaswamy, 2009 |
| 400 | 1 | 18 | 8 | 1 | Stratakos, 2019 |
| 400 | 2.92 | not reported | 9 | 2.19 | Li, 2020 |
| 400 | 2.92 | not reported | 9 | 2.85 | Li, 2020 |
| 400 | 3 | 18 | 8 | 1.05 | Stratakos, 2019 |
| 400 | 4 | 25 | 6 | 3.81 | Ramaswamy, 2009 |
| 400 | 4 | 25 | 6 | 4.41 | Ramaswamy, 2009 |
| 400 | 5 | 18 | 8 | 2.59 | Stratakos, 2019 |
| 400 | 5 | 22 | 7 | 0.52 | Buzrul, 2009 |
| 400 | 5 | 22 | 7 | 2.22 | Buzrul, 2009 |
| 400 | 5 | 22 | 7 | 2.74 | Buzrul, 2009 |
| 400 | 5 | not reported | 6.66 | 6.64 | Liu, 2017 |
| 400 | 8 | 25 | 6 | 4.61 | Ramaswamy, 2009 |
| 400 | 8 | 25 | 6 | 4.99 | Ramaswamy, 2009 |
| 400 | 10 | 20 | 5.84 | 5.65 | Foster, 2016 |
| 400 | 10 | 21.5 | 9 | 0.14 | Chen, 2006 |
| 400 | 15 | 10 | 9 | 0.72 | Patterson, 1998 |
| 400 | 15 | 10 | 9 | 1.06 | Patterson, 1998 |
| 400 | 15 | 20 | 9 | 0.19 | Patterson, 1998 |
| 400 | 15 | 20 | 9 | 0.25 | Garcia-Graells, 2000 |
| 400 | 15 | 20 | 9 | 0.3 | Garcia-Graells, 1999 |
| 400 | 15 | 20 | 9 | 0.3 | Garcia-Graells, 1999 |
| 400 | 15 | 20 | 9 | 0.4 | Patterson, 1998 |
| 400 | 15 | 20 | 9 | 0.42 | Garcia-Graells, 2000 |
| 400 | 15 | 20 | 9 | 0.45 | Garcia-Graells, 2000 |
| 400 | 15 | 20 | 9 | 0.5 | Garcia-Graells, 1999 |
| 400 | 15 | 20 | 9 | 0.53 | Garcia-Graells, 2000 |
| 400 | 15 | 20 | 9 | 0.56 | Garcia-Graells, 2000 |
| 400 | 15 | 20 | 9 | 0.58 | Garcia-Graells, 2000 |
| 400 | 15 | 20 | 9 | 0.61 | Garcia-Graells, 2000 |
| 400 | 15 | 20 | 9 | 0.69 | Garcia-Graells, 2000 |
| 400 | 15 | 20 | 9 | 0.7 | Garcia-Graells, 1999 |
| 400 | 15 | 20 | 9 | 0.71 | Garcia-Graells, 2000 |
| 400 | 15 | 20 | 9 | 1.21 | Garcia-Graells, 2000 |
| 400 | 15 | 20 | 9 | 1.36 | Garcia-Graells, 2000 |
| 400 | 15 | 20 | 9 | 1.49 | Garcia-Graells, 2000 |
| 400 | 15 | 20 | 9 | 3.42 | Garcia-Graells, 2000 |
| 400 | 15 | 20 | 9 | 3.7 | Garcia-Graells, 2000 |
| 400 | 15 | 20 | 9 | 3.75 | Garcia-Graells, 2000 |
| 400 | 15 | 20 | 5.84 | 5.84 | Foster, 2016 |
| 400 | 15 | 40 | 9 | 0.56 | Patterson, 1998 |
| 400 | 15 | 40 | 9 | 2.1 | Patterson, 1998 |
| 400 | 15 | 50 | 9 | 4.06 | Patterson, 1998 |
| 400 | 15 | 50 | 9 | 5.44 | Patterson, 1998 |
| 400 | 20 | 20 | 5.84 | 5.84 | Foster, 2016 |
| 400 | 20 | 22 | 7 | 0.94 | Buzrul, 2009 |
| 450 | 10 | 21.5 | 9 | 0.45 | Chen, 2006 |
| 500 | 1 | 18 | 8 | 0.86 | Stratakos, 2019 |
| 500 | 3 | 18 | 8 | 2.06 | Stratakos, 2019 |
| 500 | 5 | 18 | 8 | 3.45 | Stratakos, 2019 |
| 500 | 5 | not reported | 6.66 | 6.66 | Liu, 2017 |
| 500 | 10 | 21.5 | 9 | 0.83 | Chen, 2006 |
| 500 | 15 | 10 | 9 | 0.35 | Patterson, 1998 |
| 500 | 15 | 10 | 9 | 2.09 | Patterson, 1998 |
| 500 | 15 | 20 | 9 | 0.25 | Garcia-Graells, 2000 |
| 500 | 15 | 20 | 9 | 0.29 | Patterson, 1998 |
| 500 | 15 | 20 | 9 | 0.3 | Garcia-Graells, 1999 |
| 500 | 15 | 20 | 9 | 0.32 | Garcia-Graells, 2000 |
| 500 | 15 | 20 | 9 | 0.39 | Garcia-Graells, 2000 |
| 500 | 15 | 20 | 9 | 0.5 | Garcia-Graells, 1999 |
| 500 | 15 | 20 | 9 | 0.6 | Garcia-Graells, 1999 |
| 500 | 15 | 20 | 9 | 0.76 | Patterson, 1998 |
| 500 | 15 | 20 | 9 | 1.4 | Garcia-Graells, 1999 |
| 500 | 15 | 40 | 9 | 2.27 | Patterson, 1998 |
| 500 | 15 | 40 | 9 | 3.48 | Patterson, 1998 |
| 500 | 15 | 50 | 9 | 7.84 | Patterson, 1998 |
| 500 | 15 | 50 | 9 | 7.88 | Patterson, 1998 |
| 550 | 10 | 20 | 9 | 0 | Garcia-Graells, 1999 |
| 550 | 10 | 20 | 9 | 0 | Garcia-Graells, 1999 |
| 550 | 10 | 20 | 9 | 0.02 | Garcia-Graells, 1999 |
| 550 | 10 | 20 | 9 | 0.06 | Garcia-Graells, 1999 |
| 550 | 10 | 20 | 9 | 0.1 | Garcia-Graells, 1999 |
| 550 | 10 | 20 | 9 | 0.35 | Garcia-Graells, 1999 |
| 550 | 10 | 20 | 9 | 0.5 | Garcia-Graells, 1999 |
| 550 | 10 | 20 | 9 | 0.68 | Garcia-Graells, 1999 |
| 550 | 10 | 20 | 9 | 0.68 | Garcia-Graells, 1999 |
| 550 | 10 | 20 | 9 | 0.74 | Garcia-Graells, 1999 |
| 550 | 10 | 20 | 9 | 0.8 | Garcia-Graells, 1999 |
| 550 | 10 | 20 | 9 | 0.86 | Garcia-Graells, 1999 |
| 550 | 10 | 20 | 9 | 1.08 | Garcia-Graells, 1999 |
| 550 | 10 | 20 | 9 | 1.11 | Garcia-Graells, 1999 |
| 550 | 10 | 20 | 9 | 1.19 | Garcia-Graells, 1999 |
| 550 | 10 | 20 | 9 | 1.38 | Garcia-Graells, 1999 |
| 550 | 10 | 20 | 9 | 1.4 | Garcia-Graells, 1999 |
| 550 | 10 | 20 | 9 | 1.42 | Garcia-Graells, 1999 |
| 550 | 10 | 20 | 9 | 1.58 | Garcia-Graells, 1999 |
| 550 | 10 | 20 | 9 | 2.12 | Garcia-Graells, 1999 |
| 550 | 10 | 20 | 9 | 2.26 | Garcia-Graells, 1999 |
| 550 | 10 | 20 | 9 | 2.31 | Garcia-Graells, 1999 |
| 550 | 10 | 20 | 9 | 2.52 | Garcia-Graells, 1999 |
| 550 | 10 | 20 | 9 | 2.63 | Garcia-Graells, 1999 |
| 550 | 10 | 20 | 9 | 3.25 | Garcia-Graells, 1999 |
| 550 | 10 | 20 | 9 | 3.68 | Garcia-Graells, 1999 |
| 550 | 10 | 20 | 9 | 3.95 | Garcia-Graells, 1999 |
| 550 | 10 | 20 | 9 | 4.07 | Garcia-Graells, 1999 |
| 550 | 10 | 20 | 9 | 4.23 | Garcia-Graells, 1999 |
| 550 | 10 | 20 | 9 | 4.34 | Garcia-Graells, 1999 |
| 550 | 10 | 20 | 9 | 4.44 | Garcia-Graells, 1999 |
| 550 | 10 | 20 | 9 | 4.6 | Garcia-Graells, 1999 |
| 550 | 10 | 20 | 9 | 4.75 | Garcia-Graells, 1999 |
| 550 | 10 | 20 | 9 | 5.29 | Garcia-Graells, 1999 |
| 550 | 10 | 20 | 9 | 5.38 | Garcia-Graells, 1999 |
| 550 | 10 | 20 | 9 | 5.46 | Garcia-Graells, 1999 |
| 550 | 10 | 20 | 9 | 5.9 | Garcia-Graells, 1999 |
| 550 | 10 | 20 | 9 | 6.08 | Garcia-Graells, 1999 |
| 550 | 10 | 20 | 9 | 6.08 | Garcia-Graells, 1999 |
| 550 | 10 | 20 | 9 | 6.3 | Garcia-Graells, 1999 |
| 550 | 10 | 20 | 9 | 7.13 | Garcia-Graells, 1999 |
| 550 | 10 | 20 | 9 | 7.72 | Garcia-Graells, 1999 |
| 550 | 10 | 20 | 9 | 7.76 | Garcia-Graells, 1999 |
| 550 | 10 | 20 | 9 | 8.07 | Garcia-Graells, 1999 |
| 550 | 10 | 20 | 9 | 8.07 | Garcia-Graells, 1999 |
| 550 | 10 | 20 | 9 | 8.12 | Garcia-Graells, 1999 |
| 550 | 10 | 20 | 9 | 8.14 | Garcia-Graells, 1999 |
| 550 | 10 | 21.5 | 9 | 2.26 | Chen, 2006 |
| 550 | 15 | 10 | 8 | 0.4 | Garcia-Graells, 1999 |
| 550 | 15 | 10 | 8 | 0.6 | Garcia-Graells, 1999 |
| 550 | 15 | 10 | 8 | 0.6 | Garcia-Graells, 1999 |
| 550 | 15 | 10 | 8 | 1 | Garcia-Graells, 1999 |
| 550 | 15 | 20 | 8 | 0.1 | Garcia-Graells, 1999 |
| 550 | 15 | 20 | 8 | 0.4 | Garcia-Graells, 1999 |
| 550 | 15 | 20 | 8 | 0.5 | Garcia-Graells, 1999 |
| 550 | 15 | 20 | 8 | 0.5 | Garcia-Graells, 1999 |
| 550 | 15 | 20 | 9 | 1.76 | Garcia-Graells, 2000 |
| 550 | 15 | 20 | 9 | 1.81 | Garcia-Graells, 2000 |
| 550 | 15 | 20 | 9 | 2.03 | Garcia-Graells, 2000 |
| 550 | 15 | 20 | 9 | 2.15 | Garcia-Graells, 2000 |
| 550 | 15 | 20 | 9 | 2.39 | Garcia-Graells, 2000 |
| 550 | 15 | 20 | 9 | 2.44 | Garcia-Graells, 2000 |
| 550 | 15 | 20 | 9 | 3.05 | Garcia-Graells, 2000 |
| 550 | 15 | 20 | 9 | 3.34 | Garcia-Graells, 2000 |
| 550 | 15 | 20 | 9 | 3.43 | Garcia-Graells, 2000 |
| 550 | 15 | 20 | 9 | 4.98 | Garcia-Graells, 2000 |
| 550 | 15 | 20 | 9 | 5.08 | Garcia-Graells, 2000 |
| 550 | 15 | 20 | 9 | 5.82 | Garcia-Graells, 2000 |
| 550 | 15 | 30 | 8 | 0.3 | Garcia-Graells, 1999 |
| 550 | 15 | 30 | 8 | 0.6 | Garcia-Graells, 1999 |
| 550 | 15 | 30 | 8 | 1.1 | Garcia-Graells, 1999 |
| 550 | 15 | 30 | 8 | 1.7 | Garcia-Graells, 1999 |
| 550 | 15 | 40 | 8 | 0.2 | Garcia-Graells, 1999 |
| 550 | 15 | 40 | 8 | 1.1 | Garcia-Graells, 1999 |
| 550 | 15 | 40 | 8 | 1.7 | Garcia-Graells, 1999 |
| 550 | 15 | 40 | 8 | 3.1 | Garcia-Graells, 1999 |
| 550 | 15 | 50 | 8 | 2.4 | Garcia-Graells, 1999 |
| 550 | 15 | 50 | 8 | 2.4 | Garcia-Graells, 1999 |
| 550 | 15 | 50 | 8 | 4.7 | Garcia-Graells, 1999 |
| 550 | 15 | 50 | 8 | 7 | Garcia-Graells, 1999 |
| 550 | 30 | 20 | 9 | 0.07 | Garcia-Graells, 1999 |
| 550 | 30 | 20 | 9 | 0.12 | Garcia-Graells, 1999 |
| 550 | 30 | 20 | 9 | 0.27 | Garcia-Graells, 1999 |
| 550 | 30 | 20 | 9 | 0.33 | Garcia-Graells, 1999 |
| 550 | 30 | 20 | 9 | 0.74 | Garcia-Graells, 1999 |
| 550 | 30 | 20 | 9 | 0.9 | Garcia-Graells, 1999 |
| 550 | 30 | 20 | 9 | 1.1 | Garcia-Graells, 1999 |
| 550 | 30 | 20 | 9 | 1.59 | Garcia-Graells, 1999 |
| 550 | 30 | 20 | 9 | 1.67 | Garcia-Graells, 1999 |
| 550 | 30 | 20 | 9 | 1.76 | Garcia-Graells, 1999 |
| 550 | 30 | 20 | 9 | 2.19 | Garcia-Graells, 1999 |
| 550 | 30 | 20 | 9 | 2.19 | Garcia-Graells, 1999 |
| 550 | 30 | 20 | 9 | 2.32 | Garcia-Graells, 1999 |
| 550 | 30 | 20 | 9 | 2.82 | Garcia-Graells, 1999 |
| 550 | 30 | 20 | 9 | 5.22 | Garcia-Graells, 1999 |
| 550 | 30 | 20 | 9 | 5.24 | Garcia-Graells, 1999 |
| 600 | 1 | 18 | 8 | 3.69 | Stratakos, 2019 |
| 600 | 1 | 45 | 9 | 0.29 | Guan, 2006 |
| 600 | 2 | 4 | 9 | 0.45 | Guan, 2006 |
| 600 | 2 | 21 | 9 | 0.52 | Guan, 2006 |
| 600 | 2 | 21.5 | 9 | 0.93 | Chen, 2007 |
| 600 | 3 | 4 | 7 | 3.88 | Syed, 2013 |
| 600 | 3 | 4 | 7 | 4.15 | Syed, 2013 |
| 600 | 3 | 4 | 7 | 4.22 | Syed, 2013 |
| 600 | 3 | 4 | 7 | 4.23 | Syed, 2013 |
| 600 | 3 | 4 | 7 | 4.45 | Syed, 2013 |
| 600 | 3 | 18 | 8 | 5.7 | Stratakos, 2019 |
| 600 | 3 | 45 | 9 | 0.86 | Guan, 2006 |
| 600 | 4 | 4 | 9 | 1.42 | Guan, 2006 |
| 600 | 4 | 21 | 9 | 2.01 | Guan, 2006 |
| 600 | 4 | 21.5 | 9 | 1.49 | Chen, 2007 |
| 600 | 5 | 18 | 8 | 6.8 | Stratakos, 2019 |
| 600 | 5 | 20 | 8 | 0.92 | Patterson, 1995 |
| 600 | 6 | 21.5 | 9 | 3.31 | Chen, 2007 |
| 600 | 6 | 45 | 9 | 3.74 | Guan, 2006 |
| 600 | 8 | 4 | 9 | 2.66 | Guan, 2006 |
| 600 | 8 | 21 | 9 | 4.29 | Guan, 2006 |
| 600 | 8 | 21.5 | 9 | 4.12 | Chen, 2007 |
| 600 | 10 | 4 | 9 | 4.11 | Guan, 2006 |
| 600 | 10 | 20 | 8 | 1.11 | Patterson, 1995 |
| 600 | 10 | 21 | 9 | 6.11 | Guan, 2006 |
| 600 | 10 | 21.5 | 9 | 5.89 | Chen, 2007 |
| 600 | 10 | 21.5 | 9 | 6.24 | Chen, 2006 |
| 600 | 10 | 45 | 9 | 5.08 | Guan, 2006 |
| 600 | 12 | 21 | 9 | 7.08 | Guan, 2006 |
| 600 | 15 | 10 | 9 | 0.8 | Patterson, 1998 |
| 600 | 15 | 10 | 9 | 5.82 | Patterson, 1998 |
| 600 | 15 | 20 | 8 | 0.06 | Garcia-Graells, 1999 |
| 600 | 15 | 20 | 9 | 0.1 | Garcia-Graells, 1999 |
| 600 | 15 | 20 | 8 | 0.15 | Garcia-Graells, 1999 |
| 600 | 15 | 20 | 8 | 0.26 | Garcia-Graells, 1999 |
| 600 | 15 | 20 | 8 | 0.28 | Garcia-Graells, 1999 |
| 600 | 15 | 20 | 9 | 0.3 | Garcia-Graells, 1999 |
| 600 | 15 | 20 | 9 | 0.3 | Garcia-Graells, 1999 |
| 600 | 15 | 20 | 8 | 0.32 | Garcia-Graells, 1999 |
| 600 | 15 | 20 | 8 | 0.34 | Garcia-Graells, 1999 |
| 600 | 15 | 20 | 9 | 0.4 | Garcia-Graells, 1999 |
| 600 | 15 | 20 | 8 | 0.41 | Garcia-Graells, 1999 |
| 600 | 15 | 20 | 8 | 0.47 | Garcia-Graells, 1999 |
| 600 | 15 | 20 | 8 | 0.52 | Garcia-Graells, 1999 |
| 600 | 15 | 20 | 9 | 0.6 | Garcia-Graells, 1999 |
| 600 | 15 | 20 | 9 | 0.69 | Patterson, 1998 |
| 600 | 15 | 20 | 9 | 0.8 | Garcia-Graells, 1999 |
| 600 | 15 | 20 | 8 | 0.8 | Garcia-Graells, 1999 |
| 600 | 15 | 20 | 8 | 0.81 | Garcia-Graells, 1999 |
| 600 | 15 | 20 | 8 | 0.82 | Garcia-Graells, 1999 |
| 600 | 15 | 20 | 8 | 1 | Garcia-Graells, 1999 |
| 600 | 15 | 20 | 8 | 1.01 | Garcia-Graells, 1999 |
| 600 | 15 | 20 | 8 | 1.09 | Garcia-Graells, 1999 |
| 600 | 15 | 20 | 8 | 1.18 | Garcia-Graells, 1999 |
| 600 | 15 | 20 | 8 | 1.27 | Garcia-Graells, 1999 |
| 600 | 15 | 20 | 9 | 1.3 | Garcia-Graells, 1999 |
| 600 | 15 | 20 | 9 | 1.3 | Garcia-Graells, 1999 |
| 600 | 15 | 20 | 8 | 1.32 | Garcia-Graells, 1999 |
| 600 | 15 | 20 | 8 | 1.32 | Patterson, 1995 |
| 600 | 15 | 20 | 8 | 1.35 | Garcia-Graells, 1999 |
| 600 | 15 | 20 | 8 | 1.35 | Garcia-Graells, 1999 |
| 600 | 15 | 20 | 9 | 1.4 | Garcia-Graells, 1999 |
| 600 | 15 | 20 | 9 | 1.4 | Garcia-Graells, 1999 |
| 600 | 15 | 20 | 8 | 1.4 | Garcia-Graells, 1999 |
| 600 | 15 | 20 | 8 | 1.44 | Garcia-Graells, 1999 |
| 600 | 15 | 20 | 9 | 1.5 | Garcia-Graells, 1999 |
| 600 | 15 | 20 | 8 | 1.5 | Garcia-Graells, 1999 |
| 600 | 15 | 20 | 9 | 1.54 | Garcia-Graells, 2000 |
| 600 | 15 | 20 | 9 | 1.6 | Garcia-Graells, 1999 |
| 600 | 15 | 20 | 9 | 1.6 | Garcia-Graells, 1999 |
| 600 | 15 | 20 | 9 | 1.6 | Garcia-Graells, 1999 |
| 600 | 15 | 20 | 8 | 1.62 | Garcia-Graells, 1999 |
| 600 | 15 | 20 | 8 | 1.68 | Garcia-Graells, 1999 |
| 600 | 15 | 20 | 8 | 1.69 | Garcia-Graells, 1999 |
| 600 | 15 | 20 | 8 | 1.75 | Garcia-Graells, 1999 |
| 600 | 15 | 20 | 9 | 1.75 | Garcia-Graells, 2000 |
| 600 | 15 | 20 | 8 | 1.76 | Garcia-Graells, 1999 |
| 600 | 15 | 20 | 8 | 1.81 | Garcia-Graells, 1999 |
| 600 | 15 | 20 | 8 | 1.84 | Garcia-Graells, 1999 |
| 600 | 15 | 20 | 8 | 1.88 | Garcia-Graells, 1999 |
| 600 | 15 | 20 | 8 | 2.02 | Garcia-Graells, 1999 |
| 600 | 15 | 20 | 8 | 2.03 | Garcia-Graells, 1999 |
| 600 | 15 | 20 | 9 | 2.04 | Garcia-Graells, 2000 |
| 600 | 15 | 20 | 8 | 2.1 | Garcia-Graells, 1999 |
| 600 | 15 | 20 | 8 | 2.18 | Garcia-Graells, 1999 |
| 600 | 15 | 20 | 8 | 2.24 | Garcia-Graells, 1999 |
| 600 | 15 | 20 | 9 | 2.3 | Garcia-Graells, 1999 |
| 600 | 15 | 20 | 8 | 2.36 | Garcia-Graells, 1999 |
| 600 | 15 | 20 | 8 | 2.59 | Garcia-Graells, 1999 |
| 600 | 15 | 20 | 8 | 2.61 | Garcia-Graells, 1999 |
| 600 | 15 | 20 | 8 | 2.61 | Garcia-Graells, 1999 |
| 600 | 15 | 20 | 8 | 2.94 | Garcia-Graells, 1999 |
| 600 | 15 | 20 | 9 | 2.99 | Patterson, 1998 |
| 600 | 15 | 20 | 9 | 3 | Garcia-Graells, 1999 |
| 600 | 15 | 20 | 8 | 3.19 | Garcia-Graells, 1999 |
| 600 | 15 | 20 | 8 | 3.4 | Garcia-Graells, 1999 |
| 600 | 15 | 20 | 8 | 4.12 | Garcia-Graells, 1999 |
| 600 | 15 | 20 | 8 | 4.17 | Garcia-Graells, 1999 |
| 600 | 15 | 20 | 8 | 4.35 | Garcia-Graells, 1999 |
| 600 | 15 | 20 | 8 | 4.6 | Garcia-Graells, 1999 |
| 600 | 15 | 20 | 8 | 5.64 | Garcia-Graells, 1999 |
| 600 | 15 | 21.5 | 9 | 5.65 | Chen, 2007 |
| 600 | 15 | 40 | 9 | 2.48 | Patterson, 1998 |
| 600 | 15 | 40 | 9 | 6.8 | Patterson, 1998 |
| 600 | 15 | 45 | 9 | 5.62 | Guan, 2006 |
| 600 | 16 | 4 | 9 | 5.59 | Guan, 2006 |
| 600 | 16 | 21 | 9 | 7.2 | Guan, 2006 |
| 600 | 18 | 21 | 9 | 7.78 | Guan, 2006 |
| 600 | 18 | 45 | 9 | 8.48 | Guan, 2006 |
| 600 | 20 | 20 | 8 | 1.32 | Patterson, 1995 |
| 600 | 20 | 21 | 9 | 8.66 | Guan, 2006 |
| 600 | 20 | 21.5 | 9 | 6.13 | Chen, 2007 |
| 600 | 24 | 4 | 9 | 6.09 | Guan, 2006 |
| 600 | 25 | 20 | 8 | 2.2 | Patterson, 1995 |
| 600 | 30 | 4 | 9 | 6.5 | Guan, 2006 |
| 600 | 30 | 20 | 8 | 2.01 | Patterson, 1995 |
| 600 | 30 | 21.5 | 9 | 7.59 | Chen, 2007 |
| 650 | 10 | 21.5 | 9 | 7.14 | Chen, 2006 |
| 690 | 10 | 21.5 | 9 | 7.69 | Chen, 2006 |
| 700 | 15 | 20 | 9 | 0.4 | Garcia-Graells, 1999 |
| 700 | 15 | 20 | 9 | 0.6 | Garcia-Graells, 1999 |
| 700 | 15 | 20 | 9 | 0.9 | Garcia-Graells, 1999 |
| 700 | 15 | 20 | 9 | 1.42 | Patterson, 1998 |
| 700 | 15 | 20 | 9 | 2.3 | Garcia-Graells, 1999 |
| 700 | 15 | 20 | 9 | 5 | Patterson, 1998 |
| 700 | 15 | 40 | 9 | 7.81 | Patterson, 1998 |
| 700 | 15 | 40 | 9 | 7.88 | Patterson, 1998 |
| ***Francisella tularensis*** | | | | | |
| 300 | 1 | 25 | 6.4 | 0.18 | Schlesser, 2009 |
| 300 | 2 | 10 | 5.6 | 0.27 | Schlesser, 2009 |
| 300 | 2 | 25 | 6.4 | 0.35 | Schlesser, 2009 |
| 300 | 3 | 10 | 5.6 | 0.38 | Schlesser, 2009 |
| 300 | 3 | 25 | 6.4 | 0.47 | Schlesser, 2009 |
| 300 | 4 | 10 | 5.6 | 2.12 | Schlesser, 2009 |
| 300 | 4 | 25 | 6.4 | 3.13 | Schlesser, 2009 |
| 300 | 6 | 10 | 5.6 | 3.67 | Schlesser, 2009 |
| 300 | 6 | 25 | 6.4 | 4.47 | Schlesser, 2009 |
| 500 | 0.5 | 10 | 5.58 | 5.58 | Schlesser, 2009 |
| 500 | 0.5 | 25 | 6.65 | 6.65 | Schlesser, 2009 |
| 500 | 1 | 10 | 5.58 | 5.58 | Schlesser, 2009 |
| 500 | 1 | 25 | 6.65 | 6.65 | Schlesser, 2009 |
| ***Listeria monocytogenes*** | | | | | |
| 0.1 | 10 | 25 | 7.1 | 0 | Misiou, 2018 |
| 0.1 | 10 | 25 | 7.1 | 0 | Misiou, 2018 |
| 0.1 | 10 | 25 | 7.1 | 0.02 | Misiou, 2018 |
| 0.1 | 10 | 25 | 7.1 | 1.45 | Misiou, 2018 |
| 150 | 20 | 20 | 9 | 0.08 | Karatzas, 2002 |
| 200 | 5 | 10 | 4 | 0 | Komora, 2020 |
| 200 | 5 | 10 | 4 | 0 | Komora, 2020 |
| 200 | 5 | 10 | 4 | 1.03 | Komora, 2020 |
| 200 | 5 | 10 | 4 | 1.18 | Komora, 2020 |
| 200 | 5 | 10 | 4 | 2.14 | Komora, 2020 |
| 200 | 5 | 10 | 4 | 2.84 | Komora, 2020 |
| 200 | 5 | 10 | 4 | 4.4 | Komora, 2020 |
| 200 | 5 | 10 | 4 | 4.4 | Komora, 2020 |
| 200 | 5 | 20 | 9 | 0.14 | Ramos, 2015 |
| 200 | 5 | not reported | 6.38 | 0.38 | Liu, 2017 |
| 200 | 10 | 20 | 9 | 0.29 | Ramos, 2015 |
| 200 | 10 | 25 | 7.4 | 0 | Misiou, 2018 |
| 200 | 10 | 25 | 7.1 | 0.39 | Misiou, 2018 |
| 200 | 10 | 25 | 7.1 | 0.5 | Misiou, 2018 |
| 200 | 10 | 25 | 7.1 | 1.97 | Misiou, 2018 |
| 200 | 10 | 25 | 7.1 | 3.51 | Misiou, 2018 |
| 200 | 15 | 20 | 9 | 0.34 | Ramos, 2015 |
| 200 | 15 | 35 | 8 | 0.12 | Simpson, 1997 |
| 200 | 15 | 35 | 8 | 0.21 | Simpson, 1997 |
| 200 | 15 | 35 | 5.7 | 3.61 | Simpson, 1997 |
| 200 | 15 | 45 | 8 | 0.12 | Simpson, 1997 |
| 200 | 15 | 45 | 8 | 0.42 | Simpson, 1997 |
| 200 | 15 | 45 | 5.7 | 7.64 | Simpson, 1997 |
| 200 | 15 | 55 | 8 | 5.64 | Simpson, 1997 |
| 200 | 15 | 55 | 8 | 6.72 | Simpson, 1997 |
| 200 | 20 | 20 | 9 | 0.1 | Karatzas, 2002 |
| 250 | 5 | 21.5 | 9 | 0.02 | Chen, 2006 |
| 250 | 5 | 25 | 8.7 | 0.02 | Huang, 2015 |
| 250 | 10 | 21.5 | 9 | 0.12 | Chen, 2006 |
| 250 | 10 | 25 | 8.7 | 0.25 | Huang, 2015 |
| 250 | 20 | 20 | 9 | 0.36 | Karatzas, 2002 |
| 275.6 | 30 | 23 | 8 | 0.08 | Styles, 1991 |
| 275.6 | 60 | 23 | 8 | 0.08 | Styles, 1991 |
| 275.6 | 90 | 23 | 8 | 0.55 | Styles, 1991 |
| 275.6 | 120 | 23 | 8 | 1.02 | Styles, 1991 |
| 300 | 3 | 25 | 7.69 | 1.21 | Dogan, 2004 |
| 300 | 5 | 10 | 4 | 0.12 | Komora, 2020 |
| 300 | 5 | 10 | 4 | 0.2 | Komora, 2020 |
| 300 | 5 | 10 | 4 | 1.03 | Komora, 2020 |
| 300 | 5 | 10 | 4 | 1.24 | Komora, 2020 |
| 300 | 5 | 10 | 4 | 2.8 | Komora, 2020 |
| 300 | 5 | 10 | 4 | 4.38 | Komora, 2020 |
| 300 | 5 | 10 | 4 | 4.4 | Komora, 2020 |
| 300 | 5 | 10 | 4 | 4.4 | Komora, 2020 |
| 300 | 5 | 20 | 9 | 0.1 | Ramos, 2015 |
| 300 | 5 | 21.5 | 9 | 0.12 | Chen, 2006 |
| 300 | 5 | 25 | 8.7 | 0.88 | Huang, 2015 |
| 300 | 5 | not reported | 6.38 | 1.52 | Liu, 2017 |
| 300 | 5 | not reported | 6.86 | 2.09 | Liu, 2017 |
| 300 | 5 | not reported | 6.86 | 2.66 | Liu, 2017 |
| 300 | 5 | not reported | 6.86 | 3.14 | Liu, 2017 |
| 300 | 5 | not reported | 6.86 | 3.39 | Liu, 2017 |
| 300 | 5 | not reported | 6.86 | 3.94 | Liu, 2017 |
| 300 | 5 | not reported | 6.86 | 3.96 | Liu, 2017 |
| 300 | 5 | not reported | 6.86 | 4.24 | Liu, 2017 |
| 300 | 5 | not reported | 6.86 | 4.41 | Liu, 2017 |
| 300 | 6 | 25 | 7.69 | 1.91 | Dogan, 2004 |
| 300 | 10 | 20 | 9 | 0.17 | Ramos, 2015 |
| 300 | 10 | 21.5 | 9 | 0.12 | Chen, 2006 |
| 300 | 10 | 25 | 7.4 | 0.22 | Misiou, 2018 |
| 300 | 10 | 25 | 8.7 | 2.51 | Huang, 2015 |
| 300 | 10 | 25 | 7.69 | 4.15 | Dogan, 2004 |
| 300 | 15 | 20 | 9 | 2.19 | Ramos, 2015 |
| 300 | 15 | 25 | 7.69 | 3.23 | Dogan, 2004 |
| 300 | 20 | 20 | 9 | 0.63 | Karatzas, 2002 |
| 300 | 30 | 25 | 7.69 | 4.41 | Dogan, 2004 |
| 300 | 40 | 25 | 7.69 | 5.28 | Dogan, 2004 |
| 300 | 50 | 25 | 7.69 | 6.46 | Dogan, 2004 |
| 300 | 60 | 21.5 | 9 | 0.42 | Chen, 2004 |
| 300 | 60 | 25 | 7.69 | 6.77 | Dogan, 2004 |
| 300 | 65 | 25 | 7.69 | 7.4 | Dogan, 2004 |
| 300 | 75 | 25 | 7.69 | 7.69 | Dogan, 2004 |
| 300 | 120 | 21.5 | 9 | 1.14 | Chen, 2004 |
| 300 | 180 | 21.5 | 9 | 1.9 | Chen, 2004 |
| 300 | 240 | 21.5 | 9 | 2.7 | Chen, 2004 |
| 300 | 300 | 21.5 | 9 | 3.47 | Chen, 2004 |
| 300 | 360 | 21.5 | 9 | 4.27 | Chen, 2004 |
| 300 | 420 | 21.5 | 9 | 4.99 | Chen, 2004 |
| 300 | 480 | 21.5 | 9 | 5.75 | Chen, 2004 |
| 300 | 540 | 21.5 | 9 | 6.56 | Chen, 2004 |
| 300 | 600 | 21.5 | 9 | 7.36 | Chen, 2004 |
| 310 | 3 | 4 | 5.3 | 1.4 | Allison, 2018 |
| 310 | 6 | 4 | 5.3 | 1.8 | Allison, 2018 |
| 310 | 9 | 4 | 5.3 | 2.5 | Allison, 2018 |
| 310.05 | 30 | 23 | 8 | 0.3 | Styles, 1991 |
| 310.05 | 60 | 23 | 8 | 0.58 | Styles, 1991 |
| 310.05 | 90 | 23 | 8 | 0.97 | Styles, 1991 |
| 310.05 | 120 | 23 | 8 | 2.54 | Styles, 1991 |
| 344.51 | 20 | 23 | 8 | 1.57 | Styles, 1991 |
| 344.51 | 40 | 23 | 8 | 3.7 | Styles, 1991 |
| 344.51 | 40 | 23 | 8 | 5.16 | Styles, 1991 |
| 344.51 | 60 | 23 | 8 | 4.63 | Styles, 1991 |
| 344.51 | 60 | 23 | 8 | 6.1 | Styles, 1991 |
| 344.51 | 80 | 23 | 8 | 5.85 | Styles, 1991 |
| 350 | 5 | 21.5 | 9 | 0.88 | Chen, 2006 |
| 350 | 5 | 22 | 7 | 0.62 | Buzrul, 2009 |
| 350 | 5 | 22 | 7 | 1.28 | Buzrul, 2009 |
| 350 | 5 | 22 | 7 | 1.96 | Buzrul, 2009 |
| 350 | 5 | 25 | 8.7 | 4.31 | Huang, 2015 |
| 350 | 10 | 21.5 | 9 | 0.76 | Chen, 2006 |
| 350 | 10 | 25 | 8.7 | 5.71 | Huang, 2015 |
| 350 | 12 | 4 | 5.3 | 2.6 | Allison, 2018 |
| 350 | 20 | 20 | 9 | 1.49 | Karatzas, 2002 |
| 375 | 5 | 20 | 8 | 0.03 | Simpson, 1997 |
| 375 | 5 | 20 | 8 | 0.04 | Patterson, 1995 |
| 375 | 5 | 20 | 8 | 0.06 | Simpson, 1997 |
| 375 | 5 | 20 | 8 | 0.33 | Simpson, 1997 |
| 375 | 5 | 20 | 8 | 0.42 | Simpson, 1997 |
| 375 | 5 | 20 | 8 | 0.81 | Simpson, 1997 |
| 375 | 5 | 20 | 8 | 1.38 | Simpson, 1997 |
| 375 | 5 | 20 | 8 | 1.47 | Simpson, 1997 |
| 375 | 5 | 20 | 8 | 1.53 | Simpson, 1997 |
| 375 | 5 | 20 | 8 | 2.04 | Simpson, 1997 |
| 375 | 5 | 20 | 8 | 4.16 | Simpson, 1997 |
| 375 | 5 | 20 | 8 | 5.36 | Simpson, 1997 |
| 375 | 5 | 20 | 8 | 5.75 | Simpson, 1997 |
| 375 | 10 | 20 | 8 | 0.04 | Patterson, 1995 |
| 375 | 15 | 20 | 8 | 0.33 | Patterson, 1995 |
| 375 | 15 | 35 | 7.31 | 2.42 | Simpson, 1997 |
| 375 | 15 | 45 | 7.31 | 7.52 | Simpson, 1997 |
| 375 | 20 | 20 | 8 | 0.76 | Patterson, 1995 |
| 375 | 25 | 20 | 8 | 1.39 | Patterson, 1995 |
| 375 | 30 | 20 | 8 | 1.49 | Patterson, 1995 |
| 400 | 0.012 | 20 | 7 | -0.75 | Hayman, 2007 |
| 400 | 0.25 | 20 | 8 | 0.68 | Hayman, 2008 |
| 400 | 0.25 | 20 | 8 | 0.87 | Hayman, 2008 |
| 400 | 0.25 | 20 | 8 | 0.9 | Hayman, 2008 |
| 400 | 0.25 | 20 | 8 | 1.34 | Hayman, 2008 |
| 400 | 0.26 | 20 | 7 | 0.42 | Hayman, 2007 |
| 400 | 0.27 | 20 | 7 | -0.48 | Hayman, 2007 |
| 400 | 0.27 | 20 | 7 | -0.36 | Hayman, 2007 |
| 400 | 0.27 | 20 | 7 | 0.83 | Hayman, 2007 |
| 400 | 0.32 | 60 | 7 | 3.14 | Mishra, 2013 |
| 400 | 0.5 | 20 | 8 | 0.68 | Hayman, 2008 |
| 400 | 0.5 | 20 | 8 | 0.84 | Hayman, 2008 |
| 400 | 0.5 | 20 | 8 | 1.05 | Hayman, 2008 |
| 400 | 0.5 | 20 | 8 | 1.4 | Hayman, 2008 |
| 400 | 0.5 | 22 | 7.33 | 0.61 | Wen, 2009 |
| 400 | 0.51 | 20 | 7 | 0 | Hayman, 2007 |
| 400 | 0.51 | 20 | 7 | 0.67 | Hayman, 2007 |
| 400 | 0.52 | 20 | 7 | 0.55 | Hayman, 2007 |
| 400 | 0.52 | 20 | 7 | 0.6 | Hayman, 2007 |
| 400 | 0.52 | 20 | 7 | 1.74 | Hayman, 2007 |
| 400 | 0.52 | 20 | 7 | 1.79 | Hayman, 2007 |
| 400 | 0.69 | 60 | 7 | 3.44 | Mishra, 2013 |
| 400 | 0.75 | 20 | 7 | 2.29 | Hayman, 2007 |
| 400 | 0.99 | 20 | 7.26 | 1.55 | Hayman, 2007 |
| 400 | 1 | 8 | 6 | 0.43 | McClements, 2001 |
| 400 | 1 | 8 | 6 | 1.28 | McClements, 2001 |
| 400 | 1 | 8 | 6 | 1.43 | McClements, 2001 |
| 400 | 1 | 8 | 6 | 1.82 | McClements, 2001 |
| 400 | 1 | 18 | 8 | 1 | Stratakos, 2019 |
| 400 | 1 | 20 | 8 | 0.71 | Hayman, 2008 |
| 400 | 1 | 20 | 8 | 1.74 | Hayman, 2008 |
| 400 | 1 | 20 | 8 | 2.02 | Hayman, 2008 |
| 400 | 1 | 20 | 7 | 2.26 | Hayman, 2007 |
| 400 | 1 | 20 | 8 | 2.36 | Hayman, 2008 |
| 400 | 1 | 20 | 7 | 3.47 | Hayman, 2007 |
| 400 | 1 | 22 | 7.46 | 0.64 | Wen, 2009 |
| 400 | 1 | 22 | 7.33 | 3.1 | Wen, 2009 |
| 400 | 1 | 25 | 7.69 | 0.8 | Dogan, 2004 |
| 400 | 1 | 25 | 7.88 | 0.96 | Erkmen, 2004 |
| 400 | 1.01 | 20 | 7 | 1.39 | Hayman, 2007 |
| 400 | 1.01 | 20 | 7 | 1.94 | Hayman, 2007 |
| 400 | 1.01 | 20 | 7 | 2.49 | Hayman, 2007 |
| 400 | 1.01 | 20 | 7 | 3.67 | Hayman, 2007 |
| 400 | 1.01 | 60 | 7 | 4.28 | Mishra, 2013 |
| 400 | 1.33 | 60 | 7 | 4.64 | Mishra, 2013 |
| 400 | 1.5 | 20 | 8 | 0.69 | Hayman, 2008 |
| 400 | 1.5 | 20 | 8 | 1.08 | Hayman, 2008 |
| 400 | 1.5 | 20 | 8 | 1.2 | Hayman, 2008 |
| 400 | 1.5 | 20 | 8 | 1.3 | Hayman, 2008 |
| 400 | 1.5 | 20 | 8 | 1.4 | Hayman, 2008 |
| 400 | 1.5 | 20 | 8 | 1.77 | Hayman, 2008 |
| 400 | 1.5 | 20 | 8 | 1.98 | Hayman, 2008 |
| 400 | 1.5 | 20 | 7 | 2.56 | Hayman, 2007 |
| 400 | 1.5 | 20 | 8 | 3.22 | Hayman, 2008 |
| 400 | 1.5 | 20 | 8 | 3.26 | Hayman, 2008 |
| 400 | 1.5 | 20 | 8 | 3.5 | Hayman, 2008 |
| 400 | 1.5 | 20 | 7 | 3.81 | Hayman, 2007 |
| 400 | 1.5 | 20 | 7 | 4.26 | Hayman, 2007 |
| 400 | 1.5 | 20 | 8 | 4.44 | Hayman, 2008 |
| 400 | 1.5 | 20 | 8 | 4.51 | Hayman, 2008 |
| 400 | 1.5 | 22 | 7.46 | 0.86 | Wen, 2009 |
| 400 | 1.5 | 22 | 7.33 | 1.15 | Wen, 2009 |
| 400 | 1.5 | 22 | 7.46 | 2.64 | Wen, 2009 |
| 400 | 1.5 | 22 | 7.33 | 5.25 | Wen, 2009 |
| 400 | 1.51 | 20 | 7 | 3.84 | Hayman, 2007 |
| 400 | 1.51 | 20 | 7 | 5.32 | Hayman, 2007 |
| 400 | 1.52 | 20 | 7 | 4.48 | Hayman, 2007 |
| 400 | 1.65 | 60 | 7 | 6.14 | Mishra, 2013 |
| 400 | 1.92 | 20 | 7.01 | 1.92 | Hayman, 2007 |
| 400 | 2 | 8 | 6 | 2.53 | McClements, 2001 |
| 400 | 2 | 8 | 6 | 2.61 | McClements, 2001 |
| 400 | 2 | 8 | 6 | 3.04 | McClements, 2001 |
| 400 | 2 | 8 | 6 | 3.14 | McClements, 2001 |
| 400 | 2 | 20 | 6.9 | 0.48 | Hayman, 2007 |
| 400 | 2 | 20 | 8 | 1.61 | Hayman, 2008 |
| 400 | 2 | 20 | 7 | 3.01 | Hayman, 2007 |
| 400 | 2 | 20 | 8 | 3.88 | Hayman, 2008 |
| 400 | 2 | 20 | 8 | 4.1 | Hayman, 2008 |
| 400 | 2 | 20 | 7 | 4.16 | Hayman, 2007 |
| 400 | 2 | 20 | 8 | 4.19 | Hayman, 2008 |
| 400 | 2 | 20 | 7 | 4.68 | Hayman, 2007 |
| 400 | 2 | 20 | 7 | 4.83 | Hayman, 2007 |
| 400 | 2 | 20 | 7 | 6.01 | Hayman, 2007 |
| 400 | 2 | 21 | 9 | 3.08 | Shearer, 2010 |
| 400 | 2 | 21 | 9 | 3.17 | Shearer, 2010 |
| 400 | 2 | 21 | 9 | 3.46 | Shearer, 2010 |
| 400 | 2 | 21 | 9 | 3.89 | Shearer, 2010 |
| 400 | 2 | 21 | 9 | 4.37 | Shearer, 2010 |
| 400 | 2 | 21 | 9 | 4.72 | Shearer, 2010 |
| 400 | 2 | 21 | 9 | 4.81 | Shearer, 2010 |
| 400 | 2 | 21 | 9 | 4.9 | Shearer, 2010 |
| 400 | 2 | 22 | 7.46 | 4.19 | Wen, 2009 |
| 400 | 2 | 22 | 7.33 | 6.16 | Wen, 2009 |
| 400 | 2 | 30 | 7 | 4.76 | McClements, 2001 |
| 400 | 2 | 30 | 7 | 4.96 | McClements, 2001 |
| 400 | 2 | 30 | 7 | 6.26 | McClements, 2001 |
| 400 | 2 | 30 | 7 | 6.29 | McClements, 2001 |
| 400 | 2 | 50 | 9 | 5.04 | Chen, 2003b |
| 400 | 2.01 | 20 | 7 | 4.33 | Hayman, 2007 |
| 400 | 2.06 | 20 | 7.26 | 1.78 | Hayman, 2007 |
| 400 | 2.37 | 20 | 7.23 | 1.84 | Hayman, 2007 |
| 400 | 2.5 | 20 | 8 | 1.8 | Hayman, 2008 |
| 400 | 2.5 | 20 | 8 | 4.71 | Hayman, 2008 |
| 400 | 2.5 | 20 | 8 | 4.93 | Hayman, 2008 |
| 400 | 2.5 | 20 | 7 | 5.05 | Hayman, 2007 |
| 400 | 2.5 | 20 | 7 | 5.06 | Hayman, 2007 |
| 400 | 2.5 | 20 | 8 | 5.09 | Hayman, 2008 |
| 400 | 2.5 | 20 | 7 | 7 | Hayman, 2007 |
| 400 | 2.51 | 20 | 7 | 3.71 | Hayman, 2007 |
| 400 | 2.51 | 20 | 7 | 6.6 | Hayman, 2007 |
| 400 | 2.52 | 20 | 7 | 4.66 | Hayman, 2007 |
| 400 | 3 | 8 | 6 | 3.02 | McClements, 2001 |
| 400 | 3 | 8 | 6 | 3.22 | McClements, 2001 |
| 400 | 3 | 8 | 6 | 3.38 | McClements, 2001 |
| 400 | 3 | 8 | 6 | 3.5 | McClements, 2001 |
| 400 | 3 | 18 | 8 | 1.3 | Stratakos, 2019 |
| 400 | 3 | 20 | 7 | 4.98 | Hayman, 2007 |
| 400 | 3 | 20 | 7 | 5.6 | Hayman, 2007 |
| 400 | 3 | 20 | 7 | 5.78 | Hayman, 2007 |
| 400 | 3 | 20 | 7 | 6.93 | Hayman, 2007 |
| 400 | 3 | 20 | 7 | 6.98 | Hayman, 2007 |
| 400 | 3 | 20 | 7 | 7 | Hayman, 2007 |
| 400 | 3 | 22 | 7.46 | 1.28 | Wen, 2009 |
| 400 | 3 | 22 | 7.33 | 3.62 | Wen, 2009 |
| 400 | 3 | 22 | 7.46 | 5.38 | Wen, 2009 |
| 400 | 3 | 22 | 7.33 | 6.87 | Wen, 2009 |
| 400 | 3 | 25 | 7.88 | 1.31 | Erkmen, 2004 |
| 400 | 3 | 25 | 7.69 | 1.81 | Dogan, 2004 |
| 400 | 3 | 43 | 7 | 1.6 | Mishra, 2013 |
| 400 | 3.03 | 20 | 7.26 | 3.74 | Hayman, 2007 |
| 400 | 3.27 | 20 | 6.85 | 1.51 | Hayman, 2007 |
| 400 | 3.32 | 20 | 6.85 | 2.11 | Hayman, 2007 |
| 400 | 3.35 | 20 | 7.23 | 3.57 | Hayman, 2007 |
| 400 | 4 | 8 | 9 | 1.18 | McClements, 2001 |
| 400 | 4 | 8 | 9 | 1.67 | McClements, 2001 |
| 400 | 4 | 8 | 9 | 1.83 | McClements, 2001 |
| 400 | 4 | 8 | 9 | 2.51 | McClements, 2001 |
| 400 | 4 | 8 | 6 | 3.32 | McClements, 2001 |
| 400 | 4 | 8 | 6 | 3.63 | McClements, 2001 |
| 400 | 4 | 8 | 6 | 3.86 | McClements, 2001 |
| 400 | 4 | 8 | 6 | 4.32 | McClements, 2001 |
| 400 | 4 | 20 | 6.9 | 2.01 | Hayman, 2007 |
| 400 | 4 | 20 | 7.01 | 2.98 | Hayman, 2007 |
| 400 | 4 | 20 | 7.26 | 4 | Hayman, 2007 |
| 400 | 4 | 22 | 7.46 | 5.79 | Wen, 2009 |
| 400 | 4 | 30 | 9 | 1.38 | McClements, 2001 |
| 400 | 4 | 30 | 9 | 1.9 | McClements, 2001 |
| 400 | 4 | 30 | 9 | 5.2 | McClements, 2001 |
| 400 | 4 | 30 | 9 | 5.38 | McClements, 2001 |
| 400 | 4 | 30 | 7 | 5.78 | McClements, 2001 |
| 400 | 4 | 30 | 7 | 6.11 | McClements, 2001 |
| 400 | 4 | 30 | 7 | 6.65 | McClements, 2001 |
| 400 | 4 | 30 | 7 | 6.72 | McClements, 2001 |
| 400 | 4 | 50 | 9 | 5.56 | Chen, 2003b |
| 400 | 4.31 | 20 | 7.23 | 5.22 | Hayman, 2007 |
| 400 | 4.5 | 22 | 7.46 | 1.58 | Wen, 2009 |
| 400 | 4.5 | 22 | 7.33 | 4.93 | Wen, 2009 |
| 400 | 5 | 8 | 6 | 3.43 | McClements, 2001 |
| 400 | 5 | 8 | 6 | 3.78 | McClements, 2001 |
| 400 | 5 | 8 | 6 | 4.3 | McClements, 2001 |
| 400 | 5 | 8 | 6 | 4.4 | McClements, 2001 |
| 400 | 5 | 18 | 8 | 2.22 | Stratakos, 2019 |
| 400 | 5 | 20 | 9 | 3.51 | Ramos, 2015 |
| 400 | 5 | 21.5 | 9 | 3.6 | Chen, 2006 |
| 400 | 5 | 22 | 7 | 0.59 | Buzrul, 2009 |
| 400 | 5 | 22 | 7 | 2.17 | Buzrul, 2009 |
| 400 | 5 | 22 | 7 | 2.74 | Buzrul, 2009 |
| 400 | 5 | 25 | 7.88 | 1.93 | Erkmen, 2004 |
| 400 | 5 | 25 | 8.7 | 6.31 | Huang, 2015 |
| 400 | 5 | 27 | 7 | 1.08 | Mishra, 2013 |
| 400 | 5 | 45 | 9 | 5.06 | Chen, 2003b |
| 400 | 5 | 47 | 9 | 4.77 | Chen, 2003b |
| 400 | 5 | not reported | 6.99 | 0.54 | Koseki, 2008 |
| 400 | 5 | not reported | 6.38 | 3.42 | Liu, 2017 |
| 400 | 5 | not reported | 6.86 | 3.93 | Liu, 2017 |
| 400 | 5 | not reported | 6.86 | 4.2 | Liu, 2017 |
| 400 | 5 | not reported | 6.86 | 4.3 | Liu, 2017 |
| 400 | 5 | not reported | 6.86 | 4.44 | Liu, 2017 |
| 400 | 5 | not reported | 6.86 | 4.44 | Liu, 2017 |
| 400 | 5 | not reported | 6.86 | 4.86 | Liu, 2017 |
| 400 | 5 | not reported | 6.86 | 4.87 | Liu, 2017 |
| 400 | 5 | not reported | 6.86 | 5.34 | Liu, 2017 |
| 400 | 5.07 | 20 | 7.26 | 4.84 | Hayman, 2007 |
| 400 | 5.25 | 20 | 6.85 | 2.95 | Hayman, 2007 |
| 400 | 5.25 | 20 | 6.85 | 3.19 | Hayman, 2007 |
| 400 | 5.25 | 20 | 7.23 | 5.31 | Hayman, 2007 |
| 400 | 6 | 8 | 6 | 3.53 | McClements, 2001 |
| 400 | 6 | 8 | 6 | 4.09 | McClements, 2001 |
| 400 | 6 | 8 | 6 | 4.7 | McClements, 2001 |
| 400 | 6 | 8 | 6 | 4.73 | McClements, 2001 |
| 400 | 6 | 22 | 7.46 | 1.95 | Wen, 2009 |
| 400 | 6 | 22 | 7.33 | 6.48 | Wen, 2009 |
| 400 | 6 | 25 | 7.69 | 2.58 | Dogan, 2004 |
| 400 | 6 | 30 | 7 | 6.65 | McClements, 2001 |
| 400 | 6 | 30 | 7 | 6.88 | McClements, 2001 |
| 400 | 6 | 30 | 7 | 6.98 | McClements, 2001 |
| 400 | 6 | 30 | 7 | 7.21 | McClements, 2001 |
| 400 | 6 | 43 | 7 | 2.34 | Mishra, 2013 |
| 400 | 6 | 50 | 9 | 6 | Chen, 2003b |
| 400 | 6.04 | 20 | 6.9 | 2.82 | Hayman, 2007 |
| 400 | 6.04 | 20 | 7.01 | 3.01 | Hayman, 2007 |
| 400 | 6.13 | 20 | 7.26 | 4.84 | Hayman, 2007 |
| 400 | 6.24 | 20 | 7.23 | 5.41 | Hayman, 2007 |
| 400 | 7.07 | 20 | 6.85 | 3.4 | Hayman, 2007 |
| 400 | 7.07 | 20 | 6.85 | 3.93 | Hayman, 2007 |
| 400 | 7.11 | 20 | 7.23 | 6.06 | Hayman, 2007 |
| 400 | 8 | 8 | 9 | 1.88 | McClements, 2001 |
| 400 | 8 | 8 | 9 | 2.89 | McClements, 2001 |
| 400 | 8 | 8 | 9 | 5.23 | McClements, 2001 |
| 400 | 8 | 8 | 9 | 6.29 | McClements, 2001 |
| 400 | 8 | 30 | 9 | 2.05 | McClements, 2001 |
| 400 | 8 | 30 | 9 | 2.18 | McClements, 2001 |
| 400 | 8 | 30 | 9 | 5.96 | McClements, 2001 |
| 400 | 8 | 30 | 9 | 5.98 | McClements, 2001 |
| 400 | 8 | 30 | 7 | 7.13 | McClements, 2001 |
| 400 | 8 | 30 | 7 | 7.21 | McClements, 2001 |
| 400 | 8 | 30 | 7 | 7.29 | McClements, 2001 |
| 400 | 8 | 50 | 9 | 6.52 | Chen, 2003b |
| 400 | 8.03 | 20 | 7.01 | 3.21 | Hayman, 2007 |
| 400 | 8.03 | 20 | 6.9 | 3.21 | Hayman, 2007 |
| 400 | 8.08 | 20 | 7.26 | 5.91 | Hayman, 2007 |
| 400 | 9 | 20 | 6.85 | 3.52 | Hayman, 2007 |
| 400 | 9 | 20 | 6.85 | 3.81 | Hayman, 2007 |
| 400 | 9 | 20 | 7.23 | 6.61 | Hayman, 2007 |
| 400 | 9 | 22 | 7.46 | 2.91 | Wen, 2009 |
| 400 | 9 | 22 | 7.33 | 6.85 | Wen, 2009 |
| 400 | 9 | 43 | 7 | 2.78 | Mishra, 2013 |
| 400 | 10 | 20 | 9 | 7.97 | Ramos, 2015 |
| 400 | 10 | 21.5 | 9 | 3.83 | Chen, 2006 |
| 400 | 10 | 22 | 9 | 0.47 | Chen, 2003b |
| 400 | 10 | 25 | 7.1 | 2.25 | Misiou, 2018 |
| 400 | 10 | 25 | 7.88 | 2.88 | Erkmen, 2004 |
| 400 | 10 | 25 | 7.1 | 3.47 | Misiou, 2018 |
| 400 | 10 | 25 | 7.1 | 3.95 | Misiou, 2018 |
| 400 | 10 | 25 | 7.1 | 4.8 | Misiou, 2018 |
| 400 | 10 | 25 | 7.4 | 4.8 | Misiou, 2018 |
| 400 | 10 | 25 | 8.7 | 7.48 | Huang, 2015 |
| 400 | 10 | 27 | 7 | 1.86 | Mishra, 2013 |
| 400 | 10 | 30 | 7 | 7.21 | McClements, 2001 |
| 400 | 10 | 40 | 9 | 3.04 | Chen, 2003b |
| 400 | 10 | 42 | 9 | 3.65 | Chen, 2003b |
| 400 | 10 | 45 | 9 | 5.45 | Chen, 2003b |
| 400 | 10 | 47 | 9 | 5.48 | Chen, 2003b |
| 400 | 10 | 50 | 9 | 6.68 | Chen, 2003b |
| 400 | 10.03 | 20 | 6.9 | 4.15 | Hayman, 2007 |
| 400 | 10.03 | 20 | 7.26 | 6.47 | Hayman, 2007 |
| 400 | 10.07 | 20 | 7.01 | 3.21 | Hayman, 2007 |
| 400 | 10.95 | 20 | 6.85 | 3.9 | Hayman, 2007 |
| 400 | 10.95 | 20 | 6.85 | 5.01 | Hayman, 2007 |
| 400 | 10.95 | 20 | 7.23 | 7.23 | Hayman, 2007 |
| 400 | 12 | 8 | 9 | 2.4 | McClements, 2001 |
| 400 | 12 | 8 | 9 | 3.42 | McClements, 2001 |
| 400 | 12 | 8 | 9 | 5.43 | McClements, 2001 |
| 400 | 12 | 8 | 9 | 6.46 | McClements, 2001 |
| 400 | 12 | 22 | 7.33 | 6.85 | Wen, 2009 |
| 400 | 12 | 30 | 9 | 2.7 | McClements, 2001 |
| 400 | 12 | 30 | 9 | 3.5 | McClements, 2001 |
| 400 | 12 | 30 | 9 | 6.63 | McClements, 2001 |
| 400 | 12 | 30 | 9 | 6.92 | McClements, 2001 |
| 400 | 12 | 43 | 7 | 3.06 | Mishra, 2013 |
| 400 | 12 | 50 | 9 | 7.24 | Chen, 2003b |
| 400 | 12.02 | 20 | 7.01 | 3.6 | Hayman, 2007 |
| 400 | 12.02 | 20 | 6.9 | 3.87 | Hayman, 2007 |
| 400 | 12.02 | 20 | 7.26 | 7.26 | Hayman, 2007 |
| 400 | 12.84 | 20 | 6.85 | 4.36 | Hayman, 2007 |
| 400 | 12.84 | 20 | 7.23 | 7.23 | Hayman, 2007 |
| 400 | 12.88 | 20 | 6.85 | 4.29 | Hayman, 2007 |
| 400 | 14 | 50 | 9 | 6.8 | Chen, 2003b |
| 400 | 15 | 20 | 9 | 7.63 | Ramos, 2015 |
| 400 | 15 | 21.5 | 9 | 0.57 | Chen, 2004 |
| 400 | 15 | 25 | 7.88 | 4.19 | Erkmen, 2004 |
| 400 | 15 | 25 | 7.69 | 4.27 | Dogan, 2004 |
| 400 | 15 | 27 | 7 | 1.98 | Mishra, 2013 |
| 400 | 15 | 43 | 7 | 3.37 | Mishra, 2013 |
| 400 | 15 | 45 | 9 | 5.63 | Chen, 2003b |
| 400 | 15 | 47 | 9 | 5.76 | Chen, 2003b |
| 400 | 16 | 8 | 9 | 5.05 | McClements, 2001 |
| 400 | 16 | 8 | 9 | 5.75 | McClements, 2001 |
| 400 | 16 | 8 | 9 | 6.15 | McClements, 2001 |
| 400 | 16 | 8 | 9 | 6.59 | McClements, 2001 |
| 400 | 16 | 20 | 6.9 | 3.74 | Hayman, 2007 |
| 400 | 16 | 30 | 9 | 3.25 | McClements, 2001 |
| 400 | 16 | 30 | 9 | 3.9 | McClements, 2001 |
| 400 | 16 | 30 | 9 | 7.15 | McClements, 2001 |
| 400 | 16 | 30 | 9 | 7.3 | McClements, 2001 |
| 400 | 16 | 50 | 9 | 7.44 | Chen, 2003b |
| 400 | 16.05 | 20 | 7.01 | 4.23 | Hayman, 2007 |
| 400 | 16.56 | 20 | 6.85 | 4.41 | Hayman, 2007 |
| 400 | 16.6 | 20 | 6.85 | 5.27 | Hayman, 2007 |
| 400 | 18 | 43 | 7 | 4.13 | Mishra, 2013 |
| 400 | 18 | 50 | 9 | 8.12 | Chen, 2003b |
| 400 | 19 | 25 | 7.69 | 5.57 | Dogan, 2004 |
| 400 | 19.99 | 20 | 6.9 | 3.77 | Hayman, 2007 |
| 400 | 20 | 8 | 9 | 5.96 | McClements, 2001 |
| 400 | 20 | 8 | 9 | 6.13 | McClements, 2001 |
| 400 | 20 | 8 | 9 | 6.75 | McClements, 2001 |
| 400 | 20 | 8 | 9 | 6.82 | McClements, 2001 |
| 400 | 20 | 20 | 9 | 3.5 | Karatzas, 2002 |
| 400 | 20 | 22 | 7 | 1.02 | Buzrul, 2009 |
| 400 | 20 | 22 | 9 | 1.98 | Chen, 2003b |
| 400 | 20 | 22 | 7.46 | 7.03 | Wen, 2009 |
| 400 | 20 | 25 | 7.88 | 4.86 | Erkmen, 2004 |
| 400 | 20 | 27 | 7 | 2.09 | Mishra, 2013 |
| 400 | 20 | 30 | 9 | 3.85 | McClements, 2001 |
| 400 | 20 | 30 | 9 | 4.51 | McClements, 2001 |
| 400 | 20 | 30 | 9 | 7.65 | McClements, 2001 |
| 400 | 20 | 30 | 9 | 8.29 | McClements, 2001 |
| 400 | 20 | 40 | 9 | 3.26 | Chen, 2003b |
| 400 | 20 | 42 | 9 | 4.31 | Chen, 2003b |
| 400 | 20 | 45 | 9 | 6.01 | Chen, 2003b |
| 400 | 20 | 47 | 9 | 6 | Chen, 2003b |
| 400 | 20 | 50 | 9 | 7.92 | Chen, 2003b |
| 400 | 20.03 | 20 | 7.01 | 4.31 | Hayman, 2007 |
| 400 | 20.39 | 20 | 6.85 | 4.93 | Hayman, 2007 |
| 400 | 20.43 | 20 | 6.85 | 6.75 | Hayman, 2007 |
| 400 | 24 | 8 | 9 | 6.65 | McClements, 2001 |
| 400 | 24 | 8 | 9 | 6.92 | McClements, 2001 |
| 400 | 24 | 8 | 9 | 7.23 | McClements, 2001 |
| 400 | 24 | 8 | 9 | 7.4 | McClements, 2001 |
| 400 | 24 | 30 | 9 | 4.25 | McClements, 2001 |
| 400 | 24 | 30 | 9 | 4.82 | McClements, 2001 |
| 400 | 24 | 30 | 9 | 8.3 | McClements, 2001 |
| 400 | 24 | 30 | 9 | 8.44 | McClements, 2001 |
| 400 | 24.99 | 20 | 6.9 | 4.15 | Hayman, 2007 |
| 400 | 25 | 27 | 7 | 2.21 | Mishra, 2013 |
| 400 | 25 | 45 | 9 | 6.23 | Chen, 2003b |
| 400 | 25 | 47 | 9 | 6.46 | Chen, 2003b |
| 400 | 25.08 | 20 | 7.01 | 4.67 | Hayman, 2007 |
| 400 | 25.18 | 20 | 6.85 | 5.03 | Hayman, 2007 |
| 400 | 25.18 | 20 | 6.85 | 6.85 | Hayman, 2007 |
| 400 | 30 | 21.5 | 9 | 3.54 | Chen, 2004 |
| 400 | 30 | 22 | 9 | 3.4 | Chen, 2003b |
| 400 | 30 | 25 | 7.88 | 6.43 | Erkmen, 2004 |
| 400 | 30 | 25 | 7.69 | 6.87 | Dogan, 2004 |
| 400 | 30 | 27 | 7 | 2.59 | Mishra, 2013 |
| 400 | 30 | 40 | 9 | 3.99 | Chen, 2003b |
| 400 | 30 | 42 | 9 | 5.53 | Chen, 2003b |
| 400 | 30 | 45 | 9 | 6.27 | Chen, 2003b |
| 400 | 35 | 45 | 9 | 6.45 | Chen, 2003b |
| 400 | 40 | 22 | 9 | 4.65 | Chen, 2003b |
| 400 | 40 | 25 | 7.88 | 7.42 | Erkmen, 2004 |
| 400 | 40 | 25 | 7.69 | 7.57 | Dogan, 2004 |
| 400 | 40 | 40 | 9 | 5.31 | Chen, 2003b |
| 400 | 40 | 42 | 9 | 6 | Chen, 2003b |
| 400 | 40 | 45 | 9 | 6.66 | Chen, 2003b |
| 400 | 45 | 21.5 | 9 | 4.37 | Chen, 2004 |
| 400 | 45 | 25 | 7.69 | 7.67 | Dogan, 2004 |
| 400 | 45 | 45 | 9 | 7.53 | Chen, 2003b |
| 400 | 50 | 22 | 9 | 5.6 | Chen, 2003b |
| 400 | 50 | 25 | 7.88 | 7.88 | Erkmen, 2004 |
| 400 | 50 | 40 | 9 | 5.7 | Chen, 2003b |
| 400 | 50 | 42 | 9 | 6.93 | Chen, 2003b |
| 400 | 50 | 45 | 9 | 8.87 | Chen, 2003b |
| 400 | 60 | 21.5 | 9 | 5.59 | Chen, 2004 |
| 400 | 60 | 22 | 9 | 5.81 | Chen, 2003b |
| 400 | 60 | 40 | 9 | 5.83 | Chen, 2003b |
| 400 | 70 | 22 | 9 | 6.33 | Chen, 2003b |
| 400 | 70 | 25 | 9 | 3.5 | Dogan, 2004 |
| 400 | 70 | 40 | 9 | 6.09 | Chen, 2003b |
| 400 | 75 | 21.5 | 9 | 5.77 | Chen, 2004 |
| 400 | 80 | 22 | 9 | 6.72 | Chen, 2003b |
| 400 | 80 | 40 | 9 | 6.73 | Chen, 2003b |
| 400 | 90 | 21.5 | 9 | 6.12 | Chen, 2004 |
| 400 | 90 | 22 | 9 | 6.67 | Chen, 2003b |
| 400 | 90 | 40 | 9 | 7.29 | Chen, 2003b |
| 400 | 100 | 22 | 9 | 6.89 | Chen, 2003b |
| 400 | 100 | 40 | 9 | 8.27 | Chen, 2003b |
| 400 | 105 | 21.5 | 9 | 6.16 | Chen, 2004 |
| 400 | 120 | 21.5 | 9 | 6.29 | Chen, 2004 |
| 400 | 120 | 22 | 9 | 6.93 | Chen, 2003b |
| 400 | 135 | 21.5 | 9 | 6.69 | Chen, 2004 |
| 400 | 150 | 21.5 | 9 | 6.95 | Chen, 2004 |
| 400 | 180 | 21.5 | 9 | 8.48 | Chen, 2004 |
| 430 | 10 | 21.5 | 9 | 2.84 | Chen, 2004 |
| 430 | 20 | 21.5 | 9 | 4.57 | Chen, 2004 |
| 430 | 30 | 21.5 | 9 | 4.96 | Chen, 2004 |
| 430 | 40 | 21.5 | 9 | 5.31 | Chen, 2004 |
| 430 | 50 | 21.5 | 9 | 6.39 | Chen, 2004 |
| 430 | 60 | 21.5 | 9 | 6.54 | Chen, 2004 |
| 430 | 70 | 21.5 | 9 | 7.62 | Chen, 2004 |
| 430 | 80 | 21.5 | 9 | 7.57 | Chen, 2004 |
| 430 | 90 | 21.5 | 9 | 7.97 | Chen, 2004 |
| 430 | 100 | 21.5 | 9 | 8.61 | Chen, 2004 |
| 450 | 5 | 21.5 | 9 | 6.76 | Chen, 2006 |
| 450 | 5 | 25 | 8.7 | 8.624 | Huang, 2015 |
| 450 | 5 | N/A | 6.99 | 1.61 | Koseki, 2008 |
| 450 | 10 | 21.5 | 9 | 6.93 | Chen, 2006 |
| 450 | 10 | 25 | 8.7 | 8.62 | Huang, 2015 |
| 500 | 0.5 | 50 | 9 | 4.81 | Chen, 2003b |
| 500 | 1 | 18 | 8 | 1.7 | Stratakos, 2019 |
| 500 | 1 | 27 | 7 | 1.25 | Mishra, 2013 |
| 500 | 1 | 45 | 9 | 4.73 | Chen, 2003b |
| 500 | 1 | 47 | 9 | 4.74 | Chen, 2003b |
| 500 | 1 | 50 | 9 | 6.52 | Chen, 2003b |
| 500 | 1.5 | 50 | 9 | 6.63 | Chen, 2003b |
| 500 | 2 | 21 | 7 | 1.29 | Shearer, 2010 |
| 500 | 2 | 21 | 7 | 1.41 | Shearer, 2010 |
| 500 | 2 | 21 | 7 | 1.78 | Shearer, 2010 |
| 500 | 2 | 21 | 7 | 2.11 | Shearer, 2010 |
| 500 | 2 | 21 | 7 | 2.74 | Shearer, 2010 |
| 500 | 2 | 21 | 7 | 3.02 | Shearer, 2010 |
| 500 | 2 | 21 | 7 | 3.43 | Shearer, 2010 |
| 500 | 2 | 21 | 7 | 3.73 | Shearer, 2010 |
| 500 | 2 | 27 | 7 | 1.39 | Mishra, 2013 |
| 500 | 2 | 42 | 9 | 4.71 | Chen, 2003b |
| 500 | 2 | 45 | 9 | 5.03 | Chen, 2003b |
| 500 | 2 | 47 | 9 | 5.43 | Chen, 2003b |
| 500 | 2 | 50 | 9 | 7.18 | Chen, 2003b |
| 500 | 2.5 | 22 | 9 | 2.12 | Chen, 2003b |
| 500 | 2.5 | 40 | 9 | 4.95 | Chen, 2003b |
| 500 | 2.5 | 50 | 9 | 7.14 | Chen, 2003b |
| 500 | 3 | 4.4 | 7.08 | 1.25 | Kabir, 2021 |
| 500 | 3 | 4.4 | 7.08 | 1.83 | Kabir, 2021 |
| 500 | 3 | 18 | 8 | 3.35 | Stratakos, 2019 |
| 500 | 3 | 27 | 7 | 1.93 | Mishra, 2013 |
| 500 | 3 | 45 | 9 | 5.48 | Chen, 2003b |
| 500 | 3 | 47 | 9 | 6.05 | Chen, 2003b |
| 500 | 3 | 50 | 9 | 7.23 | Chen, 2003b |
| 500 | 3 | 60 | 6.9 | 4.81 | Kabir, 2021 |
| 500 | 3 | 60 | 6.9 | 5.21 | Kabir, 2021 |
| 500 | 3.5 | 50 | 9 | 6.94 | Chen, 2003b |
| 500 | 4 | 21.5 | 9 | 1.79 | Chen, 2004 |
| 500 | 4 | 27 | 7 | 3.14 | Mishra, 2013 |
| 500 | 4 | 42 | 9 | 5.62 | Chen, 2003b |
| 500 | 4 | 45 | 9 | 5.79 | Chen, 2003b |
| 500 | 4 | 47 | 9 | 6.14 | Chen, 2003b |
| 500 | 4 | 50 | 9 | 7.68 | Chen, 2003b |
| 500 | 4.5 | 50 | 9 | 7.56 | Chen, 2003b |
| 500 | 5 | 18 | 8 | 5 | Stratakos, 2019 |
| 500 | 5 | 20 | 9 | 7.41 | Ramos, 2015 |
| 500 | 5 | 21.5 | 9 | 7.54 | Chen, 2006 |
| 500 | 5 | 22 | 9 | 3.04 | Chen, 2003b |
| 500 | 5 | 27 | 7 | 3.88 | Mishra, 2013 |
| 500 | 5 | 40 | 9 | 5.75 | Chen, 2003b |
| 500 | 5 | 45 | 9 | 6.69 | Chen, 2003b |
| 500 | 5 | 47 | 9 | 6.62 | Chen, 2003b |
| 500 | 5 | 50 | 9 | 8.12 | Chen, 2003b |
| 500 | 5 | not reported | 6.99 | 5.18 | Koseki, 2008 |
| 500 | 5 | not reported | 6.38 | 6.38 | Liu, 2017 |
| 500 | 6 | 27 | 7 | 5.07 | Mishra, 2013 |
| 500 | 6 | 42 | 9 | 5.81 | Chen, 2003b |
| 500 | 6 | 43 | 7 | 2.62 | Mishra, 2013 |
| 500 | 6 | 43 | 7 | 3.41 | Mishra, 2013 |
| 500 | 6 | 43 | 7 | 3.99 | Mishra, 2013 |
| 500 | 6 | 43 | 7 | 4.53 | Mishra, 2013 |
| 500 | 6 | 43 | 7 | 5.53 | Mishra, 2013 |
| 500 | 6 | 43 | 7 | 6.04 | Mishra, 2013 |
| 500 | 6 | 45 | 9 | 6.58 | Chen, 2003b |
| 500 | 7 | 45 | 9 | 6.84 | Chen, 2003b |
| 500 | 7.5 | 40 | 9 | 5.71 | Chen, 2003b |
| 500 | 8 | 21.5 | 9 | 5.04 | Chen, 2004 |
| 500 | 8 | 42 | 9 | 7.48 | Chen, 2003b |
| 500 | 8 | 45 | 9 | 7.75 | Chen, 2003b |
| 500 | 9 | 45 | 9 | 8.28 | Chen, 2003b |
| 500 | 10 | 20 | 9 | 7.97 | Ramos, 2015 |
| 500 | 10 | 21.5 | 9 | 7 | Chen, 2006 |
| 500 | 10 | 22 | 9 | 5.92 | Chen, 2003b |
| 500 | 10 | 25 | 7.4 | 6.22 | Misiou, 2018 |
| 500 | 10 | 40 | 9 | 6.01 | Chen, 2003b |
| 500 | 10 | 42 | 9 | 7.95 | Chen, 2003b |
| 500 | 10 | 45 | 9 | 8.7 | Chen, 2003b |
| 500 | 12 | 21.5 | 9 | 5.56 | Chen, 2004 |
| 500 | 12.5 | 22 | 9 | 6 | Chen, 2003b |
| 500 | 12.5 | 40 | 9 | 6.58 | Chen, 2003b |
| 500 | 15 | 20 | 9 | 7.97 | Ramos, 2015 |
| 500 | 15 | 22 | 9 | 6.56 | Chen, 2003b |
| 500 | 15 | 40 | 9 | 6.5 | Chen, 2003b |
| 500 | 16 | 21.5 | 9 | 5.7 | Chen, 2004 |
| 500 | 17.5 | 22 | 9 | 6.64 | Chen, 2003b |
| 500 | 17.5 | 40 | 9 | 7.3 | Chen, 2003b |
| 500 | 20 | 21.5 | 9 | 6.17 | Chen, 2004 |
| 500 | 20 | 22 | 9 | 7.04 | Chen, 2003b |
| 500 | 20 | 40 | 9 | 8.36 | Chen, 2003b |
| 500 | 22.5 | 22 | 9 | 7.44 | Chen, 2003b |
| 500 | 22.5 | 40 | 9 | 8.43 | Chen, 2003b |
| 500 | 25 | 21.5 | 9 | 6.5 | Chen, 2004 |
| 500 | 25 | 22 | 9 | 7.64 | Chen, 2003b |
| 500 | 25 | 40 | 9 | 8.66 | Chen, 2003b |
| 500 | 28 | 21.5 | 9 | 6.79 | Chen, 2004 |
| 500 | 32 | 21.5 | 9 | 7.49 | Chen, 2004 |
| 500 | 35 | 22 | 9 | 8 | Chen, 2003b |
| 500 | 36 | 21.5 | 9 | 8.01 | Chen, 2004 |
| 500 | 40 | 21.5 | 9 | 8.34 | Chen, 2004 |
| 530 | 2 | 21.5 | 9 | 2.64 | Chen, 2004 |
| 530 | 4 | 21.5 | 9 | 4.22 | Chen, 2004 |
| 530 | 6 | 21.5 | 9 | 4.98 | Chen, 2004 |
| 530 | 8 | 21.5 | 9 | 5.27 | Chen, 2004 |
| 530 | 10 | 21.5 | 9 | 5.51 | Chen, 2004 |
| 530 | 12 | 21.5 | 9 | 6.13 | Chen, 2004 |
| 530 | 14 | 21.5 | 9 | 7.33 | Chen, 2004 |
| 530 | 16 | 21.5 | 9 | 8.33 | Chen, 2004 |
| 530 | 18 | 21.5 | 9 | 7.85 | Chen, 2004 |
| 530 | 20 | 21.5 | 9 | 8.33 | Chen, 2004 |
| 550 | 5 | 21.5 | 9 | 7.69 | Chen, 2006 |
| 550 | 10 | 21.5 | 9 | 8 | Chen, 2006 |
| 600 | 0.33 | 27 | 7 | 1.77 | Mishra, 2013 |
| 600 | 0.33 | 43 | 7 | 2.12 | Mishra, 2013 |
| 600 | 0.5 | 21.5 | 9 | 1.43 | Chen, 2004 |
| 600 | 0.5 | 21.5 | 9 | 6.64 | Chen, 2007 |
| 600 | 0.66 | 27 | 7 | 2.34 | Mishra, 2013 |
| 600 | 0.66 | 43 | 7 | 4.27 | Mishra, 2013 |
| 600 | 1 | 18 | 8 | 3.35 | Stratakos, 2019 |
| 600 | 1 | 21.5 | 9 | 3.16 | Chen, 2004 |
| 600 | 1 | 21.5 | 9 | 7.16 | Chen, 2007 |
| 600 | 1 | 25 | 7.88 | 1.26 | Erkmen, 2004 |
| 600 | 1 | 25 | 7.69 | 1.98 | Dogan, 2004 |
| 600 | 1 | 27 | 7 | 3.26 | Mishra, 2013 |
| 600 | 1 | 43 | 7 | 4.62 | Mishra, 2013 |
| 600 | 1.33 | 27 | 7 | 5.09 | Mishra, 2013 |
| 600 | 1.33 | 43 | 7 | 6.32 | Mishra, 2013 |
| 600 | 1.5 | 21.5 | 9 | 3.39 | Chen, 2004 |
| 600 | 1.5 | 21.5 | 9 | 7 | Chen, 2007 |
| 600 | 1.66 | 27 | 7 | 5.34 | Mishra, 2013 |
| 600 | 2 | 21.5 | 9 | 3.79 | Chen, 2004 |
| 600 | 2 | 21.5 | 9 | 7.48 | Chen, 2007 |
| 600 | 2 | 27 | 7 | 5.38 | Mishra, 2013 |
| 600 | 2.5 | 21.5 | 9 | 4.37 | Chen, 2004 |
| 600 | 2.5 | 21.5 | 9 | 7.16 | Chen, 2007 |
| 600 | 3 | 18 | 8 | 5.22 | Stratakos, 2019 |
| 600 | 3 | 21.5 | 9 | 4.54 | Chen, 2004 |
| 600 | 3 | 25 | 7.69 | 3.64 | Dogan, 2004 |
| 600 | 3 | 25 | 7.88 | 3.87 | Erkmen, 2004 |
| 600 | 3.5 | 21.5 | 9 | 4.86 | Chen, 2004 |
| 600 | 4 | 21.5 | 9 | 5.12 | Chen, 2004 |
| 600 | 4 | 21.5 | 9 | 7.73 | Chen, 2007 |
| 600 | 4.5 | 21.5 | 9 | 5.44 | Chen, 2004 |
| 600 | 5 | 18 | 8 | 5.39 | Stratakos, 2019 |
| 600 | 5 | 20 | 9 | 7.84 | Ramos, 2015 |
| 600 | 5 | 21.5 | 9 | 5.75 | Chen, 2004 |
| 600 | 5 | 21.5 | 9 | 8.22 | Chen, 2006 |
| 600 | 5 | 25 | 7.88 | 5.55 | Erkmen, 2004 |
| 600 | 6 | 21.5 | 9 | 6.33 | Chen, 2004 |
| 600 | 6 | 21.5 | 9 | 7.6 | Chen, 2007 |
| 600 | 6 | 25 | 7.69 | 5.45 | Dogan, 2004 |
| 600 | 7 | 21.5 | 9 | 6.82 | Chen, 2004 |
| 600 | 8 | 21.5 | 9 | 7.35 | Chen, 2004 |
| 600 | 9 | 21.5 | 9 | 7.75 | Chen, 2004 |
| 600 | 10 | 20 | 9 | 7.84 | Ramos, 2015 |
| 600 | 10 | 21.5 | 9 | 7.71 | Chen, 2004 |
| 600 | 10 | 21.5 | 9 | 8.93 | Chen, 2006 |
| 600 | 10 | 25 | 7.88 | 6.68 | Erkmen, 2004 |
| 600 | 10 | 25 | 7.69 | 6.73 | Dogan, 2004 |
| 600 | 15 | 20 | 9 | 7.84 | Ramos, 2015 |
| 600 | 15 | 25 | 7.88 | 7.3 | Erkmen, 2004 |
| 600 | 17 | 25 | 7.69 | 7.67 | Dogan, 2004 |
| 600 | 20 | 25 | 7.88 | 7.88 | Erkmen, 2004 |
| 650 | 5 | 21.5 | 9 | 8.85 | Chen, 2006 |
| 650 | 10 | 21.5 | 9 | 8.33 | Chen, 2006 |
| ***Pseudomonas aeruginosa*** | | | | | |
| 200 | 5 | 20 | 9 | 0.12 | Ramos, 2015 |
| 200 | 10 | 20 | 9 | 0.34 | Ramos, 2015 |
| 200 | 15 | 20 | 9 | 0.07 | Ramos, 2015 |
| 300 | 5 | 20 | 9 | 2.3 | Ramos, 2015 |
| 300 | 10 | 20 | 9 | 7.92 | Ramos, 2015 |
| 300 | 15 | 20 | 9 | 7.9 | Ramos, 2015 |
| 400 | 5 | 20 | 9 | 7.68 | Ramos, 2015 |
| 400 | 10 | 20 | 9 | 7.68 | Ramos, 2015 |
| 400 | 15 | 20 | 9 | 7.68 | Ramos, 2015 |
| 500 | 5 | 20 | 9 | 7.66 | Ramos, 2015 |
| 500 | 10 | 20 | 9 | 7.66 | Ramos, 2015 |
| 500 | 15 | 20 | 9 | 7.66 | Ramos, 2015 |
| 600 | 5 | 20 | 9 | 7.66 | Ramos, 2015 |
| 600 | 10 | 20 | 9 | 7.66 | Ramos, 2015 |
| 600 | 15 | 20 | 9 | 7.66 | Ramos, 2015 |
| ***Salmonella enterica*** | | | | | |
| 200 | 5 | not reported | 6.64 | 0.58 | Liu, 2017 |
| 200 | 10 | 21.5 | 9 | 0 | Chen, 2006 |
| 200 | 10 | 21.5 | 9 | 0.14 | Chen, 2006 |
| 225 | 15 | 25 | 7 | 0.44 | Nakimbugwe, 2006 |
| 225 | 15 | 25 | 7 | 0.55 | Nakimbugwe, 2006 |
| 225 | 15 | 25 | 7 | 1.95 | Nakimbugwe, 2006 |
| 250 | 10 | 21.5 | 9 | 0.1 | Chen, 2006 |
| 250 | 10 | 21.5 | 9 | 0.21 | Chen, 2006 |
| 300 | 5 | not reported | 6.5 | 2.66 | Liu, 2017 |
| 300 | 5 | not reported | 6.64 | 2.82 | Liu, 2017 |
| 300 | 5 | not reported | 6.5 | 2.9 | Liu, 2017 |
| 300 | 5 | not reported | 6.5 | 3.39 | Liu, 2017 |
| 300 | 5 | not reported | 6.5 | 3.4 | Liu, 2017 |
| 300 | 5 | not reported | 6.5 | 3.41 | Liu, 2017 |
| 300 | 5 | not reported | 6.5 | 3.93 | Liu, 2017 |
| 300 | 5 | not reported | 6.5 | 4.15 | Liu, 2017 |
| 300 | 5 | not reported | 6.5 | 4.29 | Liu, 2017 |
| 300 | 10 | 21.5 | 9 | 0.02 | Chen, 2006 |
| 300 | 10 | 21.5 | 9 | 0.43 | Chen, 2006 |
| 300 | 30 | 20 | 7.33 | 6.16 | Foster, 2016 |
| 300 | 45 | 20 | 7.33 | 6.85 | Foster, 2016 |
| 300 | 60 | 20 | 7.33 | 5.17 | Foster, 2016 |
| 350 | 10 | 20 | 9 | 0.21 | Guan, 2005 |
| 350 | 10 | 21.5 | 9 | 0.19 | Chen, 2006 |
| 350 | 10 | 21.5 | 9 | 0.62 | Chen, 2006 |
| 350 | 20 | 20 | 9 | 0.4 | Guan, 2005 |
| 350 | 30 | 20 | 9 | 0.61 | Guan, 2005 |
| 350 | 60 | 20 | 9 | 1.2 | Guan, 2005 |
| 350 | 90 | 20 | 9 | 2.51 | Guan, 2005 |
| 350 | 120 | 20 | 9 | 3.11 | Guan, 2005 |
| 400 | 1 | 18 | 8 | 1.1 | Stratakos, 2019 |
| 400 | 3 | 18 | 8 | 1.36 | Stratakos, 2019 |
| 400 | 5 | 18 | 8 | 2.02 | Stratakos, 2019 |
| 400 | 5 | 25 | 7.34 | 2.85 | Erkmen, 2011 |
| 400 | 5 | not reported | 6.64 | 6.64 | Liu, 2017 |
| 400 | 10 | 20 | 7.33 | 6.55 | Foster, 2016 |
| 400 | 10 | 20 | 9 | 1.04 | Guan, 2005 |
| 400 | 10 | 21.5 | 9 | 0.75 | Chen, 2006 |
| 400 | 10 | 21.5 | 9 | 1.46 | Chen, 2006 |
| 400 | 10 | 25 | 7.34 | 3.87 | Erkmen, 2011 |
| 400 | 15 | 20 | 7.33 | 6.77 | Foster, 2016 |
| 400 | 15 | 25 | 7.34 | 4.46 | Erkmen, 2011 |
| 400 | 20 | 20 | 7.33 | 7.14 | Foster, 2016 |
| 400 | 20 | 20 | 9 | 1.36 | Guan, 2005 |
| 400 | 20 | 25 | 7.34 | 5.3 | Erkmen, 2011 |
| 400 | 25 | 25 | 7.34 | 5.65 | Erkmen, 2011 |
| 400 | 30 | 20 | 9 | 1.79 | Guan, 2005 |
| 400 | 30 | 25 | 7.34 | 6.13 | Erkmen, 2011 |
| 400 | 33 | 20 | 9 | 2.98 | Guan, 2005 |
| 400 | 40 | 25 | 7.34 | 6.78 | Erkmen, 2011 |
| 400 | 45 | 25 | 7.34 | 7.32 | Erkmen, 2011 |
| 400 | 60 | 20 | 9 | 4.48 | Guan, 2005 |
| 450 | 10 | 20 | 9 | 2.38 | Guan, 2005 |
| 450 | 10 | 21.5 | 9 | 1.74 | Chen, 2006 |
| 450 | 10 | 21.5 | 9 | 3.1 | Chen, 2006 |
| 450 | 14 | 20 | 9 | 3.09 | Guan, 2005 |
| 450 | 22 | 20 | 9 | 4.58 | Guan, 2005 |
| 450 | 30 | 20 | 9 | 5 | Guan, 2005 |
| 500 | 1 | 18 | 8 | 1.71 | Stratakos, 2019 |
| 500 | 2 | 20 | 9 | 0.46 | Guan, 2005 |
| 500 | 3 | 18 | 8 | 2.07 | Stratakos, 2019 |
| 500 | 5 | 18 | 8 | 3.3 | Stratakos, 2019 |
| 500 | 5 | not reported | 6.64 | 6.64 | Liu, 2017 |
| 500 | 8 | 20 | 9 | 4.21 | Guan, 2005 |
| 500 | 10 | 20 | 9 | 4.55 | Guan, 2005 |
| 500 | 10 | 21.5 | 9 | 3.4 | Chen, 2006 |
| 500 | 10 | 21.5 | 9 | 5.28 | Chen, 2006 |
| 500 | 20 | 20 | 9 | 4.92 | Guan, 2005 |
| 500 | 30 | 20 | 9 | 5.02 | Guan, 2005 |
| 500 | 40 | 20 | 9 | 5.6 | Guan, 2005 |
| 500 | 50 | 20 | 9 | 7.25 | Guan, 2005 |
| 550 | 2 | 20 | 9 | 2.47 | Guan, 2005 |
| 550 | 8 | 20 | 9 | 4.61 | Guan, 2005 |
| 550 | 10 | 20 | 9 | 4.85 | Guan, 2005 |
| 550 | 10 | 21.5 | 9 | 5.62 | Chen, 2006 |
| 550 | 10 | 21.5 | 9 | 6.59 | Chen, 2006 |
| 550 | 20 | 20 | 9 | 5.11 | Guan, 2005 |
| 550 | 30 | 20 | 9 | 5.39 | Guan, 2005 |
| 550 | 40 | 20 | 9 | 6.8 | Guan, 2005 |
| 550 | 50 | 20 | 9 | 7.78 | Guan, 2005 |
| 600 | 1 | 18 | 8 | 2.51 | Stratakos, 2019 |
| 600 | 2 | 20 | 9 | 4 | Guan, 2005 |
| 600 | 2 | 21.5 | 9 | 4.6 | Chen, 2007 |
| 600 | 2 | 21.5 | 9 | 4.66 | Chen, 2007 |
| 600 | 2 | 21.5 | 9 | 3.65 | Chen, 2007 |
| 600 | 3 | 18 | 8 | 5.01 | Stratakos, 2019 |
| 600 | 4 | 21.5 | 9 | 5.43 | Chen, 2007 |
| 600 | 4 | 21.5 | 9 | 5.46 | Chen, 2007 |
| 600 | 4 | 21.5 | 9 | 5.59 | Chen, 2007 |
| 600 | 5 | 18 | 8 | 6.29 | Stratakos, 2019 |
| 600 | 6 | 21.5 | 9 | 5.83 | Chen, 2007 |
| 600 | 6 | 21.5 | 9 | 6.7 | Chen, 2007 |
| 600 | 6 | 21.5 | 9 | 6.76 | Chen, 2007 |
| 600 | 8 | 20 | 9 | 4.89 | Guan, 2005 |
| 600 | 8 | 21.5 | 9 | 6.45 | Chen, 2007 |
| 600 | 8 | 21.5 | 9 | 6.52 | Chen, 2007 |
| 600 | 8 | 21.5 | 9 | 6.68 | Chen, 2007 |
| 600 | 10 | 20 | 9 | 5.15 | Guan, 2005 |
| 600 | 10 | 21.5 | 9 | 6.51 | Chen, 2006 |
| 600 | 10 | 21.5 | 9 | 6.86 | Chen, 2007 |
| 600 | 10 | 21.5 | 9 | 7 | Chen, 2007 |
| 600 | 10 | 21.5 | 9 | 7.77 | Chen, 2007 |
| 600 | 10 | 21.5 | 9 | 8.19 | Chen, 2006 |
| 600 | 15 | 21.5 | 9 | 7.63 | Chen, 2007 |
| 600 | 15 | 21.5 | 9 | 7.69 | Chen, 2007 |
| 600 | 15 | 21.5 | 9 | 7.77 | Chen, 2007 |
| 600 | 20 | 20 | 9 | 6.38 | Guan, 2005 |
| 600 | 20 | 21.5 | 9 | 6.77 | Chen, 2007 |
| 600 | 20 | 21.5 | 9 | 6.92 | Chen, 2007 |
| 600 | 20 | 21.5 | 9 | 7.24 | Chen, 2007 |
| 600 | 30 | 20 | 9 | 8.91 | Guan, 2005 |
| 650 | 10 | 21.5 | 9 | 7.26 | Chen, 2006 |
| 650 | 10 | 21.5 | 9 | 8.16 | Chen, 2006 |
| 690 | 10 | 21.5 | 9 | 8.13 | Chen, 2006 |
| 690 | 10 | 21.5 | 9 | 8.5 | Chen, 2006 |
| ***Shigella flexneri*** | | | | | |
| 200 | 10 | 21.5 | 9 | 0 | Chen, 2006 |
| 200 | 10 | 50 | 9 | 0.05 | Chen, 2006 |
| 200 | 10 | 50 | 9 | 0.28 | Chen, 2006 |
| 250 | 10 | 21.5 | 9 | 0.05 | Chen, 2006 |
| 250 | 10 | 50 | 9 | 0.69 | Chen, 2006 |
| 275 | 15 | 25 | 7 | 2.17 | Nakimbugwe, 2006 |
| 275 | 15 | 25 | 7 | 2.29 | Nakimbugwe, 2006 |
| 275 | 15 | 25 | 7 | 4.41 | Nakimbugwe, 2006 |
| 300 | 10 | 21.5 | 9 | 0.12 | Chen, 2006 |
| 300 | 10 | 50 | 9 | 1.23 | Chen, 2006 |
| 350 | 10 | 21.5 | 9 | 0.14 | Chen, 2006 |
| 350 | 10 | 50 | 9 | 2.06 | Chen, 2006 |
| 400 | 10 | 21.5 | 9 | 0.17 | Chen, 2006 |
| 400 | 10 | 50 | 9 | 4.28 | Chen, 2006 |
| 450 | 10 | 21.5 | 9 | 0.45 | Chen, 2006 |
| 450 | 10 | 50 | 9 | 4.56 | Chen, 2006 |
| 500 | 10 | 21.5 | 9 | 0.88 | Chen, 2006 |
| 500 | 10 | 50 | 9 | 5.88 | Chen, 2006 |
| 550 | 10 | 21.5 | 9 | 1.19 | Chen, 2006 |
| 550 | 10 | 50 | 9 | 6.4 | Chen, 2006 |
| 600 | 1 | 50 | 9 | 5.52 | Chen, 2007 |
| 600 | 2 | 50 | 9 | 5.92 | Chen, 2007 |
| 600 | 3 | 50 | 9 | 6.16 | Chen, 2007 |
| 600 | 4 | 50 | 9 | 6.29 | Chen, 2007 |
| 600 | 6 | 50 | 9 | 7.06 | Chen, 2007 |
| 600 | 8 | 50 | 9 | 6.62 | Chen, 2007 |
| 600 | 10 | 21.5 | 9 | 1.69 | Chen, 2006 |
| 600 | 10 | 50 | 9 | 7.2 | Chen, 2006 |
| 600 | 10 | 50 | 9 | 7.53 | Chen, 2007 |
| 650 | 10 | 21.5 | 9 | 2.15 | Chen, 2006 |
| ***Staphylococcus aureus*** | | | | | |
| 50 | 4 | 20 | 8.58 | N/A | Erkmen, 1997 |
| 100 | 15 | 10 | 9 | 0 | Patterson, 1998 |
| 100 | 15 | 10 | 9 | 0.03 | Patterson, 1998 |
| 100 | 15 | 20 | 9 | 0.28 | Patterson, 1998 |
| 100 | 15 | 20 | 9 | 0.59 | Patterson, 1998 |
| 100 | 15 | 40 | 9 | 0 | Patterson, 1998 |
| 100 | 15 | 40 | 9 | 0 | Patterson, 1998 |
| 100 | 15 | 55 | 9 | 0.34 | Patterson, 1998 |
| 100 | 15 | 60 | 9 | 1.48 | Patterson, 1998 |
| 100 | 15 | 60 | 9 | 2.18 | Patterson, 1998 |
| 200 | 5 | 20 | 9 | 0.12 | Ramos, 2015 |
| 200 | 5 | not reported | 6.54 | 0.02 | Liu, 2017 |
| 200 | 10 | 20 | 9 | 0.14 | Ramos, 2015 |
| 200 | 10 | 21.5 | 9 | 0.05 | Chen, 2006 |
| 200 | 10 | 30 | 9 | 3.21 | Gao, 2005 |
| 200 | 10 | 30 | 9 | 3.21 | Gao, 2005 |
| 200 | 10 | 50 | 9 | 0.12 | Chen, 2006 |
| 200 | 15 | 10 | 9 | 0 | Patterson, 1998 |
| 200 | 15 | 10 | 9 | 0.03 | Patterson, 1998 |
| 200 | 15 | 20 | 9 | 0.07 | Ramos, 2015 |
| 200 | 15 | 20 | 9 | 0.1 | Patterson, 1998 |
| 200 | 15 | 20 | 9 | 0.2 | Patterson, 1998 |
| 200 | 15 | 20 | 9 | 2.91 | Gao, 2005 |
| 200 | 15 | 40 | 9 | 0 | Patterson, 1998 |
| 200 | 15 | 40 | 9 | 0 | Patterson, 1998 |
| 200 | 15 | 40 | 9 | 4.53 | Gao, 2005 |
| 200 | 15 | 50 | 9 | 0.42 | Patterson, 1998 |
| 200 | 15 | 50 | 9 | 0.8 | Patterson, 1998 |
| 200 | 15 | 55 | 9 | 2.41 | Patterson, 1998 |
| 200 | 15 | 55 | 9 | 3 | Patterson, 1998 |
| 200 | 15 | 60 | 9 | 6.65 | Patterson, 1998 |
| 200 | 15 | 60 | 9 | 7.7 | Patterson, 1998 |
| 200 | 16 | 30 | 9 | 4.15 | Gao, 2005 |
| 200 | 20 | 30 | 9 | 4.06 | Gao, 2005 |
| 250 | 10 | 21.5 | 9 | 0 | Chen, 2006 |
| 250 | 10 | 50 | 9 | 0.28 | Chen, 2006 |
| 250 | 11 | 35 | 9 | 3.61 | Gao, 2005 |
| 250 | 14 | 25 | 9 | 4.1 | Gao, 2005 |
| 260 | 15 | 35 | 9 | 5.1 | Gao, 2005 |
| 300 | 5 | 20 | 9 | 0.63 | Ramos, 2015 |
| 300 | 5 | not reported | 6.19 | 0.09 | Liu, 2017 |
| 300 | 5 | not reported | 6.19 | 0.09 | Liu, 2017 |
| 300 | 5 | not reported | 6.19 | 0.13 | Liu, 2017 |
| 300 | 5 | not reported | 6.19 | 0.13 | Liu, 2017 |
| 300 | 5 | not reported | 6.19 | 0.14 | Liu, 2017 |
| 300 | 5 | not reported | 6.54 | 0.46 | Liu, 2017 |
| 300 | 5 | not reported | 6.19 | 0.69 | Liu, 2017 |
| 300 | 5 | not reported | 6.19 | 0.69 | Liu, 2017 |
| 300 | 5 | not reported | 6.19 | 0.7 | Liu, 2017 |
| 300 | 10 | 20 | 9 | 1.65 | Ramos, 2015 |
| 300 | 10 | 20 | 9 | 2.97 | Gao, 2005 |
| 300 | 10 | 21.5 | 9 | 0.1 | Chen, 2006 |
| 300 | 10 | 40 | 9 | 4.42 | Gao, 2005 |
| 300 | 10 | 50 | 9 | 0.99 | Chen, 2006 |
| 300 | 12 | 25 | 9 | 4.19 | Gao, 2005 |
| 300 | 15 | 10 | 9 | 0.15 | Patterson, 1998 |
| 300 | 15 | 10 | 9 | 0.17 | Patterson, 1998 |
| 300 | 15 | 20 | 9 | 0.61 | Patterson, 1998 |
| 300 | 15 | 20 | 9 | 0.93 | Patterson, 1998 |
| 300 | 15 | 20 | 9 | 1.76 | Ramos, 2015 |
| 300 | 15 | 30 | 9 | 5.08 | Gao, 2005 |
| 300 | 15 | 30 | 9 | 5.17 | Gao, 2005 |
| 300 | 15 | 30 | 9 | 5.21 | Gao, 2005 |
| 300 | 15 | 30 | 9 | 5.31 | Gao, 2005 |
| 300 | 15 | 30 | 9 | 5.52 | Gao, 2005 |
| 300 | 15 | 40 | 9 | 0 | Patterson, 1998 |
| 300 | 15 | 40 | 9 | 0.03 | Patterson, 1998 |
| 300 | 15 | 50 | 9 | 3.25 | Patterson, 1998 |
| 300 | 15 | 50 | 9 | 3.91 | Patterson, 1998 |
| 300 | 15 | 55 | 9 | 6.95 | Patterson, 1998 |
| 300 | 15 | 55 | 9 | 7.67 | Patterson, 1998 |
| 300 | 15 | 60 | 9 | 7.71 | Patterson, 1998 |
| 300 | 16 | 25 | 9 | 4.87 | Gao, 2005 |
| 300 | 20 | 20 | 9 | 4.84 | Gao, 2005 |
| 300 | 20 | 40 | 9 | 5.96 | Gao, 2005 |
| 320 | 13 | 25 | 9 | 4.58 | Gao, 2005 |
| 330 | 12 | 30 | 9 | 4.97 | Gao, 2005 |
| 350 | 10 | 21.5 | 9 | 0.07 | Chen, 2006 |
| 350 | 10 | 50 | 9 | 1.87 | Chen, 2006 |
| 350 | 16 | 30 | 9 | 6.17 | Gao, 2005 |
| 400 | 5 | 10 | 4.26 | -0.04 | Tabla, 2012 |
| 400 | 5 | 10 | 5.86 | 0.2 | Tabla, 2012 |
| 400 | 5 | 10 | 6.2 | 0.34 | Tabla, 2012 |
| 400 | 5 | 10 | 3.71 | 0.41 | Tabla, 2012 |
| 400 | 5 | 10 | 6.14 | 3.11 | Tabla, 2012 |
| 400 | 5 | 20 | 9 | 1.62 | Ramos, 2015 |
| 400 | 5 | not reported | 6.19 | 3.29 | Liu, 2017 |
| 400 | 5 | not reported | 6.19 | 3.34 | Liu, 2017 |
| 400 | 5 | not reported | 6.19 | 3.4 | Liu, 2017 |
| 400 | 5 | not reported | 6.19 | 3.43 | Liu, 2017 |
| 400 | 5 | not reported | 6.19 | 3.43 | Liu, 2017 |
| 400 | 5 | not reported | 6.19 | 3.64 | Liu, 2017 |
| 400 | 5 | not reported | 6.19 | 3.7 | Liu, 2017 |
| 400 | 5 | not reported | 6.54 | 4.02 | Liu, 2017 |
| 400 | 10 | 20 | 9 | 2.16 | Ramos, 2015 |
| 400 | 10 | 21.5 | 9 | 0.19 | Chen, 2006 |
| 400 | 10 | 30 | 9 | 5.42 | Gao, 2005 |
| 400 | 10 | 50 | 9 | 3.8 | Chen, 2006 |
| 400 | 15 | 10 | 9 | 0.05 | Patterson, 1998 |
| 400 | 15 | 10 | 9 | 0.46 | Patterson, 1998 |
| 400 | 15 | 20 | 9 | 0.98 | Patterson, 1998 |
| 400 | 15 | 20 | 9 | 1.28 | Patterson, 1998 |
| 400 | 15 | 20 | 9 | 1.95 | Ramos, 2015 |
| 400 | 15 | 20 | 9 | 4.85 | Gao, 2005 |
| 400 | 15 | 40 | 9 | 0.02 | Patterson, 1998 |
| 400 | 15 | 40 | 9 | 0.08 | Patterson, 1998 |
| 400 | 15 | 40 | 9 | 7.18 | Gao, 2005 |
| 400 | 15 | 50 | 9 | 3.45 | Patterson, 1998 |
| 400 | 15 | 50 | 9 | 4.59 | Patterson, 1998 |
| 400 | 20 | 30 | 9 | 6.98 | Gao, 2005 |
| 450 | 10 | 21.5 | 9 | 0.43 | Chen, 2006 |
| 450 | 10 | 50 | 9 | 5.22 | Chen, 2006 |
| 500 | 1 | 50 | 9 | 3.81 | Chen, 2007 |
| 500 | 2 | 50 | 9 | 5.05 | Chen, 2007 |
| 500 | 3 | 4.4 | 7.08 | 1.23 | Kabir, 2021 |
| 500 | 3 | 4.4 | 7.08 | 1.91 | Kabir, 2021 |
| 500 | 3 | 50 | 9 | 4.85 | Chen, 2007 |
| 500 | 3 | 60 | 5.97 | 4.06 | Kabir, 2021 |
| 500 | 3 | 60 | 5.97 | 4.14 | Kabir, 2021 |
| 500 | 4 | 50 | 9 | 5.79 | Chen, 2007 |
| 500 | 5 | 10 | 6.14 | 1.87 | Tabla, 2012 |
| 500 | 5 | 20 | 9 | 1.72 | Ramos, 2015 |
| 500 | 6 | 50 | 9 | 5.99 | Chen, 2007 |
| 500 | 8 | 50 | 9 | 6.52 | Chen, 2007 |
| 500 | 10 | 20 | 9 | 1.55 | Ramos, 2015 |
| 500 | 10 | 21.5 | 9 | 1.09 | Chen, 2006 |
| 500 | 10 | 50 | 9 | 6.56 | Chen, 2007 |
| 500 | 10 | 50 | 9 | 6.61 | Chen, 2006 |
| 500 | 15 | 10 | 9 | 1.64 | Patterson, 1998 |
| 500 | 15 | 10 | 9 | 1.98 | Patterson, 1998 |
| 500 | 15 | 20 | 9 | 2.15 | Patterson, 1998 |
| 500 | 15 | 20 | 9 | 2.46 | Ramos, 2015 |
| 500 | 15 | 20 | 9 | 2.48 | Patterson, 1998 |
| 500 | 15 | 40 | 9 | 0.27 | Patterson, 1998 |
| 500 | 15 | 40 | 9 | 1.15 | Patterson, 1998 |
| 500 | 15 | 50 | 9 | 5.14 | Patterson, 1998 |
| 500 | 15 | 50 | 9 | 6.17 | Patterson, 1998 |
| 550 | 10 | 21.5 | 9 | 2.8 | Chen, 2006 |
| 550 | 10 | 50 | 9 | 7.06 | Chen, 2006 |
| 600 | 1 | 45 | 9 | 1.23 | Guan, 2006 |
| 600 | 2 | 21 | 9 | 2.88 | Guan, 2006 |
| 600 | 2 | 21.5 | 9 | 0.82 | Chen, 2007 |
| 600 | 2 | 45 | 9 | 2.58 | Guan, 2006 |
| 600 | 3 | 45 | 9 | 3 | Guan, 2006 |
| 600 | 4 | 4 | 9 | 4.3 | Guan, 2006 |
| 600 | 4 | 21 | 9 | 4.28 | Guan, 2006 |
| 600 | 4 | 21.5 | 9 | 2.04 | Chen, 2007 |
| 600 | 4 | 45 | 9 | 4 | Guan, 2006 |
| 600 | 5 | 20 | 8 | 0.43 | Patterson, 1995 |
| 600 | 5 | 20 | 9 | 4.13 | Ramos, 2015 |
| 600 | 6 | 21 | 9 | 5.63 | Guan, 2006 |
| 600 | 6 | 21.5 | 9 | 3.21 | Chen, 2007 |
| 600 | 6 | 45 | 9 | 5.97 | Guan, 2006 |
| 600 | 8 | 4 | 9 | 5.65 | Guan, 2006 |
| 600 | 8 | 21 | 9 | 7.26 | Guan, 2006 |
| 600 | 8 | 21.5 | 9 | 3.69 | Chen, 2007 |
| 600 | 8 | 45 | 9 | 8.41 | Guan, 2006 |
| 600 | 10 | 4 | 9 | 7.26 | Guan, 2006 |
| 600 | 10 | 20 | 8 | 1.15 | Patterson, 1995 |
| 600 | 10 | 20 | 9 | 5.24 | Ramos, 2015 |
| 600 | 10 | 21 | 9 | 7.37 | Guan, 2006 |
| 600 | 10 | 21.5 | 9 | 5.39 | Chen, 2007 |
| 600 | 10 | 21.5 | 9 | 6 | Chen, 2006 |
| 600 | 10 | 45 | 9 | 8.36 | Guan, 2006 |
| 600 | 10 | 50 | 9 | 8 | Chen, 2006 |
| 600 | 12 | 21 | 9 | 7.56 | Guan, 2006 |
| 600 | 15 | 10 | 9 | 2.83 | Patterson, 1998 |
| 600 | 15 | 10 | 9 | 7.83 | Patterson, 1998 |
| 600 | 15 | 20 | 8 | 2.19 | Patterson, 1995 |
| 600 | 15 | 20 | 9 | 3.89 | Patterson, 1998 |
| 600 | 15 | 20 | 9 | 5.17 | Patterson, 1998 |
| 600 | 15 | 20 | 9 | 7.47 | Ramos, 2015 |
| 600 | 15 | 21.5 | 9 | 5.59 | Chen, 2007 |
| 600 | 15 | 40 | 9 | 2.62 | Patterson, 1998 |
| 600 | 15 | 40 | 9 | 3.31 | Patterson, 1998 |
| 600 | 15 | 50 | 9 | 7.36 | Patterson, 1998 |
| 600 | 16 | 4 | 9 | 7.35 | Guan, 2006 |
| 600 | 16 | 21 | 9 | 7.81 | Guan, 2006 |
| 600 | 18 | 21 | 9 | 7.84 | Guan, 2006 |
| 600 | 20 | 20 | 8 | 3.45 | Patterson, 1995 |
| 600 | 20 | 21 | 9 | 8.3 | Guan, 2006 |
| 600 | 20 | 21.5 | 9 | 6.35 | Chen, 2007 |
| 600 | 24 | 4 | 9 | 7.6 | Guan, 2006 |
| 600 | 25 | 20 | 8 | 4.32 | Patterson, 1995 |
| 600 | 30 | 4 | 9 | 7.84 | Guan, 2006 |
| 600 | 30 | 20 | 8 | 5.26 | Patterson, 1995 |
| 600 | 30 | 21.5 | 9 | 7.24 | Chen, 2007 |
| 650 | 10 | 21.5 | 9 | 7 | Chen, 2006 |
| 690 | 10 | 21.5 | 9 | 7.65 | Chen, 2006 |
| 700 | 5 | 4 | 7.41 | 3.85 | Syed, 2014 |
| 700 | 5 | 4 | 7.41 | 3.86 | Syed, 2014 |
| 700 | 5 | 4 | 7.41 | 4.14 | Syed, 2014 |
| 700 | 5 | 4 | 7.41 | 4.16 | Syed, 2014 |
| 700 | 5 | 4 | 7.41 | 6.41 | Syed, 2014 |
| 700 | 15 | 10 | 9 | 5.44 | Patterson, 1998 |
| 700 | 15 | 10 | 9 | 7.85 | Patterson, 1998 |
| 700 | 15 | 40 | 9 | 5.69 | Patterson, 1998 |
| 700 | 15 | 40 | 9 | 7.88 | Patterson, 1998 |
| ***Vibrio parahaemolyticus*** | | | | | |
| 100 | 5 | 21.5 | 9 | 0.02 | Chen, 2006 |
| 100 | 10 | 21.5 | 9 | 0.02 | Chen, 2006 |
| 150 | 5 | 21.5 | 9 | 0.14 | Chen, 2006 |
| 150 | 10 | 21.5 | 9 | 0.02 | Chen, 2006 |
| 200 | 5 | 21.5 | 9 | 0.24 | Chen, 2006 |
| 200 | 10 | 21.5 | 9 | 2.08 | Chen, 2006 |
| 250 | 5 | 21.5 | 9 | 2.89 | Chen, 2006 |
| 250 | 10 | 21.5 | 9 | 6.11 | Chen, 2006 |
| 300 | 0.5 | 21.5 | 9 | 0.91 | Chen, 2007 |
| 300 | 1 | 21.5 | 9 | 2.44 | Chen, 2007 |
| 300 | 1.5 | 21.5 | 9 | 4.01 | Chen, 2007 |
| 300 | 2 | 21.5 | 9 | 5.55 | Chen, 2007 |
| 300 | 4 | 21.5 | 9 | 5.75 | Chen, 2007 |
| 300 | 5 | 21.5 | 9 | 6.52 | Chen, 2006 |
| 300 | 6 | 21.5 | 9 | 6.27 | Chen, 2007 |
| 300 | 10 | 21.5 | 9 | 7.08 | Chen, 2007 |
| 300 | 10 | 21.5 | 9 | 7.47 | Chen, 2006 |
| 350 | 5 | 21.5 | 9 | 7.88 | Chen, 2006 |
| 350 | 10 | 21.5 | 9 | 8.88 | Chen, 2006 |
| 200 | 10 | 21.5 | 9 | 0.12 | Chen, 2006 |
| 235 | 15 | 25 | 7 | 0.26 | Nakimbugwe, 2006 |
| 235 | 15 | 25 | 7 | 0.72 | Nakimbugwe, 2006 |
| 235 | 15 | 25 | 7 | 2.05 | Nakimbugwe, 2006 |
| 250 | 10 | 21.5 | 9 | 0.19 | Chen, 2006 |
| 300 | 3 | 22 | 9 | 1.83 | Chen, 2003a |
| 300 | 6 | 22 | 9 | 2.7 | Chen, 2003a |
| 300 | 9 | 22 | 9 | 3.51 | Chen, 2003a |
| 300 | 10 | 21.5 | 9 | 1.98 | Chen, 2006 |
| 300 | 12 | 22 | 9 | 4.45 | Chen, 2003a |
| 300 | 15 | 22 | 9 | 5.06 | Chen, 2003a |
| 300 | 18 | 22 | 9 | 5.54 | Chen, 2003a |
| 300 | 21 | 22 | 9 | 5.62 | Chen, 2003a |
| 300 | 24 | 22 | 9 | 5.95 | Chen, 2003a |
| 300 | 27 | 22 | 9 | 6.15 | Chen, 2003a |
| 300 | 30 | 22 | 9 | 6.19 | Chen, 2003a |
| 300 | 33 | 22 | 9 | 6.62 | Chen, 2003a |
| 300 | 36 | 22 | 9 | 6.86 | Chen, 2003a |
| 300 | 39 | 22 | 9 | 7.15 | Chen, 2003a |
| 300 | 42 | 22 | 9 | 7.15 | Chen, 2003a |
| 300 | 45 | 22 | 9 | 7.85 | Chen, 2003a |
| 300 | 48 | 22 | 9 | 8.48 | Chen, 2003a |
| 350 | 2 | 22 | 9 | 2.02 | Chen, 2003a |
| 350 | 4 | 22 | 9 | 3.13 | Chen, 2003a |
| 350 | 6 | 22 | 9 | 4.52 | Chen, 2003a |
| 350 | 8 | 22 | 9 | 5.37 | Chen, 2003a |
| 350 | 10 | 21.5 | 9 | 5.18 | Chen, 2006 |
| 350 | 10 | 22 | 9 | 0.51 | Chen, 2003a |
| 350 | 11 | 22 | 9 | 5.63 | Chen, 2003a |
| 350 | 13 | 22 | 9 | 6.28 | Chen, 2003a |
| 350 | 15 | 22 | 9 | 6.28 | Chen, 2003a |
| 350 | 17 | 22 | 9 | 7.09 | Chen, 2003a |
| 350 | 19 | 22 | 9 | 7.15 | Chen, 2003a |
| 350 | 20 | 22 | 9 | 1.9 | Chen, 2003a |
| 350 | 21 | 22 | 9 | 7.32 | Chen, 2003a |
| 350 | 24 | 22 | 9 | 7.91 | Chen, 2003a |
| 350 | 26 | 22 | 9 | 8.24 | Chen, 2003a |
| 350 | 28 | 22 | 9 | 8.28 | Chen, 2003a |
| 350 | 30 | 22 | 9 | 2.54 | Chen, 2003a |
| 350 | 40 | 22 | 9 | 3.29 | Chen, 2003a |
| 350 | 50 | 22 | 9 | 4.08 | Chen, 2003a |
| 350 | 60 | 22 | 9 | 5.16 | Chen, 2003a |
| 350 | 70 | 22 | 9 | 5.58 | Chen, 2003a |
| 350 | 80 | 22 | 9 | 5.74 | Chen, 2003a |
| 350 | 90 | 22 | 9 | 6.46 | Chen, 2003a |
| 350 | 100 | 22 | 9 | 7.15 | Chen, 2003a |
| 350 | 110 | 22 | 9 | 7.59 | Chen, 2003a |
| 350 | 120 | 22 | 9 | 8.12 | Chen, 2003a |
| 400 | 1 | 22 | 9 | 1.78 | Chen, 2003a |
| 400 | 2 | 22 | 9 | 3.26 | Chen, 2003a |
| 400 | 3 | 22 | 9 | 4.74 | Chen, 2003a |
| 400 | 4 | 22 | 9 | 5.23 | Chen, 2003a |
| 400 | 5 | 22 | 9 | 0.38 | Chen, 2003a |
| 400 | 5 | 22 | 9 | 5.83 | Chen, 2003a |
| 400 | 6 | 22 | 9 | 6.04 | Chen, 2003a |
| 400 | 7 | 22 | 9 | 6.51 | Chen, 2003a |
| 400 | 8 | 22 | 9 | 6.58 | Chen, 2003a |
| 400 | 9 | 22 | 9 | 6.81 | Chen, 2003a |
| 400 | 10 | 21.5 | 9 | 6.9 | Chen, 2006 |
| 400 | 10 | 22 | 9 | 1.26 | Chen, 2003a |
| 400 | 10 | 22 | 9 | 7.48 | Chen, 2003a |
| 400 | 11 | 22 | 9 | 8.38 | Chen, 2003a |
| 400 | 15 | 22 | 9 | 3.53 | Chen, 2003a |
| 400 | 20 | 22 | 9 | 4.19 | Chen, 2003a |
| 400 | 25 | 22 | 9 | 4.3 | Chen, 2003a |
| 400 | 30 | 22 | 9 | 4.7 | Chen, 2003a |
| 400 | 35 | 22 | 9 | 5.58 | Chen, 2003a |
| 400 | 40 | 22 | 9 | 5.78 | Chen, 2003a |
| 400 | 45 | 22 | 9 | 6.18 | Chen, 2003a |
| 400 | 50 | 22 | 9 | 6.18 | Chen, 2003a |
| 400 | 55 | 22 | 9 | 6.66 | Chen, 2003a |
| 400 | 60 | 22 | 9 | 6.9 | Chen, 2003a |
| 400 | 65 | 22 | 9 | 6.95 | Chen, 2003a |
| 400 | 70 | 22 | 9 | 7.08 | Chen, 2003a |
| 400 | 75 | 22 | 9 | 7.94 | Chen, 2003a |
| 400 | 80 | 22 | 9 | 8.34 | Chen, 2003a |
| 450 | 0.5 | 22 | 9 | 2.87 | Chen, 2003a |
| 450 | 1 | 22 | 9 | 3.96 | Chen, 2003a |
| 450 | 1.5 | 22 | 9 | 5.04 | Chen, 2003a |
| 450 | 2 | 22 | 9 | 1.15 | Chen, 2003a |
| 450 | 2 | 22 | 9 | 5.38 | Chen, 2003a |
| 450 | 2.5 | 22 | 9 | 5.44 | Chen, 2003a |
| 450 | 3 | 22 | 9 | 5.72 | Chen, 2003a |
| 450 | 3.5 | 22 | 9 | 5.91 | Chen, 2003a |
| 450 | 4 | 22 | 9 | 1.04 | Chen, 2003a |
| 450 | 4 | 22 | 9 | 6.11 | Chen, 2003a |
| 450 | 4.8 | 22 | 9 | 6.19 | Chen, 2003a |
| 450 | 5.3 | 22 | 9 | 6.49 | Chen, 2003a |
| 450 | 5.9 | 22 | 9 | 6.51 | Chen, 2003a |
| 450 | 6 | 22 | 9 | 1.64 | Chen, 2003a |
| 450 | 6.4 | 22 | 9 | 6.94 | Chen, 2003a |
| 450 | 7 | 22 | 9 | 7.2 | Chen, 2003a |
| 450 | 7.5 | 22 | 9 | 8.06 | Chen, 2003a |
| 450 | 8 | 22 | 9 | 2.51 | Chen, 2003a |
| 450 | 8 | 22 | 9 |  | Chen, 2003a |
| 450 | 10 | 21.5 | 9 | 7.62 | Chen, 2006 |
| 450 | 10 | 22 | 9 | 3.87 | Chen, 2003a |
| 450 | 12 | 22 | 9 | 4.35 | Chen, 2003a |
| 450 | 14 | 22 | 9 | 4.8 | Chen, 2003a |
| 450 | 16 | 22 | 9 | 5.04 | Chen, 2003a |
| 450 | 18 | 22 | 9 | 5.08 | Chen, 2003a |
| 450 | 20 | 22 | 9 | 5.34 | Chen, 2003a |
| 450 | 22 | 22 | 9 | 5.45 | Chen, 2003a |
| 450 | 24 | 22 | 9 | 5.52 | Chen, 2003a |
| 450 | 26 | 22 | 9 | 5.82 | Chen, 2003a |
| 450 | 28 | 22 | 9 | 5.97 | Chen, 2003a |
| 450 | 30 | 22 | 9 | 5.93 | Chen, 2003a |
| 450 | 32 | 22 | 9 | 6.3 | Chen, 2003a |
| 450 | 34 | 22 | 9 | 6.32 | Chen, 2003a |
| 450 | 36 | 22 | 9 | 6.62 | Chen, 2003a |
| 450 | 38 | 22 | 9 | 7.64 | Chen, 2003a |
| 450 | 40 | 22 | 9 | 7.83 | Chen, 2003a |
| 450 | 42 | 22 | 9 | 8.16 | Chen, 2003a |
| 450 | 44 | 22 | 9 | 8.44 | Chen, 2003a |
| 450 | 46 | 22 | 9 | 8.48 | Chen, 2003a |
| 500 | 1.5 | 22 | 9 | 1.4 | Chen, 2003a |
| 500 | 3 | 22 | 9 | 1.74 | Chen, 2003a |
| 500 | 4.5 | 22 | 9 | 2.87 | Chen, 2003a |
| 500 | 6 | 22 | 9 | 3.22 | Chen, 2003a |
| 500 | 7.5 | 22 | 9 | 3.7 | Chen, 2003a |
| 500 | 9 | 22 | 9 | 4.33 | Chen, 2003a |
| 500 | 10 | 21.5 | 9 | 8.4 | Chen, 2006 |
| 500 | 10.5 | 22 | 9 | 4.67 | Chen, 2003a |
| 500 | 12 | 22 | 9 | 5.25 | Chen, 2003a |
| 500 | 13.5 | 22 | 9 | 5.38 | Chen, 2003a |
| 500 | 15 | 22 | 9 | 5.59 | Chen, 2003a |
| 500 | 16.5 | 22 | 9 | 5.9 | Chen, 2003a |
| 500 | 18 | 22 | 9 | 5.99 | Chen, 2003a |
| 500 | 19.5 | 22 | 9 | 5.94 | Chen, 2003a |
| 500 | 21 | 22 | 9 | 6.57 | Chen, 2003a |
| 500 | 22.5 | 22 | 9 | 6.53 | Chen, 2003a |
| 500 | 24 | 22 | 9 | 6.7 | Chen, 2003a |
| 500 | 25.5 | 22 | 9 | 6.95 | Chen, 2003a |
| 500 | 27 | 22 | 9 | 7.28 | Chen, 2003a |
| 500 | 28.5 | 22 | 9 | 7.45 | Chen, 2003a |
| 500 | 30 | 22 | 9 | 8.23 | Chen, 2003a |
| 500 | 31.5 | 22 | 9 | 8.54 | Chen, 2003a |
| 550 | 10 | 21.5 | 9 | 8.16 | Chen, 2006 |
| ***Yersinia pseudotuberculosis*** | | | | | |
| 300 | 2 | 10 | 7.1 | 0.25 | Schlesser, 2009 |
| 300 | 2 | 25 | 7.1 | 0 | Schlesser, 2009 |
| 300 | 4 | 10 | 7.1 | 1.05 | Schlesser, 2009 |
| 300 | 4 | 25 | 7.1 | 3.35 | Schlesser, 2009 |
| 300 | 6 | 10 | 7.1 | 1.1 | Schlesser, 2009 |
| 300 | 6 | 25 | 7.1 | 7.1 | Schlesser, 2009 |
| 500 | 2 | 10 | 7.1 | 7.1 | Schlesser, 2009 |
| 500 | 2 | 25 | 7.1 | 7.1 | Schlesser, 2009 |
| 500 | 4 | 10 | 7.1 | 7.1 | Schlesser, 2009 |
| 500 | 4 | 25 | 7.1 | 7.1 | Schlesser, 2009 |
| 500 | 6 | 10 | 7.1 | 7.1 | Schlesser, 2009 |
| 500 | 6 | 25 | 7.1 | 7.1 | Schlesser, 2009 |

References:

Allison, A., Chowdhury, S., & Fouladkhah, A. (2018). Synergism of mild heat and high-pressure pasteurization against *Listeria monocytogenes* and natural microflora in phosphate-buffered saline and raw milk. *Microorganisms*, *6*(4), 102. CABDirect. https://doi.org/10.3390/microorganisms6040102

Bulut, S. (2014). Inactivation of *Escherichia coli* in milk by high pressure processing at low and subzero temperatures*. High Pressure Research:34*(4), 439–446.

<https://doi.org/10.1080/08957959.2014.981262>

Buzrul, S., Alpas, H., Largeteau, A., & Demazeau, G. (2009). Efficiency of pulse pressure treatment for inactivation of *Escherichia coli* and *Listeria innocua* in whole milk. *European Food Research and Technology*, *229*(1), 127–131. https://doi.org/10.1007/s00217-009-1033-0

Chen, H., & Hoover, D. G. (2003a). Pressure inactivation kinetics of *Yersinia enterocolitica* ATCC 35669. *International Journal of Food Microbiology*, *87*(1–2), 161–171. https://doi.org/10.1016/s0168-1605(03)00064-3

Chen, H. Q., & Hoover, D. G. (2003b). Modeling the combined effect of high hydrostatic pressure and mild heat on the inactivation kinetics of *Listeria monocytogenes* Scott A in whole milk. *Innovative Food Science & Emerging Technologies*, *4*(1), 25–34. CABDirect. https://doi.org/10.1016/S1466-8564(02)00083-8

Chen, H. Q., & Hoover, D. G. (2004). Use of Weibull model to describe and predict pressure inactivation of Listeria monocytogenes Scott A in whole milk. *Innovative Food Science & Emerging Technologies*, *5*(3), 269–276. CABDirect. https://doi.org/10.1016/j.ifset.2004.03.002

Chen, H., Guan, D., & Hoover, D. G. (2006). Sensitivities of foodborne pathogens to pressure changes. *Journal of Food Protection*, *69*(1), 130–136. <https://doi.org/10.4315/0362-028x-69.1.130>

Chen, H. Q. (2007). Use of linear, Weibull, and log-logistic functions to model pressure inactivation of seven foodborne pathogens in milk. *Food Microbiology*, *24*(3), 197–204. CABDirect.

Dogan, C., & Erkmen, O. (2004). High pressure inactivation kinetics of *Listeria monocytogenes* inactivation in broth, milk, and peach and orange juices. *Journal of Food Engineering*, *62*(1), 47–52. https://doi.org/10.1016/S0260-8774(03)00170-5

Durães-Carvalho, R., Souza, A. R., Martins, L. M., Sprogis, A. C. S., Bispo, J. A. C., Bonafe, C. F. S., & Yano, T. (2012). Effect of high hydrostatic pressure on *Aeromonas hydrophila* AH 191 growth in milk. *Journal of Food Science*, *77*(8), M417-424. https://doi.org/10.1111/j.1750-3841.2012.02819.x

Erkmen, O., & Karataş, Ş. (1997). Effect of high hydrostatic pressure on *Staphylococcus aureus* in milk. *Journal of Food Engineering*, *33*(3), 257–262. https://doi.org/10.1016/S0260-8774(97)00021-6

Erkmen, O., & Dogan, C. (2004). Effects of ultra high hydrostatic pressure on *Listeria monocytogenes* and natural flora in broth, milk and fruit juices. *International Journal of Food Science & Technology*, *39*(1), 91–97. https://doi.org/10.1046/j.0950-5423.2003.00754.x

Erkmen, O. (2011). Effects of high hydrostatic pressure on Salmonella typhimurium and aerobic bacteria in milk and fruit juices. *Rom. Biotechnol. Lett. 16*(5),

6540-6547.

Foster, D. M., Poulsen, K. P., Sylvester, H. J., Jacob, M. E., Casulli, K. E., & Farkas, B. E. (2016). Effect of high-pressure processing of bovine colostrum on immunoglobulin G concentration, pathogens, viscosity, and transfer of passive immunity to calves. *Journal of Dairy Science*, *99*(11), 8575–8588. CABDirect. https://doi.org/10.3168/jds.2016-11204

Gao, Y.-L., Wang, Y.-X., & Jiang, H.-H. (2005). Effect of high pressure and mild heat on *Staphylococcus aureus* in milk using response surface methodology. *Process Biochemistry*, *40*(5), 1849–1854. https://doi.org/10.1016/j.procbio.2004.06.053

García Graells, C., Masschalck, B., & Michiels, C. W. (1999). Inactivation of *Escherichia coli* in milk by high-hydrostatic-pressure treatment in combination with antimicrobial peptides. *Journal of Food Protection*, *62*(11), 1248–1254. CABDirect.

García-Graells, C., Valckx, C., & Michiels, C. W. (2000). Inactivation of *Escherichia coli* and *Listeria innocua* in milk by combined treatment with high hydrostatic pressure and the lactoperoxidase system. *Applied and Environmental Microbiology*, *66*(10), 4173–4179. https://doi.org/10.1128/AEM.66.10.4173-4179.2000

Guan, D. S., Chen, H. Q., & Hoover, D. G. (2005). Inactivation of S*almonella typhimurium* DT 104 in UHT whole milk by high hydrostatic pressure. *International Journal of Food Microbiology*, *104*(2), 145–153. CABDirect. https://doi.org/10.1016/j.ijfoodmicro.2005.01.014

Guan, D. S., Chen, H. Q., Ting, E. Y., & Hoover, D. G. (2006). Inactivation of *Staphylococcus aureus* and *Escherichia coli* O157:H7 under isothermal-endpoint pressure conditions. *Journal of Food Engineering*, *77*(3), 620–627. CABDirect. https://doi.org/10.1016/j.jfoodeng.2005.07.021

Hayman, M. M., Anantheswaran, R. C., & Knabel, S. J. (2007). The effects of growth temperature and growth phase on the inactivation of *Listeria monocytogenes* in whole milk subject to high pressure processing. *International Journal of Food Microbiology*, *115*(2), 220–226. CABDirect. https://doi.org/10.1016/j.ijfoodmicro.2006.10.019

Hayman, M. M., Anantheswaran, R. C., & Knabel, S. J. (2008). Heat shock induces barotolerance in *Listeria monocytogenes*. *Journal of Food Protection*, *71*(2), 426–430. CABDirect.

Huang, H.-W., Lung, H.-M., Chang, Y.-H., Yang, B. B., & Wang, C.-Y. (2015). Inactivation of pathogenic *Listeria monocytogenes* in raw milk by high hydrostatic pressure. *Foodborne Pathogens and Disease*, *12*(2), 139–144. https://doi.org/10.1089/fpd.2014.1871

Kabir, M. N., Aras, S., George, J., Wadood, S., Chowdhury, S., & Fouladkhah, A. C. (2021). High-pressure and thermal-assisted pasteurization of habituated, wild-type, and pressure-stressed *Listeria monocytogenes*, *Listeria innocua*, and *Staphylococcus aureus*. *LWT - Food Science and Technology*, *137*. CABDirect. <https://doi.org/10.1016/j.lwt.2020.110445>

Karatzas, K.A.G. & Bennik, M.H.J. (2002). Characterization of a *Listeria monocytogenes* Scott A Isolate with High Tolerance towards High Hydrostatic

Pressure. *Appl Environ Microbiol* 68. <https://doi.org/10.1128/AEM.68.7.3183-3189.2002>

Komora, N., Maciel, C., Pinto, C. A., Ferreira, V., Brandão, T. R. S., Saraiva, J. M. A., Castro, S. M., & Teixeira, P. (2020). Non-thermal approach to *Listeria monocytogenes* inactivation in milk: The combined effect of high pressure, pediocin PA-1 and bacteriophage P100. *Food Microbiology*, *86*, 103315. <https://doi.org/10.1016/j.fm.2019.103315>

Koseki, S., Mizuno, Y., & Yamamoto, K. (2008). Use of mild-heat treatment following high-pressure processing to prevent recovery of pressure-injured *Listeria monocytogenes* in milk. *Food Microbiology*, *25*(2), 288–293. <https://doi.org/10.1016/j.fm.2007.10.009>

Li, Y., Zheng, Z., Zhu, S., Ramaswamy, H. S., & Yu, Y. (2020). Effect of Low-Temperature-High-Pressure Treatment on the Reduction of *Escherichia coli* in Milk. *Foods (Basel, Switzerland)*, *9*(12). https://doi.org/10.3390/foods9121742

Liu, H.-B., Li, P., Sun, C., Du, X.-J., Zhang, Y., & Wang, S. (2017). Inhibitor-Assisted High-Pressure Inactivation of Bacteria in Skim Milk. *Journal of Food Science*, *82*(7), 1672–1681. https://doi.org/10.1111/1750-3841.13737

Martínez‐Rodriguez, A., & Mackey, B. M. (2005). Factors affecting the pressure resistance of some *Campylobacter* species. *Letters in Applied Microbiology*, *41*(4), 321–326. https://doi.org/10.1111/j.1472-765X.2005.01768.x

McClements, J. M. J., Patterson, M. F., & Linton, M. (2001). The Effect of Growth Stage and Growth Temperature on High Hydrostatic Pressure Inactivation of Some Psychrotrophic Bacteria in Milk. *Journal of Food Protection*, *64*(4), 514–522. <https://doi.org/10.4315/0362-028X-64.4.514>

Mishra, N., Puri, V., & Demirci, A. (2013). Inactivation and Injury of *Listeria monocytogenes* under Combined Effect of Pressure and Temperature in UHT Whole Milk. *JOURNAL OF FOOD PROCESS ENGINEERING*, *36*(3), 374–384. https://doi.org/10.1111/jfpe.12004

Misiou, O., van Nassau, T. J., Lenz, C. A., & Vogel, R. F. (2018). The preservation of *Listeria*-critical foods by a combination of endolysin and high hydrostatic pressure. *International Journal of Food Microbiology*, *266*, 355–362. https://doi.org/10.1016/j.ijfoodmicro.2017.10.004

Nakimbugwe, D., Masschalck, B., Anim, G., & Michiels, C. W. (2006). Inactivation of gram-negative bacteria in milk and banana juice by hen egg white and lambda lysozyme under high hydrostatic pressure. *International Journal of Food Microbiology*, *112*(1), 19–25. https://doi.org/10.1016/j.ijfoodmicro.2006.06.010

Park, S. H., Hong, G. P., Min, S. G., & Choi, M. J. (2009). Effects of pressure assisted mild thermal treatment on inactivation of *Eescherichia coli* ATCC 10536 iIn milk suspension. *Korean Journal for Food Science of Animal Resources*, *29*(3), 310–316. https://doi.org/10.5851/kosfa.2009.29.3.310

Patterson, M. F., Quinn, M., Simpson, R., & Gilmour, A. (1995). Sensitivity of vegetative pathogens to high hydrostatic pressure treatment in phosphate-buffered saline and foods. *Journal of Food Protection*, *58*(5), 524–529. CABDirect.

Patterson, M. F., & Kilpatrick, D. J. (1998). The combined effect of high hydrostatic pressure and mild heat on inactivation of pathogens in milk and poultry. *Journal of Food Protection*, *61*(4), 432–436. CABDirect.

Ramaswamy, H. S., Jin, H., & Zhu, S. (2009). Effects of fat, casein and lactose on high-pressure destruction of *Escherichia coli* K12 (ATCC-29055) in milk. *Food and Bioproducts Processing*, *87*(1), 1–6. https://doi.org/10.1016/j.fbp.2008.02.005

Ramos, S. J., Chiquirrín, M., García, S., Condón, S., & Pérez, M. D. (2015). Effect of high pressure treatment on inactivation of vegetative pathogens and on denaturation of whey proteins in different media. *LWT - Food Science and Technology*, *63*(1), 732–738. <https://doi.org/10.1016/j.lwt.2015.03.085>

Schlesser, J. E., & Parisi, B. (2009). Inactivation of *Yersinia pseudotuberculosis* 197 and *Francisella tularensis* LVS in beverages by high pressure processing. *Journal of Food Protection*, *72*(1), 165–168. https://doi.org/10.4315/0362-028x-72.1.165

Shearer, A. E. H., Neetoo, H. S., & Chen, H. Q. (2010). Effect of growth and recovery temperatures on pressure resistance of *Listeria monocytogenes*. *International Journal of Food Microbiology*, *136*(3), 359–363. CABDirect. https://doi.org/10.1016/j.ijfoodmicro.2009.10.034

Simpson, R. K., & Gilmour, A. (1997). The resistance of *Listeria monocytogenes* to high hydrostatic pressure in foods. *Food Microbiology*, *14*(6), 567–573. CABDirect. https://doi.org/10.1006/fmic.1997.0117

Solomon, E. B., & Hoover, D. G. (2004). Inactivation of *Campylobacter jejuni* by high hydrostatic pressure. *Letters in Applied Microbiology*, *38*(6), 505–509. https://doi.org/10.1111/j.1472-765X.2004.01527.x

Stratakos, A. Ch., Inguglia, E. S., Linton, M., Tollerton, J., Murphy, L., Corcionivoschi, N., Koidis, A., & Tiwari, B. K. (2019). Effect of high pressure processing on the safety, shelf life and quality of raw milk. *Innovative Food Science & Emerging Technologies*, *52*, 325–333. https://doi.org/10.1016/j.ifset.2019.01.009

Styles, M. F., Hoover, D. G., & Farkas, D. F. (1991). Response of *Listeria monocytogenes* and *Vibrio parahaemolyticus* to high hydrostatic pressure. *Journal of Food Science*, *56*(5), 1404–1407. CABDirect. https://doi.org/10.1111/j.1365-2621.1991.tb04784.x

Syed, Q. A., Buffa ,Martin, Guamis ,Buenaventura, & and Saldo, J. (2013). Lethality and injuring the effect of compression and decompression rates of high hydrostatic pressure on *Escherichia coli* O157:H7 in different matrices. *High Pressure Research*, *33*(1), 64–72. https://doi.org/10.1080/08957959.2013.767898

Syed, Q. A., Buffa, M., Guamis, B., & Saldo, J. (2014). Effect of Compression and Decompression Rates of High Hydrostatic Pressure on Inactivation of *Staphylococcus aureus* in Different Matrices. *Food and Bioprocess Technology*, *7*(4), 1202–1207. https://doi.org/10.1007/s11947-013-1146-0

Tabla, R., Martínez, B., Rebollo, J. E., González, J., Ramírez, M. R., Roa, I., Rodríguez, A., & García, P. (2012). Bacteriophage performance against *Staphylococcus aureus* in milk is improved by high hydrostatic pressure treatments. *International Journal of Food Microbiology*, *156*(3), 209–213. https://doi.org/10.1016/j.ijfoodmicro.2012.03.023

Wen, J., Anantheswaran, R. C., & Knabel, S. J. (2009). Changes in barotolerance, thermotolerance, and cellular morphology throughout the life cycle of *Listeria monocytogenes*. *Applied and Environmental Microbiology*, *75*(6), 1581–1588. CABDirect. https://doi.org/10.1128/AEM.01942-08
